# Screening differentially expressed proteins from co-cultured hematopoietic cells and bone marrow-derived stromal cells by quantitative proteomics (SILAC) method

**DOI:** 10.1186/s12014-019-9249-x

**Published:** 2019-07-18

**Authors:** Rui Liu, Yi Wang, Bingxin Li, Hui Wang, Feng Guan, Zengqi Tan, Xiang Li

**Affiliations:** 1 Joint International Research Laboratory of Glycobiology and Medicinal Chemistry, College of Life Science, Northwest University, 229 Taibai North Road, Xi’an, 710069 Shaanxi China; 2 Department of Hematology, Provincial People’s Hospital, Xi’an, Shaanxi China; 3 Wuxi School of Medicine, Jiangnan University, Wu’xi, China

**Keywords:** Hematopoietic cells, Stromal cells, Co-culture, Proteomics, SILAC method

## Abstract

**Background:**

Bone marrow stromal cells protect hematopoietic cells and provide drug resistance by delivering bunch of variable proteins. Thus, alterations of protein expression are typically associated with cell–cell signal transduction and regulation of cellular functions.

**Methods:**

Co-culture models of bone marrow stromal cells and hematopoietic cells are often used in studies of their crosstalk. Studies of altered protein expression initiated by stromal cell/hematopoietic cell interactions are an important new trend in microenvironmental research. There has been no report to date of global quantitative proteomics analysis of crosstalk between hematopoietic cells and stromal cells. In this study, we analyzed quantitative proteomes in a co-culture system of stromal HS5 cells and hematopoietic KG1a cells, and simultaneously tracked differentially expressed proteins in two types of cells before and after co-culture by stable isotope labeling by amino acids in cell culture (SILAC) method.

**Results:**

We have shown that in co-cultured KG1a, 40 proteins (including CKAP4, LMNA, and SERPINB2) were upregulated and 64 proteins (including CD44, CD99, and NCAM1) were downregulated relative to KG1a alone. We utilized IPA analysis to discover that the NOD-like receptor signaling pathway was upregulated, whereas platelet activation was downregulated in co-cultured KG1a cells. Furthermore, 95 proteins (including LCP1, ARHGAP4, and UNCX) were upregulated and 209 proteins (including CAPG, FLNC, and MAP4) were downregulated in co-cultured HS5 relative to HS5 alone. The tight junction pathway was downregulated and glycolysis/gluconeogenesis pathway was dysfunctional in co-cultured HS5. Most importantly, the significantly differentially expressed proteins can also be confirmed using different co-cultured cell lines.

**Conclusion:**

Altogether, we recommend such quantitative proteomics approach for the studies of the hematopoietic–stroma cross-talk, differentially expressed proteins and related signaling pathways identification. The differentially expressed proteins identified from this current SILAC method will provide a useful basis for ongoing studies of crosstalk between stromal cells and hematopoietic cells in co-culture systems. All these result suggested our ongoing studies can focus on the mechanisms underlying CKAP4 increase and CD44 decrease in co-cultured hematopoietic cells, and the increase of LCP1 and decrease of CAPG in co-cultured stromal cell. The proteomic profiles from the KG1a/stromal cell co-culture system give new molecular insights into the roles of these cells in MDS pathophysiology and related bone disease.

**Electronic supplementary material:**

The online version of this article (10.1186/s12014-019-9249-x) contains supplementary material, which is available to authorized users.

## Introduction

Bone marrow stroma cells can create a niche, which determines the proliferation, differentiation, or quiescence of hematopoietic stem cells (HSC) as well as regulates the residence of surrounding tumor cells [1]. There is increasing evidence that the bone marrow microenvironment generates signals essential in determining and controlling normal hematopoiesis [1–3]. For example, clonal cells from myelodysplastic syndrome (MDS) and their bone marrow stromal cells interact and have mutual impacts on each other’s biology [4]. Abnormal secretion by bone marrow stromal cells of cytokines such as tumor necrosis factor alpha (TNF-α) and interleukin-32 can result in malignant hematopoiesis syndromes, e.g., MDS [5–7]. Modification of stroma cells was found in different types of leukemias, creating a cancer supportive mircroenvrionment and promoting leukemogenesis and resistance to treatment [8–10]. The absence of normal hematopoiesis in MDS patients may thus alter the bone marrow microenvironment and promote niches that favor propagation of clonal cells [11, 12].

Thus, to mimic the bone marrow conditions, various in vitro co-culture models, particularly human bone marrow stromal cells co-cultured with hematopoietic cells, have been used to evaluate crosstalk between clonal/tumor cells and the bone marrow niche [13–16]. Bone marrow stromal cell lines HS5 and HS27a represent two major types of cells related to early cancer development and are useful for deciphering cross talk between stromal cells and clonal cells. HS27a supports “cobblestone area” formation by early hematopoietic progenitor cells, whereas HS5 secretes multiple cytokines that support proliferation of committed progenitor cells [17]. In our previous studies, we integrated information on quantitative proteomic data with genomic data to discover several differentially expressed proteins in two bone marrow stromal cells HS5 and HS27a [18]. The SILAC method has many advantages to reduce sample to sample variability therefore is highly suitable for protein quantification [19, 20]. The cross-talk between hematopoietic cells and stromal cells leads to a rearrangement of each other’s functions, so it is very crucial to set up the quantitative proteomic setting to monitor the differentially expressed proteins in both cell populations. In the present study, we used the SILAC method to examine a model system of KG1a co-cultured with HS5. We identified several differentially expressed proteins in the two types of cells, annotated functions of these proteins using GO, KEGG, and interconnecting networks. The SILAC data can also be validated using different co-cultured cells. Altogether, the SILAC method described here is recommended to study the crosstalk in co-culture models. And the confirmed differentially expressed proteins from different co-culture models will provide potential interests to study their functional roles in the ongoing works.

## Materials and methods

### Cell lines and primary CD34+ cells

Hematopoietic KG1a cells (derived from acute myeloid leukemia) and bone marrow stromal cell lines HS5 and HS27a were grown, propagated, and subjected to experiments between passages 8 and 24, as described previously [13]. The human MDS cell line SKM-1 cells were obtained from Prof. Xiao Li’s lab (Shanghai Jiaotong University Sixth People’s Hospital, Shanghai, China). Above cells were cultured in RPMI 1640 (Biological Industries, Kibutz Beit Haemek, Israel) with 10% fetal bovine serum (Biological Industries, Kibutz Beit Haemek, Israel). Primary CD34+ cord blood cells were derived from the cord blood (CB) of healthy babies from the People’s Hospital of Shaanxi Province. Blood was aseptically aspirated from placenta and umbilical CB veins immediately following normal obstetrical deliveries and placed into blood bags containing an anticoagulant (citrate, phosphate, dextrose, and adenine-1). Samples were processed within 8 h of collection. CD34+ cells were cultured in StemSpan™ SFEM II (StemCell Technologies, Canada) added with StemSpan™ Megakaryocyte Expansion Supplement (StemCell Technologies, Canada). Informed consent was obtained from all individual participants included in the study.

### Co-culture model

For co-culture study, KG1a or SKM-1cells were co-cultured with either HS5 or HS27a overnight. After 24-h incubation, KG1a cells or SKM-1 cells were sorted from co-culture, and related proteins expression was determined by Western blotting. Normal primary CD34^+^ cells were co-cultured with HS5 overnight. After 24-h incubation, Normal primary CD34^+^ cells were sorted from co-culture, and related proteins expression was determined by Western blotting.

### SILAC labeling method

For SILAC labeling, HS5 cells were cultured in SILAC-labeled RPMI 1640 medium with 10% dialyzed FBS and 1% penicillin/streptomycin containing “light” (K0R0) or “heavy” (K8R10) lysine (Lys) and arginine (Arg), while KG1a cells were cultured under the same conditions except with “medium” (K4R6) or “heavy” (K8R10) Lys and Arg [21]. l-Proline (200 mg/L) was added to the medium to prevent Arg-to-proline conversion [22, 23]. SILAC-labeled cells were cultured in SILAC medium for at least 6 passages to eliminate native Lys and Arg.

### Cell sorting

Cell pellets were washed with 10 mL Miltenyi buffer, centrifuged at 1200 rpm for 5–7 min, and resuspended in 300 μL buffer per 1 × 10^8^ cells, added with CD45^+^ microbeads (100 μL per 10^8^ cells), and incubated for 30 min at 4 °C. Cells were washed with Miltenyi buffer, resuspended in 2 mL Miltenyi buffer, and separated by autoMACS separator. CD45^+^ cells (mainly KG1a or SKM-1) and CD34^+^ cells (Normal primary CD34^+^ hematopoietic stem cells) were isolated by magnetic-activated cell sorting (MACS; Miltenyi Biotec; Auburn, CA, USA), resulting in 95–98% purity as determined by flow cytometry. CD45- cells and CD34- were mainly HS5or HS27a.

### Protein digestion and fractionation

HS5 and KG1a cells were lysed in buffer (7 M urea and 2 M thiourea in 50 mM ammonium bicarbonate) and sonicated (Scientz; Ningbo, China). Lysates were centrifuged at 15,000 × *g* for 15 min. Protein concentration of supernatant was determined by Bradford assay (Pierce Biotechnology; Rockford, IL, USA). Heavy- and light-labeled HS5 lysates, and heavy- and medium-labeled KG1a lysates, were each mixed at ratio 1:1. Proteins (1 mg) were digested overnight with Lys C and trypsin as per FASP protocol [24]. Peptides were recovered from the filter, desalted and fractionated with Oasis HLB 1-mL cartridges (Waters; Milford, MA, USA), loaded three times onto Oasis HLB, and eluted successively with 7.5%, 10%, 12.5%, 15%, 17.5%, 25%, and 60% acetonitrile (ACN) in 50 mM ammonium bicarbonate. Flow-through from cartridges was desalted with 1 mL Sep-Pak C18 (Waters). Fractions were lyophilized in a vacuum centrifuge and subjected to LC–MS/MS as below.

### Nanoflow LC–MS/MS analysis

Two independent replicates were dissolved in loading buffer (0.1% formic acid) and loaded onto a 20-cm capillary column packed in-house with 3-μm Reprosil-Pur C18 beads (Dr. Maisch; Ammerbuch, Germany) using an EASY-nLC 1000 system (Thermo Scientific; San Jose, CA, USA). Running buffer A was 0.1% formic acid in water; running buffer B was 0.1% formic acid in ACN. Total gradient was 120 min, with flow rate started at 300 nL/min. Detailed gradient was 6% ACN with linear increase to 30% ACN over 105 min, followed by 4 min linear increase to 90% ACN. MS data were acquired using data-dependent top-20 method on Q Exactive HF (Thermo Scientific). Analytical parameters were: spray voltage 2 kV; S-lens RF level 60; capillary temperature 275 °C; full-scan resolutions 60,000@m/z 200 with AGC 3e6, maximum fill time 20 ms, and mass range of full mass 350–1500; MS^2^ scan resolutions 15,000@m/z 200 with AGC 5e4 and maximum fill time 100 ms for proteomics analysis; isolation width 1.6 Th; fixed first mass 110; normalized collision energy 27; peptide match set to “preferred”; isotope exclusion on. Precursor ions with single, unassigned charge states were eliminated from fragmentation selection.

### Data analysis

Data were analyzed using the MaxQuant software package, V. 1.5.1.12, with Andromeda search engine [24]. False discovery rate (FDR) was set at 1% for proteins and peptides. For peptides, minimum length six amino acids and maximum mass 10,000 Da were required. Fragmentation spectra were searched by Andromeda using the UniProt human database (V. 201502; 90,300 entries) combined with 262 common contaminants. Enzyme specificity was set as C-terminal to Arg and Lys and a maximum of two missed cleavages. Second peptide search was enabled. Carbamidomethylation (C) was set as fixed modification. Deamidation (NQ) and oxidation (M) were set as variable modifications. SILAC quantification was set to doublets. For KG1a data, Lys (+ 4 Da) and Arg (+ 6 Da) were selected as medium labels, and Lys (+ 8 Da) and Arg (+ 10 Da) were selected as heavy labels. For HS5 data, Lys (+ 0 Da) and Arg (+ 0 Da) were selected as light labels, and Lys (+ 8 Da) and Arg (+ 10 Da) were selected as heavy labels.

Differential regulation was considered using log2 ratio-cut off together with z-score cut-off, as described previously [25,26]. In brief, H/X (L or M) (ratio of “heavy” to “light” or “medium”) were converted into log2 space to determine geometric means, and average ratios and standard deviations were determined for each data set. The log2 H/X ratio for each protein was converted to a z-score using the following formula:

$$ z\,scores\,\left( \sigma \right) \,of\, \left[ b \right] = \frac{{\log_{2} \frac{H}{{X\, \left( {M\, or\, L} \right)}}\left[ b \right] - Average\, of\,(\log_{2} \,of\, each\, number,\, a, \ldots ,n)}}{{Standard\, deviation\, of\, (\log_{2} \,of\, each\, number,\, a, \ldots ,n)}} $$

where b represents a single protein in a data set population (a, …, n). The z-score indicates the number of standard deviation units (σ) the protein’s log_2_ H/X ratio is away from the population mean. Thus, a z-score ≥ 1.960σ indicates that the protein’s differential expression lies outside the 95% confidence interval, ≥ 2.576σ indicates that differential expression is outside the 99% confidence interval, and ≥ 3.291σ indicates 99.9% confidence. A protein with z-score ≥ 1.960σ and |log2 ratio| of > 0.58 was considered significantly differentially expressed. Extracted data from the two experiments were subjected to further functional analysis.

### Functional annotation

Proteins showing significant changes (|log2 ratio| of > 0.58 and z-score ≥ 1.960σ) in UniProt identifiers were uploaded to the Protein Center database (Thermo Scientific). GO enrichment analysis and KEGG (Kyoto Encyclopedia of Genes and Genomes) pathways analysis of these proteins were performed. KEGG pathways with FDR p-value < 0.05 were considered significant.

To assess interactions, proteins with significant changes containing UniProt identifiers and corresponding expression values were uploaded to the Ingenuity Systems Application (IPA) (www.ingenuity.com). Networks of these proteins were generated algorithmically based on their connectivity. Fisher’s exact test was used to calculate p-values indicating the probability of a particular biological function or disease belonging to a particular network.

### Western blotting

Total cell proteins were prepared and quantified by BCA assay (Beyotime; Haimen, China), equal amounts of protein (20 µg) from each lysate were separated by 7.5% SDS-PAGE, and proteins were transferred to PVDF membranes for immunoblotting. Membranes were blocked with 5% nonfat dry milk diluted in Tris-buffered saline containing 0.1% Tween-20 (TBS-T) for 1 h at room temperature, and incubated overnight at 4 °C in 5% nonfat dry milk/TBS-T containing anti-CKAP4, anti-CD44, anti-LCP1, or anti-CAPG (Abcam; Cambridge, MA, USA) rabbit antibody. Secondary goat anti-rabbit antibodies (Santa Cruz Biotechnology; Santa Cruz, CA, USA) conjugated to horseradish peroxidase were used for enhanced chemiluminescence (Pierce Biotechnology; Chicago, IL, USA), and membranes were exposed to film.

### CD44 knockdown and CCK8 assay

Depletion of CD44 was performed using GenePharma siRNA. Transfections at a final concentration of 400 nM GenePharma siRNA with Lipofectamine^®^ 2000 Reagent (Life Technologies, ThermoFisher Distributer; Brendale QLD, Australia) were performed per the manufacturer’s instructions. Incubate the cells at 37 °C in a CO2 incubator for 24–96 h until to assay for gene knockdown. Following is GenePharma siRNA sequences for CD44: Scramble RNA (5′-UUCUCCGAACGUGUCACGUTT-3′), siRNA#1 (5′-UUCUUCGACUGUUGACUGCTT-3′), siRNA#2 (5′-AUAUGUGUCAUACUGGGAGTT-3′), siRNA#3 (5′-UAGUUAUGGUAAUUGGUCCTT-3′). The cell suspension (100 μL/well) was seeded in a 96-well plate. 10 μL of CCK-8 solution was added to each well of the plate, and the plate was incubated for 4 h, and the absorbance at 450 nm was measured using a microplate reader.

## Results

### Experimental design

**Fig. 1:**
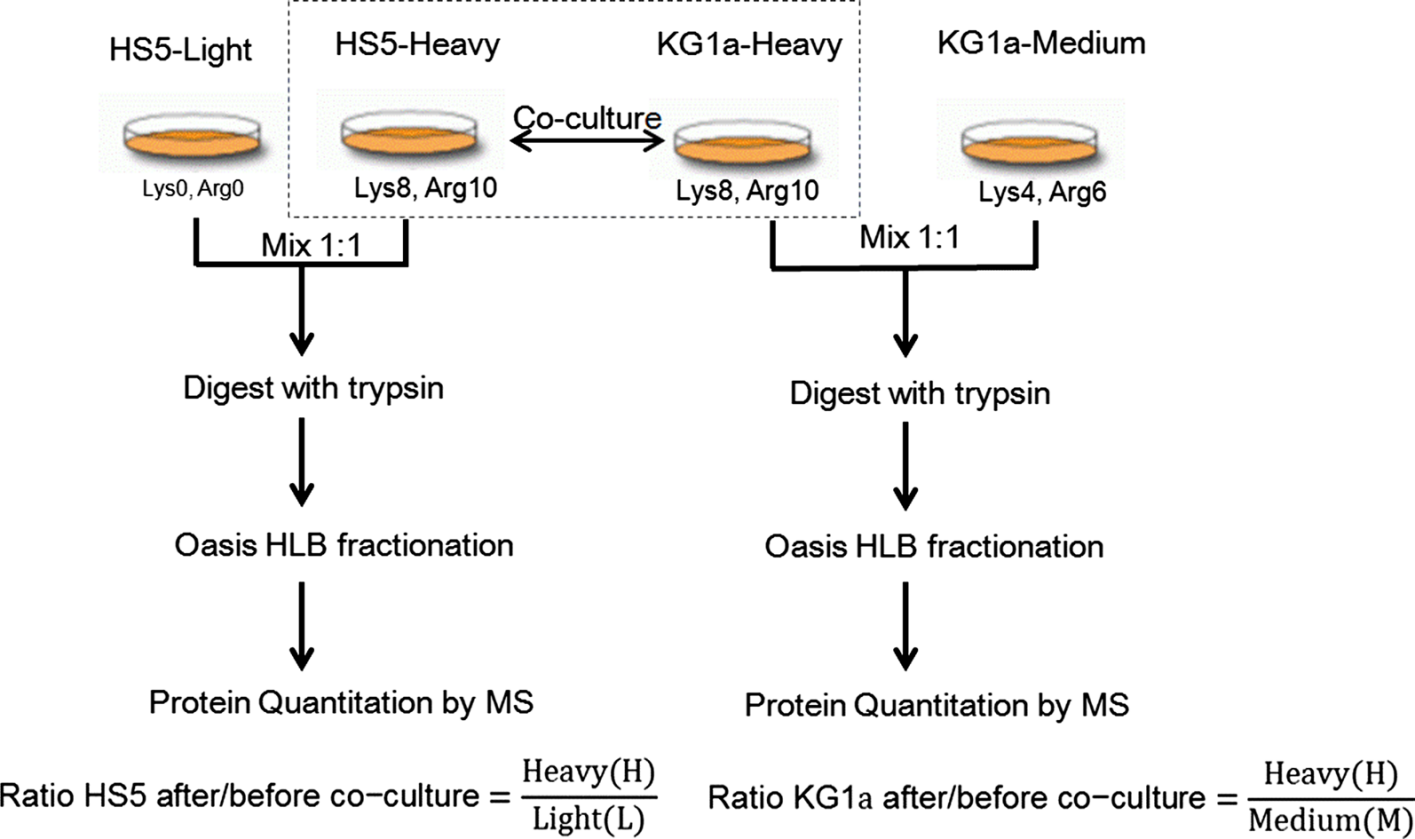
Experiment design for proteome quantification of KG1a and HS5 cell w/o co-culture. The data were collected by two independent replicates

KG1a cells were labeled with K8R10 (heavy channel) or K4R10 (medium channel), and HS5 cells were labeled with K8R10 (heavy channel) or K0R0 (light channel), as described in M&M/ "Co-culture model”. Heavy-labeled HS5 were co-cultured with heavy-labeled KG1a and then separated by AutoMACS. To obtain a profile of proteins in bone marrow-derived stromal cells before and after contact with KG1a, light-labeled and heavy-labeled HS5 cells, respectively, were mixed at ratio 1:1 and subjected to quantitative proteomics analysis. To investigate differentially expressed proteins in KG1a in the presence and absence of stromal cells, heavy-labeled and medium-labeled KG1a cells, respectively, were mixed at ratio 1:1 and subjected to quantitative proteomics analysis (Fig. 1). Experiments and data analysis were repeated twice, respectively.

### Differential protein expression in KG1a before and after co-culture with HS5

**Fig. 2:**
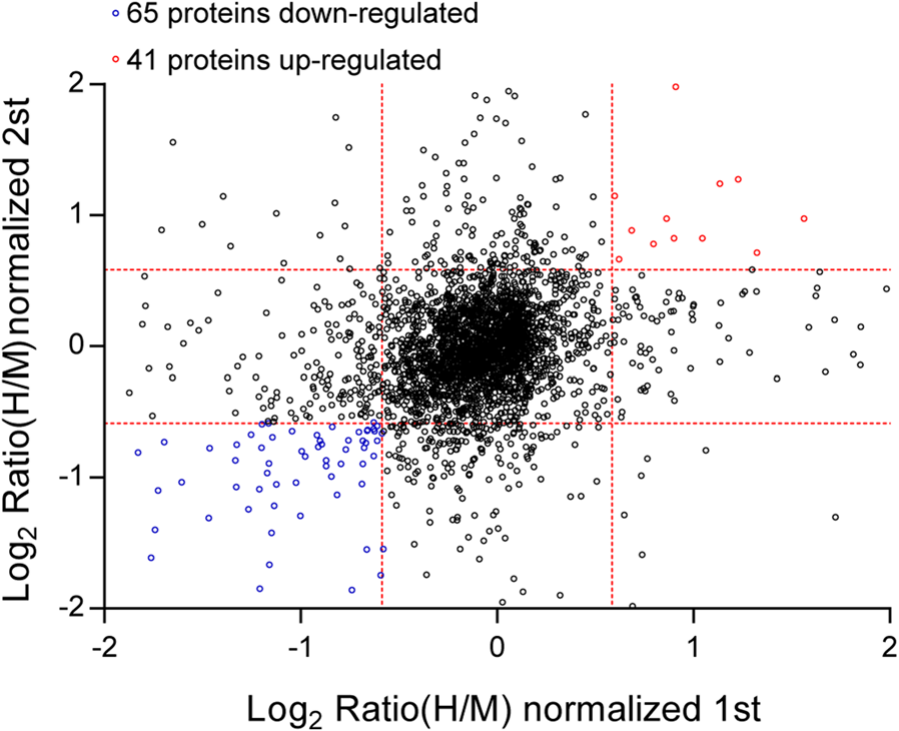
Normalized protein expression (scatter plot) of KG1a before and after co-culture with HS5. Proteins were quantified and normalized from two independent biological replicates. Proteins with ratios > 1.5 and Z-score ≥ 1.960σ from both experiments were considered to be upregulated. Proteins with ratios < 0.67 and Z-score ≥ 1.960σ from both experiments were considered to be downregulated

Overall, 5526 proteins were identified in both KG1a cultured alone and co-cultured with HS5. Of these, 41 proteins were significantly upregulated and 65 were significantly downregulated in co-cultured KG1a (|log2 ratio| of > 0.58 and z-score ≥ 1.960σ in two replicates) (Fig. 2; Additional file 1: Table S1). The top 10 upregulated proteins were plasminogen activator inhibitor 2 (SERPINB2), prelamin-A/C (LMNA), cytoskeleton-associated protein 4 (CKAP4), annexin A2 (ANXA2), neuroblast differentiation-associated protein (AHNAK), tissue factor pathway inhibitor 2 (TFPI2), tropomyosin alpha-1 chain (TPM1), protein S100-A6 (S100A6), myosin-10 (MYH10), and ubiquitin carboxyl-terminal hydrolase isozyme L1 (UCHL1) (Additional file 1: Table S2, Additional file 1: Fig. S1). SerpinB2, a member of the clade B or ovalbumin-like serine protease inhibitor (ov-serpin) subgroup of the serpin superfamily, is highly regulated in a cell type specific manner analogous to that of cytokines and oncogenes [27]. SerpinB2 is expressed by a range of cells including cancer cells, monocyte/macrophages, fibroblasts, endothelial cells, and dendritic cells and is often upregulated during inflammatory conditions [28]. CKAP4 (cytoskeleton-associated protein 4) was identified as Dickkopf1 (DKK1) receptor, which is correlated with prognosis in pancreatic, lung, and esophageal cancers, and the DKK1 signaling pathway was always activated during cancer cell proliferation [29–31]. The top 10 downregulated proteins were CD109 antigen (CD109), tyrosine-protein kinase Lyn (LYN), PCNA-associated factor (KIAA0101), integral membrane protein 2A (ITM2A), metalloreductase STEAP3 (STEAP3), histone H3.1 (HIST1H3A), tetraspanin (CD81), phosphoprotein associated with glycosphingolipid-enriched microdomains 1 (PAG1), histone H4 (HIST1H4A), and fatty acid desaturase 2 (FADS2) (Additional file 1: Table S3, Additional file 1: Fig. S2). CD109 is a glycosylphosphatidylinositol (GPI) anchored glycoprotein that presents on the cytoplasmic membrane. The biological functions of CD109 is to attenuate TGF-β signaling as a component of the co-receptor complex for TGF-β ligands. It has been studied as a cell surface antigen of hematopoietic cells and also as a cancer-associated protein in hematopoietic malignancies and human solid tumors [32]. Differentially expressed proteins are summarized in Additional file 1: Table S1.

### Functional analysis of regulated proteins in co-cultured KG1a

**Fig. 3:**
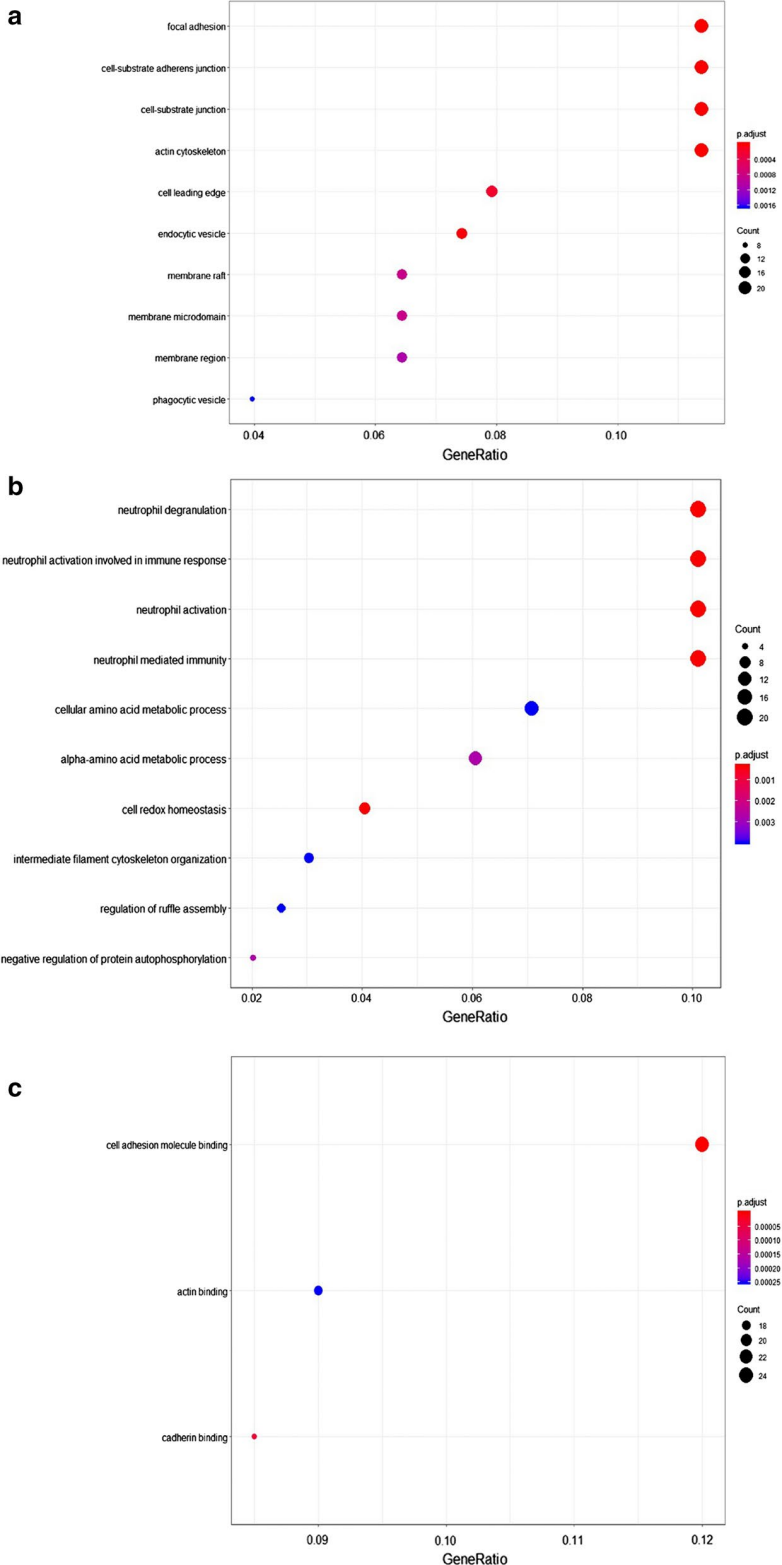

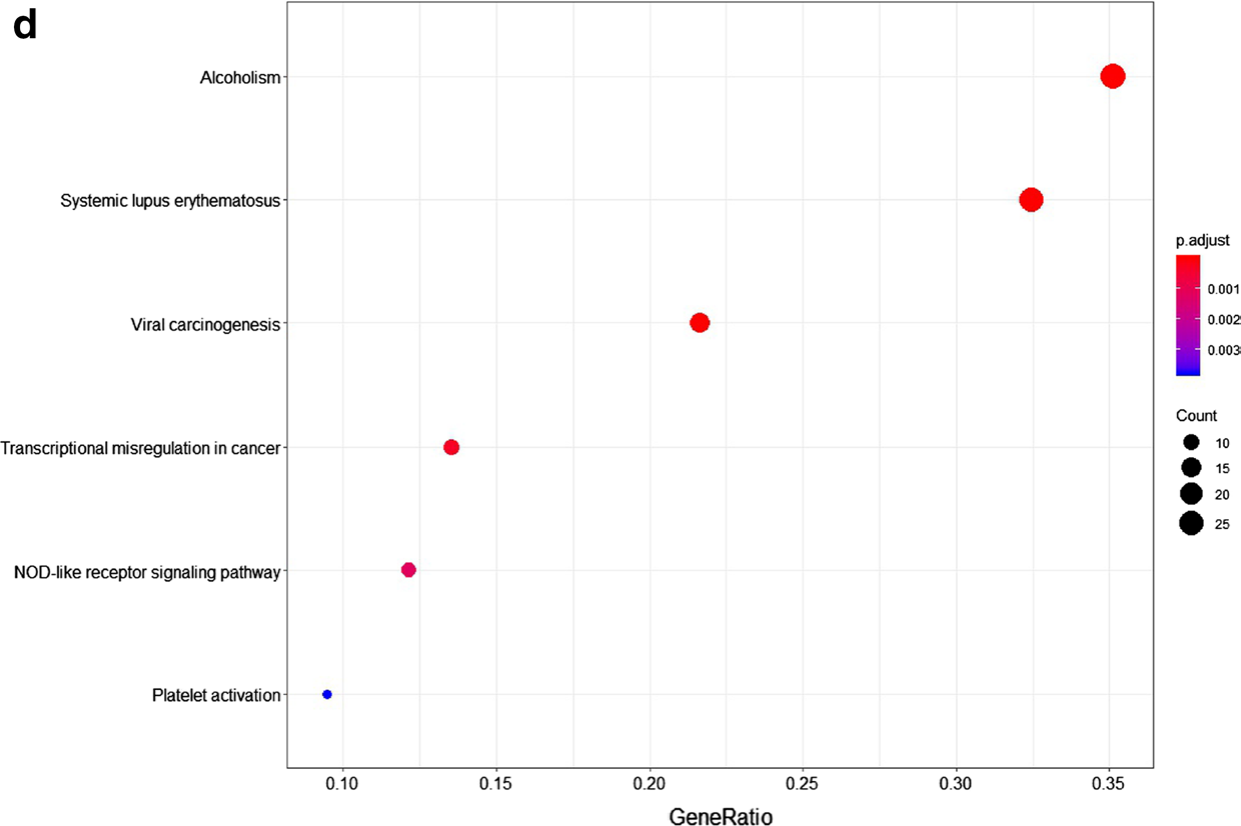
Functional classification of differentially expressed proteins of KG1a after co-culture with HS5, based on GO enrichment annotation terms. Proteins shown were linked to at least one annotation term within the GO cellular component, GO-CC (**a**), GO biological process, GO-BP (**b**), and GO molecular function, GO-MF (**c**). Over-represented pathways of significantly regulated proteins were retrieved from KEGG (FDR p-value < 0.05) (**d**)

Significantly regulated proteins in KG1a after co-culture with HS5 were analyzed by functional annotation, including GO enrichment annotation, metabolic and canonical pathway enrichment, and interconnecting networks. The major cellular component categories were nuclear chromatin, protein-DNA complex, and nuclear nucleosome et al. (Fig. 3a). The major biological process categories were protein heterotetramerization, protein heterooligomerization, and DNA replication-dependent nucleosome assembly et al. (Fig. 3b). The most common molecular function categories were cell adhesion molecule binding, cadherin binding, and nucleosomal DNA binding et al. (Fig. 3c).

Over-represented pathways of significantly regulated proteins (including both up-regulated and down-regulated proteins) were retrieved from KEGG (FDR p-value < 0.05) (Fig. 3d). Proteins involved in the NOD-like receptor signaling pathway were upregulated, whereas proteins involved in platelet activation were downregulated (Table 1).

**Table 1:** Regulated proteins of KG1a after co-culture with HS5 in over-represented KEGG pathways: NOD-like receptor signaling pathway and platelet activation

KEGG pathway	FDR p-value	Protein accessions	Gene names	Protein descriptions	Average ratio H/M
NOD-like receptor signalling pathway	1.67E−4	P07858	CTSB	Cathepsin B	3.66
		Q5XLA6	CARD17	Caspase recruitment domain-containing protein 17	2.91
		P21796	VDAC1	Voltage-dependent anion-selective channel protein 1	2.51
		P45880	VDAC2	Voltage-dependent anion-selective channel protein 2	2.46
		Q96PP8	GBP5	Guanylate-binding protein 5	2.38
		P43490	NAMPT	Nicotinamide phosphoribosyltransferase	1.89
		Q96PP9	GBP4	Guanylate-binding protein 4	1.73
		P32455	GBP1	Guanylate-binding protein 1	1.60
Platelet activation	1.5E−3	P43405	SYK	Tyrosine-protein kinase SYK	0.61
		Q01970	PLCB3	1-Phosphatidylinositol 4,5-bisphosphate phosphodiesterase beta-3	0.58
		Q9BV40	VAMP8	Vesicle-associated membrane protein 8	0.56
		Q7LDG7	RASGRP2	Calcium and DAG-regulated guanine nucleotide exchange factor I	0.56
		P08754	GNAI3	Guanine nucleotide-binding protein G(k) subunit alpha	0.54
		O00161	SNAP23	Synaptosomal-associated protein 23	0.49
		P07948	LYN	Tyrosine-protein kinase Lyn	0.30

Columns list protein UniProt accession numbers, gene names, protein descriptions, and SILAC average ratios from two biological replicates

**Fig. 4:**
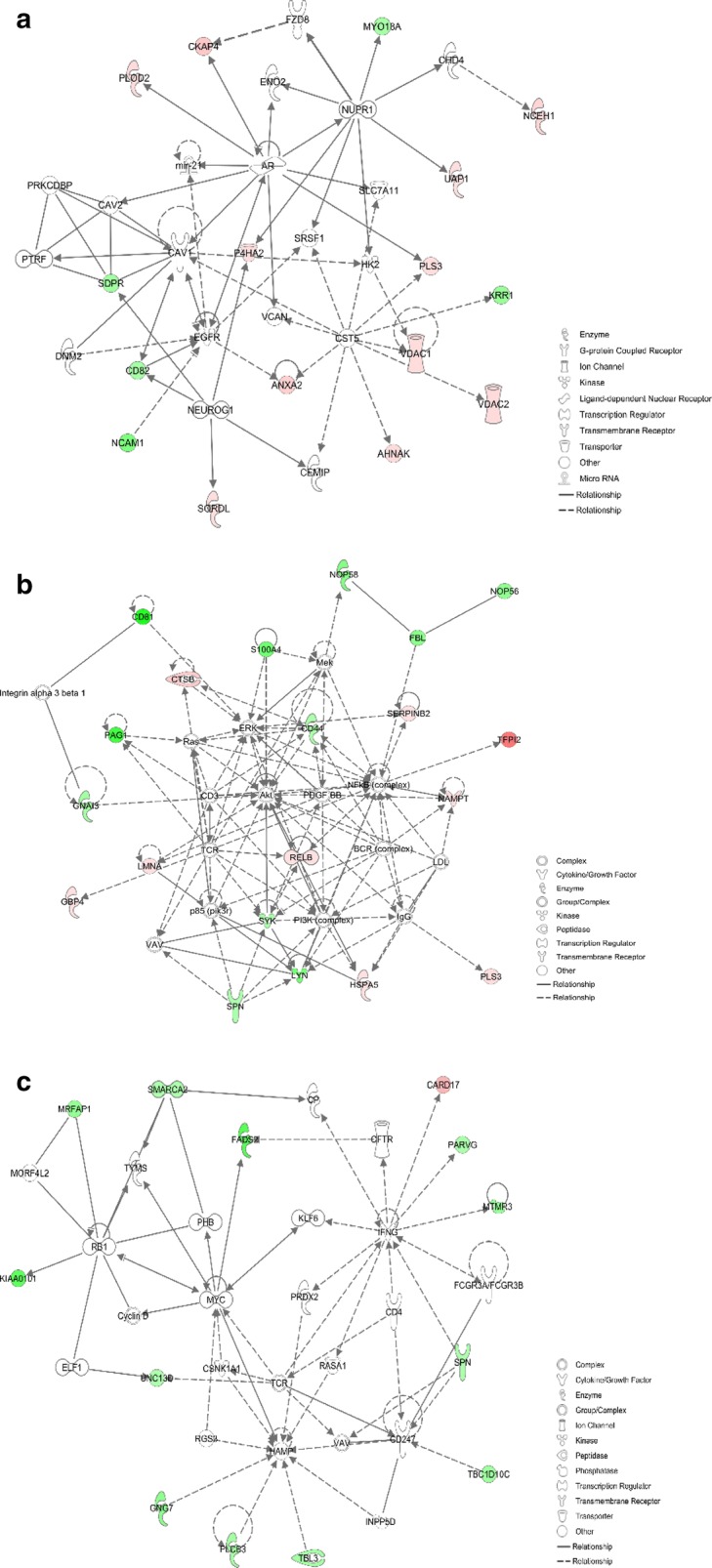
Functional network, from IPA analysis, of differentially regulated proteins identified in KG1a co-cultured with HS5. Top network functions of cancer, cellular movement, hereditary disorder (**a**), cell death and survival, cellular movement, cell signaling (**b**), and cellular movement, cancer, organismal injury and abnormalities (**c**). Red icons: upregulated proteins in co-cultured KG1a. Green icons: downregulated proteins in co-cultured KG1a. Color intensity indicates degree of variation

The top network functions identified as differentially expressed proteins in co-cultured KG1a were cancer, cellular movement, hereditary disorder (Fig. 4a), cell death and survival, cellular movement, cell signaling (Fig. 4b), cell morphology, cardiovascular disease, and hematological disease (Fig. 4c).

### Differential protein expression in HS5 before and after co-culture with KG1a

**Fig. 5:**
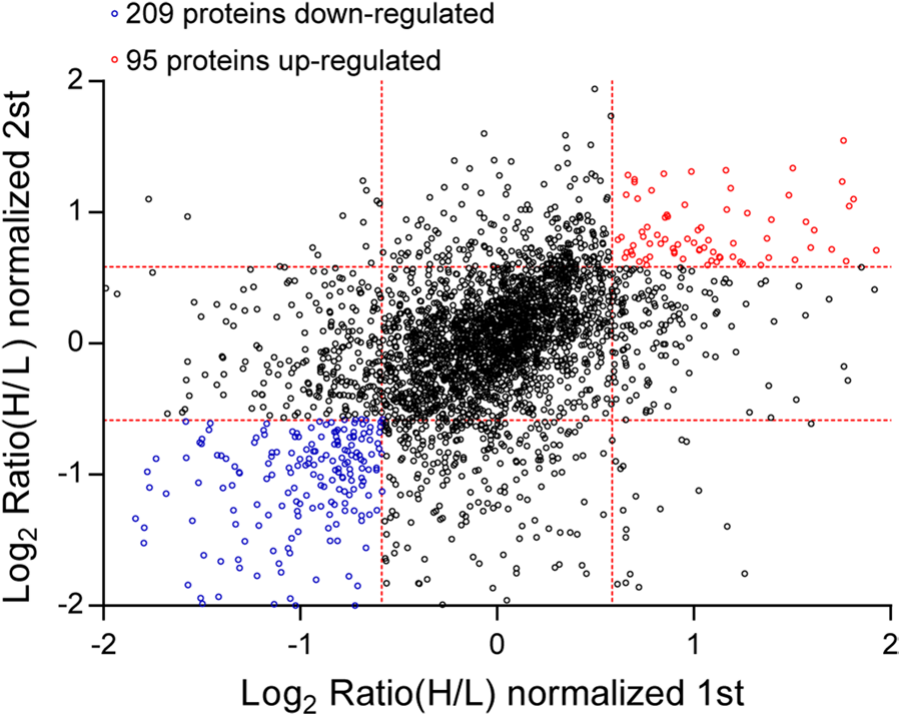
Normalized protein expression (scatter plot) of HS5 before and after co-culture with KG1a. Proteins were quantified and normalized from two independent biological replicates. Proteins with ratios > 1.5 and Z-score ≥ 1.960σ from both experiments were considered to be upregulated. Proteins with ratios < 0.67 and Z-score ≥ 1.960σ from both experiments were considered to be downregulated

HS5 in the presence and absence of KG1a were analyzed by quantitative proteomics (SILAC method). A total of 5922 proteins were identified in both HS5 cultured alone and co-cultured with KG1a. 95 proteins were significantly upregulated and 209 were significantly downregulated in co-cultured HS5 (|log2 ratio| of > 0.58 and z-score ≥ 1.960σ in two replicates) (Fig. 5; Additional file 1: Table S4). The top 10 upregulated proteins were fermitin family homolog 3 (FERMT3), Ena/VASP-like protein (EVL), neural cell adhesion molecule 1 (NCAM1), plastin-2 (LCP1), Rho GDP-dissociation inhibitor 2 (ARHGDIB), coronin-1A (CORO1A), glia maturation factor gamma (GMFG), integrin alpha-L (ITGAL), cyclin-dependent kinase 6 (CDK6), and lymphocyte-specific protein 1 (LSP1) (Additional file 1: Table S5, Additional file 1: Fig. S3). The neural cell adhesion molecule 1 (NCAM1) is a fundamental protein in cell–cell interaction and in cellular developmental processes including cell migration and survival, axon guidance, and synaptic targeting, and its dysregulation is involved in a number of diseases [33]. Lymphocyte cytosolic protein 1 (LCP1), a lymphocyte-specific target, plays a critical role in B-cell biology by crosslinking F-actin filaments, thereby solidifying cytoskeletal structures and providing a scaffold for critical signaling pathways. It was reported LCP1 was highly expressed in leukemia and lymphoma cell lines [34]. The top 10 downregulated proteins were dermcidin (DCD), tissue-type plasminogen activator (PLAT), Target of Nesh-SH3 (ABI3BP), serpin B7 (SERPINB7), alpha-internexin (INA), neuronal-specific septin-3 (SEPT3), cysteine-rich protein 2 (CRIP2), sulfhydryl oxidase 1 (QSOX1), neprilysin (MME), and CCAAT/enhancer-binding protein beta (CEBPB) (Additional file 1: Table S6, Additional file 1: Fig. S4). Abi3 bp has been reported to play an important role in MSC biology in many MSC processes, including proliferation, differentiation, adhesion, morphology and transformation [35]. Differentially expressed proteins are summarized in Additional file 1: Table S4.

### Functional analysis of regulated proteins in co-cultured HS5

**Fig. 6:**
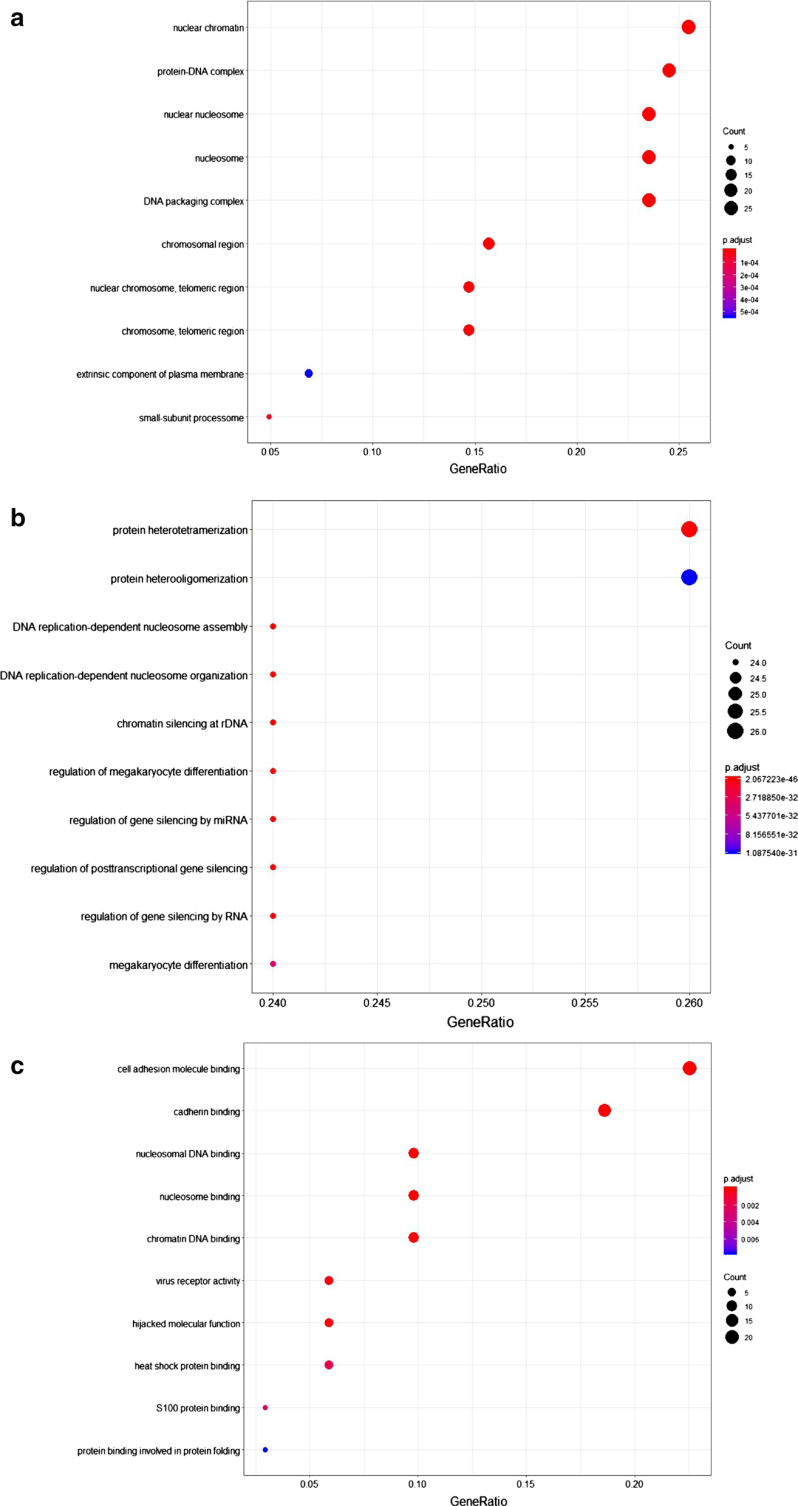

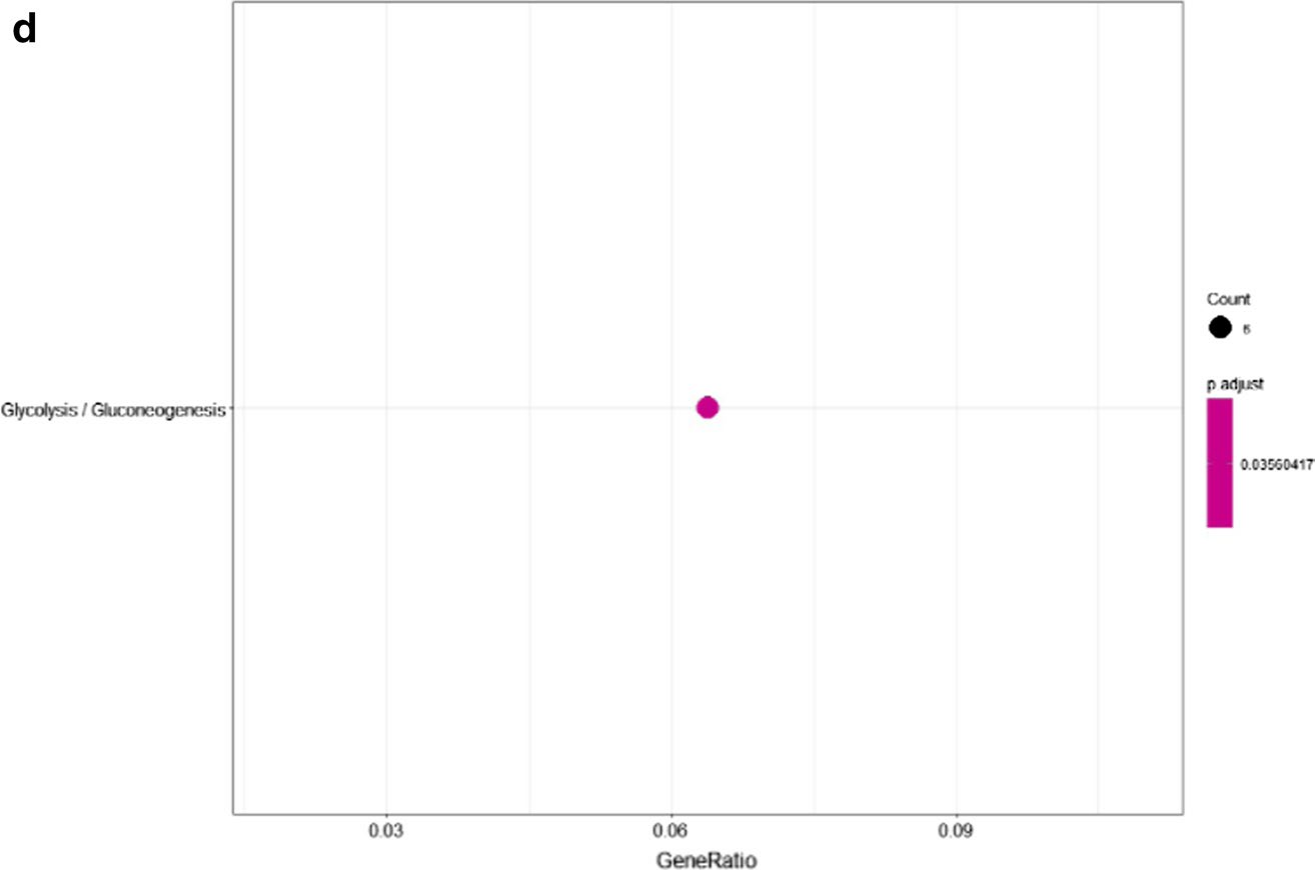
Functional classification of differentially expressed proteins of HS5 after co-culture with KG1a, based on GO enrichment annotation terms. Proteins shown were linked to at least one annotation term within the GO cellular component, GO-CC (**a**), GO biological process, GO-BP (**b**), and GO molecular function, GO-MF (**c**). Over-represented pathways of significantly regulated proteins were retrieved from KEGG (FDR p-value < 0.05) (**d**)

Significantly regulated proteins in HS5 after co-culture with KG1a were analyzed by functional annotation, including GO enrichment annotation, metabolic and canonical pathway enrichment, and interconnecting networks. The major cellular component categories were focal adhesion, cell-substrate adherens junction, cell-substrate junction, actin cytoskeleton and et al. (Fig. 6a). The major biological process categories were neutrophil degranulation, neutrophil activation involved in immune response, neutrophil activation, neutrophil mediated immunity and et al. (Fig. 6b). The most common molecular function categories were cell adhesion molecule binging, actin binding and cadherin binding (Fig. 6c).

Over-represented pathways of significantly regulated proteins (including both up-regulated and down-regulated proteins) were retrieved from KEGG (FDR p-value < 0.05) (Fig. 6d, Table 2).

**Table 2:** Regulated proteins of HS5 after co-culture with KG1a in over-represented KEGG pathways: tight junction and glycolysis/gluconeogenesis

KEGG pathway	FDR p-value	Protein accessions	Gene names	Protein descriptions	Average ratio H/L
Glycolysis/gluconeogenesis	3.95E−2	P08237	PFKM	ATP-dependent 6-phosphofructokinase, muscle type	0.62
		P52789	HK2	Hexokinase-2; hexokinase	2.37
		P14550	AKR1A1	Alcohol dehydrogenase [NADP(+)]	2.83
		P09104	ENO2	Gamma-enolase; enolase	0.46
		Q16822	PCK2	Phosphoenolpyruvate carboxykinase [GTP], mitochondrial	1.74
		P47895	ALDH1A3	Aldehyde dehydrogenase family 1 member A3	0.36

Columns as in Table 1

**Fig. 7:**
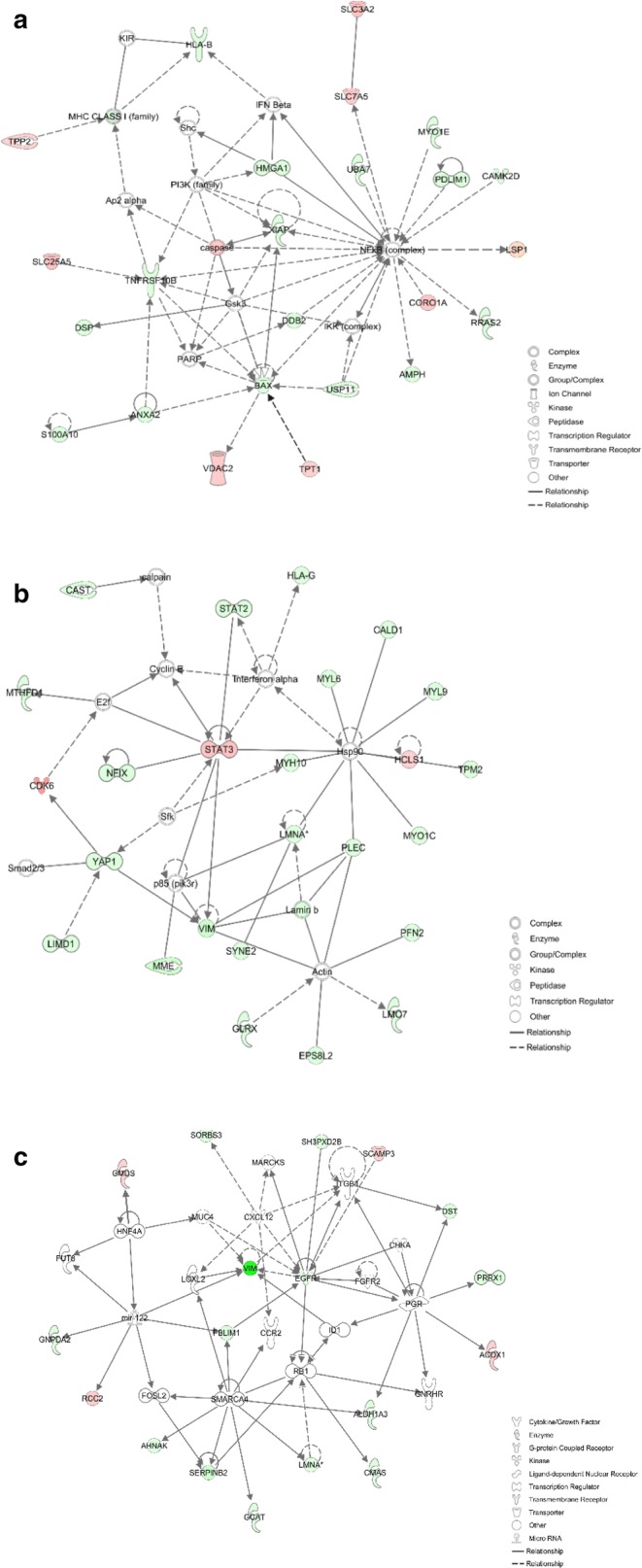
Functional network, from IPA analysis, of differentially regulated proteins identified in HS5 co-cultured with KG1a. Top network functions of cell death and survival, amino acid metabolism, molecular transport (**a**), cell morphology, cellular movement and developmental disorder (**b**), and cellular movement, cancer, organismal injury and abnormalities (**c**). Red icons: upregulated proteins in co-cultured HS5. Green icons: downregulated proteins in co-cultured HS5. Color intensity indicates degree of variation

The top network functions identified as differentially expressed proteins in co-cultured HS5 were death and survival, amino acid metabolism, molecular transport (Fig. 7a), cell morphology, cellular movement, and developmental disorder (Fig. 7b), and cellular movement, cancer, organismal injury, and abnormalities (Fig. 7c).

### Confirmation of differential protein expression by western blotting

**Fig. 8:**
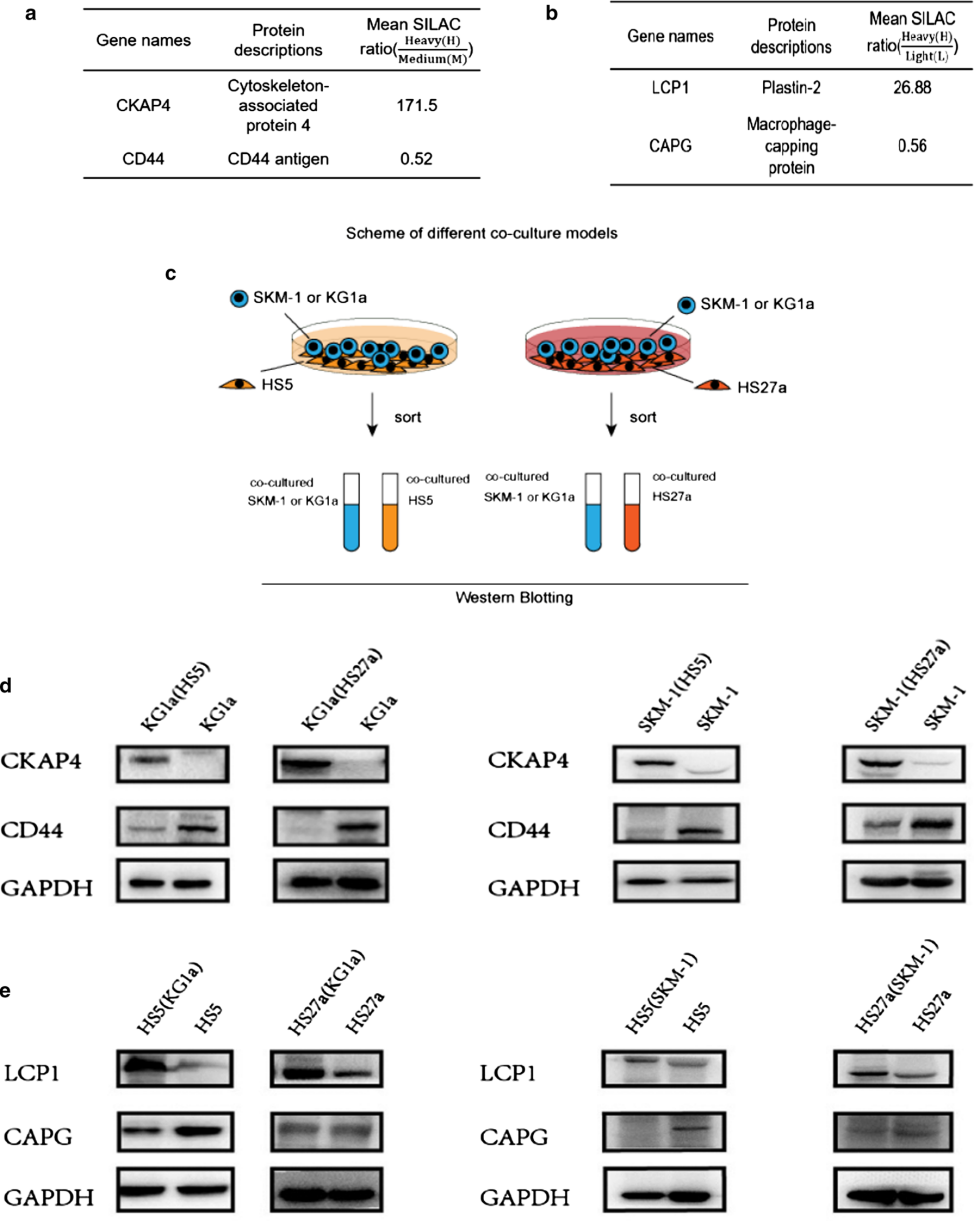

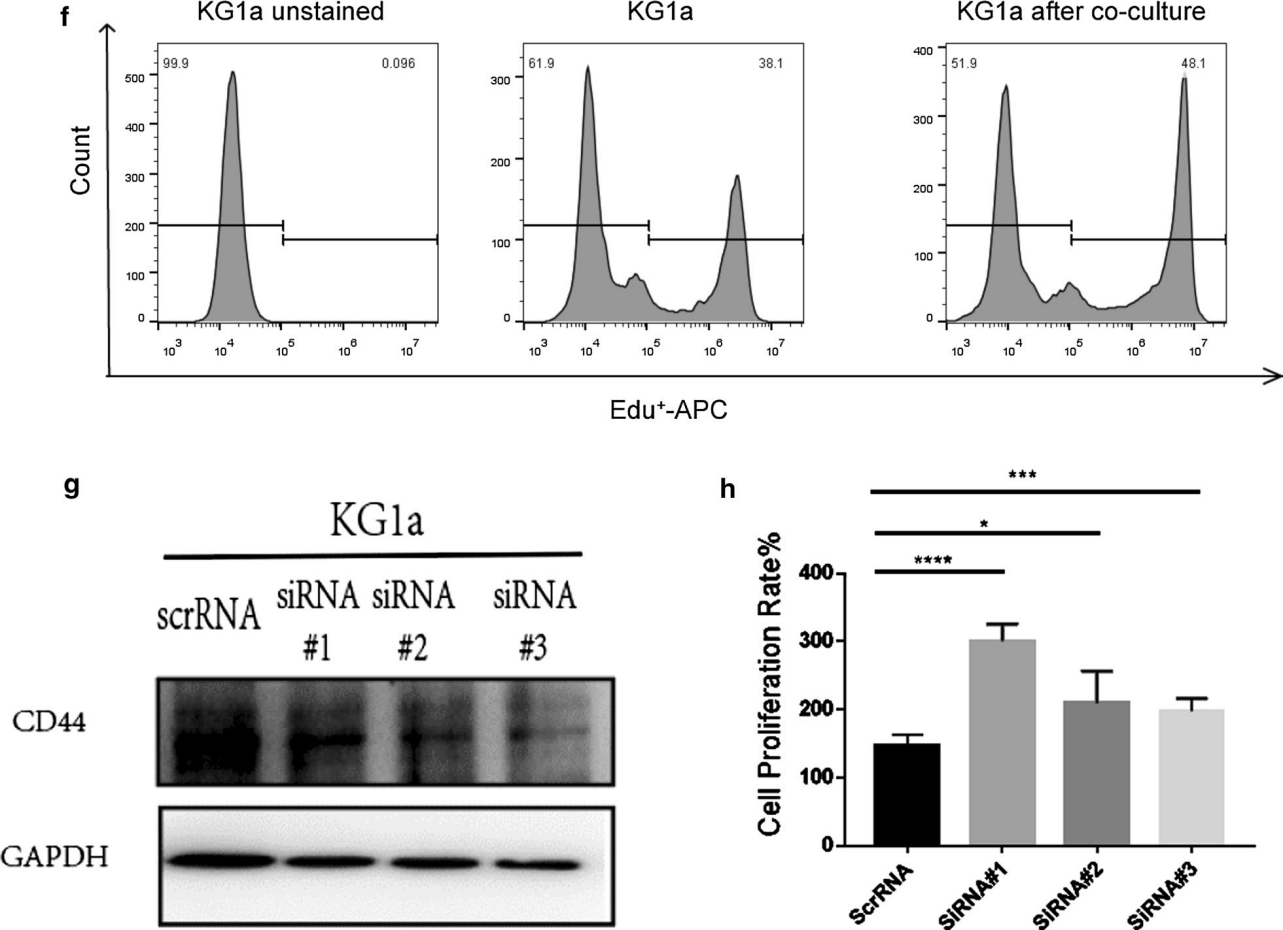
Western blotting of differentially expressed proteins. Selected proteins of CKAP4, CD44, LCP1 and CAPG from SILAC dataset. **a**, **b** The scheme of different co-culture model (**c**). Protein expression of CKAP4, CD44, LCP1 and CAPG to confirm SILAC results in different co-cultured models. GAPDH served as loading control. **d**, **e** “KG1a(HS5)”: KG1a after co-culture with HS5, “KG1a(HS27a)”: KG1a after co-culture with HS27a, “SKM-1(HS27a)”: SKM-1 after co-culture with HS27a, “SKM-1(HS5)”: SKM-1 after co-culture with HS5,”HS5(KG1a)”: HS5 after co-culture with KG1a. “HS27a(KG1a)”: HS27a after co-culture with KG1a, “HS27a(SKM-1)”: HS27a after co-culture with SKM-1, “HS5(SKM-1)”: HS5 after co-culture with SKM-1. **f** Flow cytometry analysis confirmed the proliferation of KG1a after co-cultured with HS27a. **g** Western blotting to verify the efficiency of knockdown CD44 in KG1a. **h** Detects Cell proliferation was detected by Cell Counting Kit-8 (CCK-8 kit). ScrRNA, KG1a is interfered by Scramble RNA. SiRNA#1, siRNA#2, siRNA#3 are three different specific siRNAs used to knock down CD44

Subsequently, we selectively validated the diffentially expressed proteins from proteomics data. We picked four proteins, CKAP4, CD44, LCP1 and CAPG, from the SILAC dataset. Proteomic data showed CKAP4 expression was elevated (SILAC ratios = 171.5) whereas CD44 was reduced(SILAC ratios = 0.52) in co-cultured KG1a (Fig. 8a) while LCP1 expression was strongly elevated((SILAC ratios = 26.88) but CAPG was reduced (SILAC ratios = 0.56) in co-cultured HS5 (Fig. 8b). We performed western blotting to confirm these proteins expression in co-cultured KG1a and HS5. The results were consistent with SILAC data (Fig. 8d, e). In order to test these proteins can also be found in different co-culture models, we validated them using bone marrow derived stromal cells HS27a co-culture with either KG1a or with SKM-1 and HS5 co-cultured with SKM-1(Fig. 8c). The results showed that CKAP4 was elevated whereas CD44 was reduced in either co-cultured KG1a or co-cultured SKM-1 after contacted with different stromal cells (Fig. 8d). LCP1 expression was strongly elevated whereas CAPG expression was deceased in co-cultured HS5 or co-cultured HS27a cells after co-cultured with KG1a or SKM-1 (Fig. 8e). However, there were no significant changes in the co-cultured normal hematopoietic cells and stromal cells (Additional file 1: Fig. S5). Altogether, the quantitative proteomics by SILAC method can be utilized in different types of co-culture studies and to verify the variety of signals exchanged between hematopoietic cells and their niche. We then investigated the functional role of CD44 in myeloid clonal cells. First, we found cell proliferation of KG1a was increased after co-cultured with HS27a (Fig. 8f). Our proteomic data and western blotting showed CD44 was decreased in co-cultured KG1a. Next, we carried out siRNA assay to knock down CD44 in KG1a alone cells to investigate cell proliferation. We found cell proliferation of KG1a was enhanced after CD44 was knocked down (Fig. 8g, h), suggesting CD44 correlated with cell proliferation in myeloid clonal cells.

## Discussion

Tumor cells can acquire “stem” characteristics through interaction with and manipulation of their microenvironment. Typically, tumor cell/stromal cell interactions are part of the cellular social network, play an essential role in tumor development, and are potentially a useful therapeutic target [36]. Hematopoietic stem cells are regulated by biochemical and physical contextual signals arising from their microenvironment, which includes osteoblasts, mesenchymal/stromal cells, endothelial cells, pericytes, macrophages, matrix structures, and soluble factors. In particular, the subendosteal and vascular niches are two distinct microenvironmental structures that can alter hematopoiesis. Raaijmakers et al. [37] showed that deletion of Dicer 1 in murine osteoblast progenitors resulted in development of dysplastic murine hematopoiesis. Marrow stromal cells, in the presence of clonal MDS cells, displayed abnormal gene expression and function [5, 38]. Our previous studies of an in vitro co-culture system, using stromal cell line HS5 derived from a healthy marrow donor, showed that advanced MDS displayed TNF-α-induced sensitivity to apoptosis following stromal contact [13, 39]. Based on our in vitro observations, we intravenously co-injected HS5 cells and MDS marrow-derived hematopoietic cells into Nod.cg-Prkdc^*scid*^ Il2rg^*tm1wjll*^ (NSG) mice, and observed that differential stromal contact had structural and functional effects on proliferation of MDS precursor cells [40]. Also, we used glycomics (lectin microarray) and proteomics (SILAC method) approaches to compare two bone marrow stromal cell lines, HS5 and HS27a, and observed differential expression of several glycans and proteins [18, 41]. These and many similar findings indicate that microenvironmental factors play key roles in both normal and pathological hematopoiesis, and that hematopoiesis is modulated by bidirectional signals between hematopoietic cells and the microenvironment. Based on these findings, we further labeled proteins in HS5 and KG1a cells with light, medium, and heavy stable isotopes of Arg and Lys, to evaluate the proteomes of the two cell types before and after their physical contact.

### The influence for hematopoietic cells before and after co-culture with stroma

To investigate the heterogeneity of bone marrow stromal cells, the proteome of HS5 and HS27a were previously analyzed using SILAC method [18]. We found 28 proteins differentially expressed in both proteomic and genomic database of HS5 and HS27a. After modified one of the differentially expressed proteins, IGTV, in HS27a, we could influence the apoptosis of co-cultured hematopoietic cells, suggesting the important role of stromal cells in determining the cell fate of co-cultured hematopoietic cells [18]. In this study, we found CKAP4, LMNA, and SERPINB2 were upregulated, whereas CD44, CD99, and NCAM1 were downregulated in KG1a co-cultured with HS5. Western blotting confirmed the increase of CKAP4 and decrease of CD44 in these cells. We also demonstrated the expression of CKAP4 was up-regulated and CD44 was down-regulated in co-cultured SKM-1 after contacted with either HS27a or HS5 and in co-cultured KG1a after contacted with HS27a. CD44 is a transmembrane glycoprotein expressed on the surface of normal and leukemic cells [42]. Our results demonstrated cell proliferation was increased but the expression of CD44 was down-regulated in co-cultured KG1a. Thus we knocked down CD44 in KG1a and found cell proliferation was enhanced thereafter. Our data consist with the previous findings that knock-out of CD44 could enhance cell proliferation in endothelial cells [43]. These data suggested CD44 expression correlates with cell proliferation in myeloid clonal cells and its related molecular mechanism needs to be further developed. The other potential-targeted proteins identified by this SILAC labeled co-culture setting can be further investigated their influence of stromal cells in microenvironment on the hematopoietic cells.

### The influence for stroma cells before and after co-culture with hematopoietic cells

Alterations of MDS stroma are dependent on the presence of clonal MDS cells, and are reversible by elimination of clonal cells. Our findings indicate cross-interaction and cross-manipulation between hematopoietic cells and their microenvironment; i.e., the interactions are mutual. Signals in the opposite direction (from clonal hematopoietic cells to stroma) remain to be characterized. In an analysis of transcriptomic profiles of mesenchymal stromal cells from healthy donors and myeloma patients before and after co-culture with myeloma cell line MM.1S, Garcia-Gomez et al. [44] found that CXCL1, CXCL5, and CXCL6 were activated by the myeloma cells. In the present study, HS5 co-cultured with KG1a showed upregulation of LCP1, ARHGAP4, and UNCX, and downregulation of CAPG, FLNC, and MAP4. LCP1 (lymphocyte cytosolic protein 1), also known as plastin-2, is expressed highly in chronic lymphocytic leukemia (CLL) and was found to crosslink F-actin filaments, thereby helping to strengthen cytoskeletal structures and to build a scaffold for essential signaling pathways [34]. CXC chemokine receptor 4 (CXCR4), which is a prognostic marker for gastric cancer, leukemia, and other cancers, and can increase levels of its downstream molecule LCP1, is activated by the regulator GLI1 [45]. Further studies are needed to clarify the role of LCP1 in the bone marrow niche involved in CXCR4/CXCR7 signaling axis. CAPG (macrophage-capping protein) can modulate cell motility by interacting with the cytoskeleton [46, 47]. In CAPG-overexpressing pancreatic cancer cells, phosphorylation status of other actin-modulating proteins (cofilin, ezrin/radixin) was not significantly altered, suggesting possible involvement of other cellular proteins, such as ornithine aminotransferase and enolase [48]. CAPG may thus participate in the bone marrow niche via these enzymes and their downstream signaling pathway. Using different co-culture models, the western blotting results confirmed the increase of LCP1 and decrease of CAPG in co-cultured HS5 and in co-cultured HS27a after KG1a or SKM-1 incubation, which are in agreement with the proteomics data. The above findings, taken together, indicate that various proteins in stromal cells may display dysfunctional behavior in response to signals from a particular type of hematopoietic cells.

In this current work, we studied the whole proteomic profiles of hematopoietic cells and stromal cells before and after their interaction. The quantitative proteomic technique described here provides a useful basis for future studies involving co-culture models. Our note clearly shows that quantitative proteomics by SILAC method performed here can serve as a basic method for monitoring the differentially expressed proteins in co-culture models. Importantly, a wide range of analysis to study the crosstalk between stromal cells and hemaptopoietic cells can be added, including the intracellular signaling pathways. The proteomic profiles from the KG1a/stromal cell co-culture system give new molecular insights into the roles of these cells in MDS pathophysiology and related bone disease. For example, one study is in progress to investigate the functions and clinical relevance of differentially expressed proteins in this SILAC labeled co-culture setting.

## Conclusion

Altogether, we recommend such quantitative proteomics approach can be broadly used for the studies of the hematopoietic–stroma cross-talk, differentially expressed proteins and related signaling pathways identification. The differentially expressed proteins identified from this current SILAC method will provide a useful basis for ongoing studies of crosstalk between stromal cells and hematopoietic cells in co-culture systems. All these result suggested our ongoing studies can focus on the mechanisms underlying CKAP4 increase and CD44 decrease in co-cultured hematopoietic cells, and the increase of LCP1 and decrease of CAPG in co-cultured stromal cell. The proteomic profiles from the KG1a/stromal cell co-culture system give new molecular insights into the roles of these cells in MDS pathophysiology and related bone disease.

## Supporting information

Detailed SILAC data for the co-culture model are available as supplementary information.

## Additional file

**Additional file 1.** Functional annotations of dysregulated proteins in either KG1a or HS5 after co-cultured. Some verification assays for CKAP4 and CD44 in CD34+ primary cells. And the lists of significant regulated proteins in either KG1a or HS5 after co-culture.

## Data Availability

Please contact author for data requests.

## Abbreviations

SILAC: stable isotope labeling by amino acids in cell culture method
HSC: hematopoietic stem cells
MDS: myelodysplastic syndrome
TNF-α: tumor necrosis factor alpha
GO: gene ontology
KEGG: Kyoto encyclopedia of genes and genomes
IPA: the Ingenuity Systems Application
FDR: false discovery rate
MS: mass spectrometry
LC: liquid chromatography
CKAP4: cytoskeleton-associated protein 4
LCP1: plastin-2
CD44: CD44 antigen
CAPG: macrophage-capping protein
SERPINB2: plasminogen activator inhibitor 2
LMNA: prelamin-A/C
CKAP4: cytoskeleton-associated protein 4
ANXA2: annexin A2
AHNAK: neuroblast differentiation-associated protein
TFPI2: tissue factor pathway inhibitor 2
TPM1: tropomyosin alpha-1 chain
S100A6: protein S100-A6
MYH10: myosin-10
UCHL1: ubiquitin carboxyl-terminal hydrolase isozyme L1
CD109: CD109 antigen
LYN: tyrosine-protein kinase Lyn
KIAA0101: PCNA-associated factor
ITM2A: integral membrane protein 2A
STEAP3: metalloreductase STEAP3
HIST1H3A: histone H3.1
CD81: tetraspanin
HIST1H4A: histone H4
PAG1: phosphoprotein associated with glycosphingolipid-enriched microdomains 1
FADS2: fatty acid desaturase 2
FERMT3: fermitin family homolog 3
EVL: Ena/VASP-like protein
NCAM1: neural cell adhesion molecule 1
LCP1: plastin-2
ARHGDIB: Rho GDP-dissociation inhibitor 2
CORO1A: coronin-1A
GMFG: glia maturation factor gamma
ITGAL: integrin alpha-L
CDK6: cyclin-dependent kinase 6
LSP1: lymphocyte-specific protein 1
DCD: dermcidin
PLAT: tissue-type plasminogen activator
ABI3BP: target of Nesh-SH3
SERPINB7: serpin B7
INA: alpha-internexin
SEPT3: neuronal-specific septin-3
CRIP2: cysteine-rich protein 2
QSOX1: sulfhydryl oxidase 1
MME: neprilysin
CEBPB: CCAAT/enhancer-binding protein beta
DKK1: Dickkopf1 receptor
CXCR4: CXC chemokine receptor 4
CAPG: macrophage-capping protein
