# Supplementary material for: Screening differentially expressed proteins from co-cultured hematopoietic cells and bone marrow-derived stromal cells by quantitative proteomics (SILAC) method
Source: Clin Proteomics. 2019 Jul 18;16:32. doi: 10.1186/s12014-019-9249-x (PMC6637644; doi:10.1186/s12014-019-9249-x)
Supplement: Supplementary file 1 — Additional file 1. Functional annotations of dysregulated proteins in either KG1a or HS5 after co-cultured. Some verification assays for CKAP4 and CD44 in CD34+ primary cells. And the lists of significant regulated proteins in either KG1a or HS5 after co-culture. [file 12014_2019_9249_MOESM1_ESM.docx]

**Figure S1.** Functional annotation of up-regulated expressed proteins of KG1a after co-culture with HS5, based on GO enrichment annotation terms. Proteins shown were linked to at least one annotation term within the GO cellular component, GO-CC (A), GO biological process, GO-BP (B), and GO molecular function, GO-MF (C). Over-represented pathways of significantly regulated proteins were retrieved from KEGG (FDR p-value <0.05). (D)


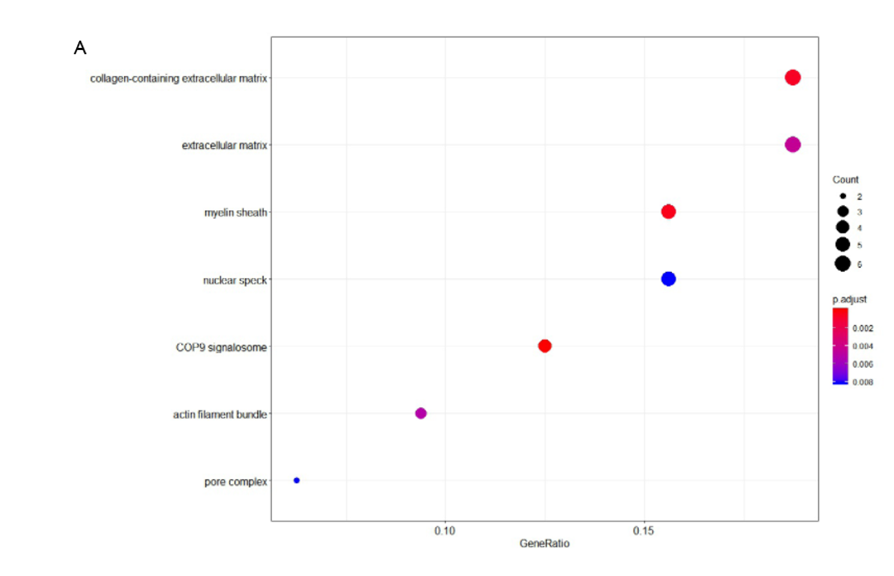


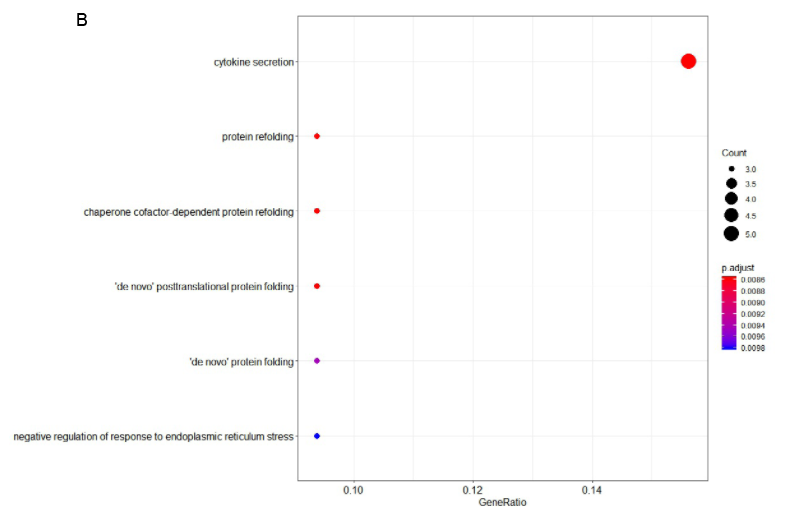


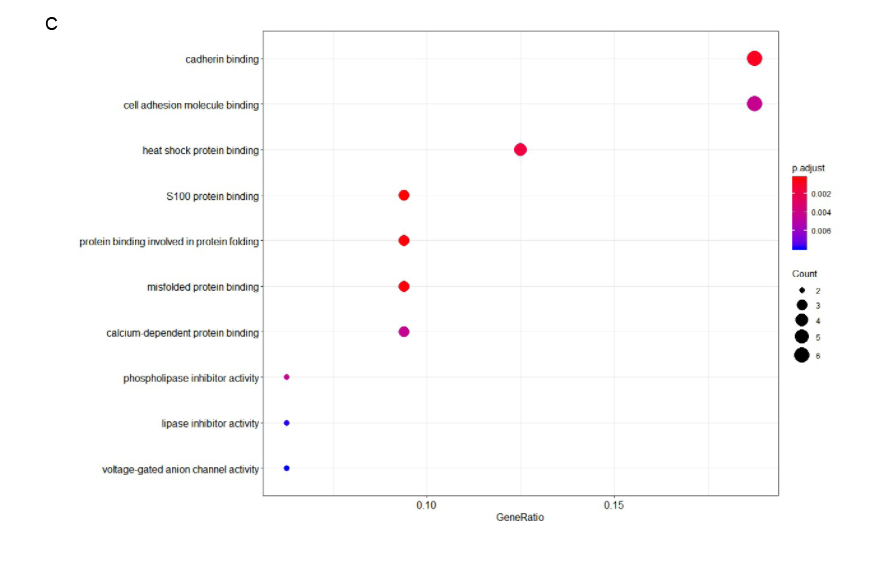


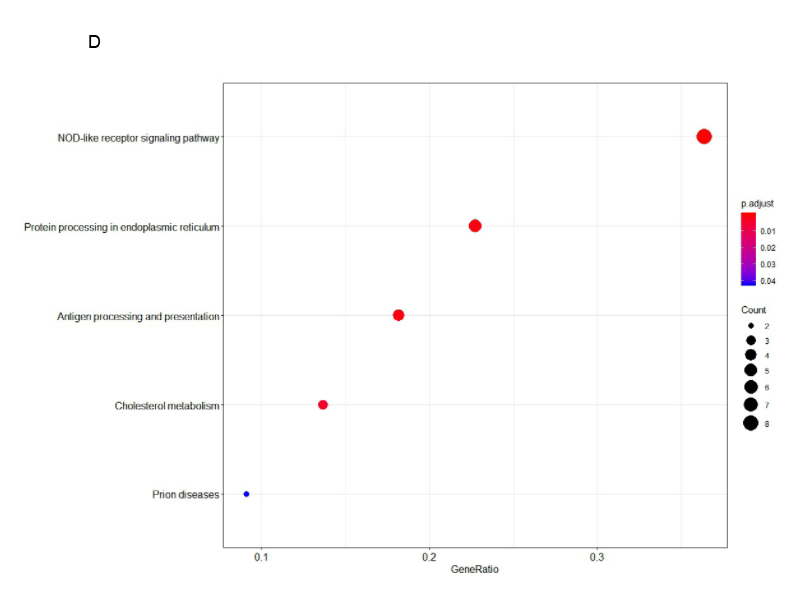


**Figure S2.** Functional annotation of down-regulated expressed proteins of KG1a after co-culture with HS5, based on GO enrichment annotation terms. Proteins shown were linked to at least one annotation term within the GO cellular component, GO-CC (A), GO biological process, GO-BP (B), and GO molecular function, GO-MF (C). Over-represented pathways of significantly regulated proteins were retrieved from KEGG (FDR p-value <0.05) (D).


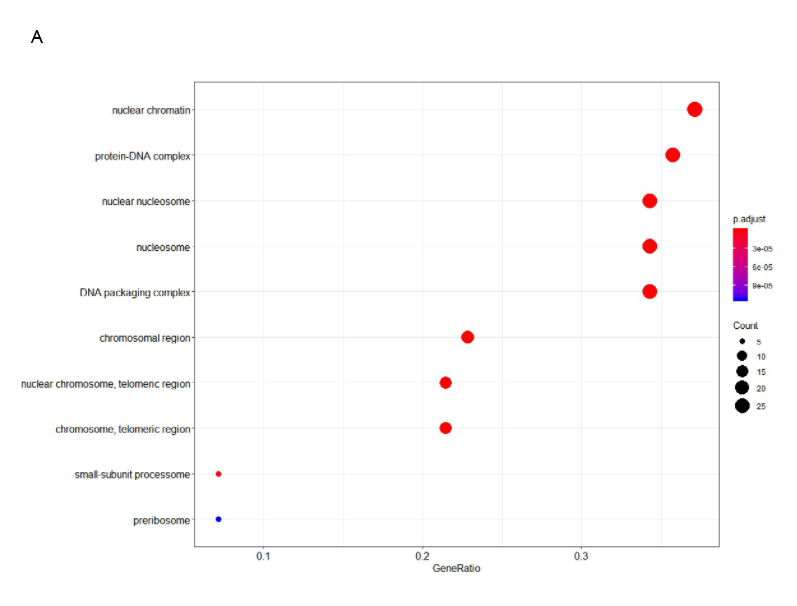


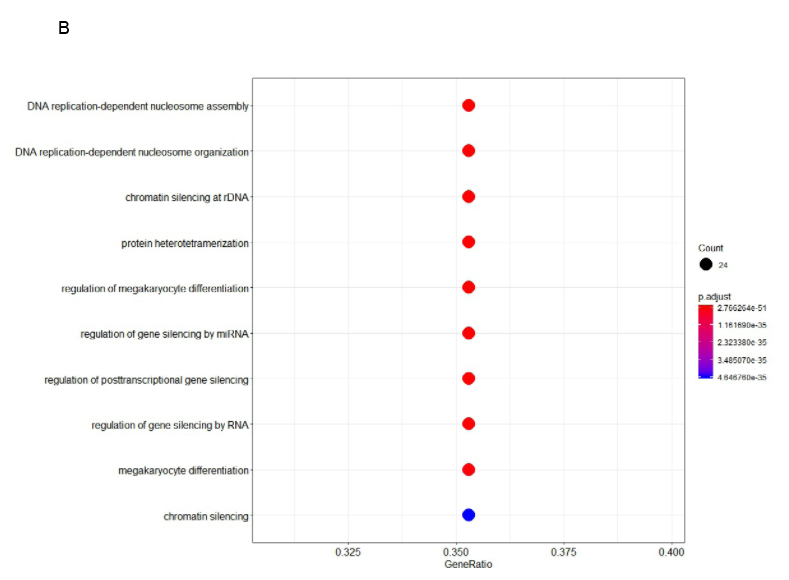


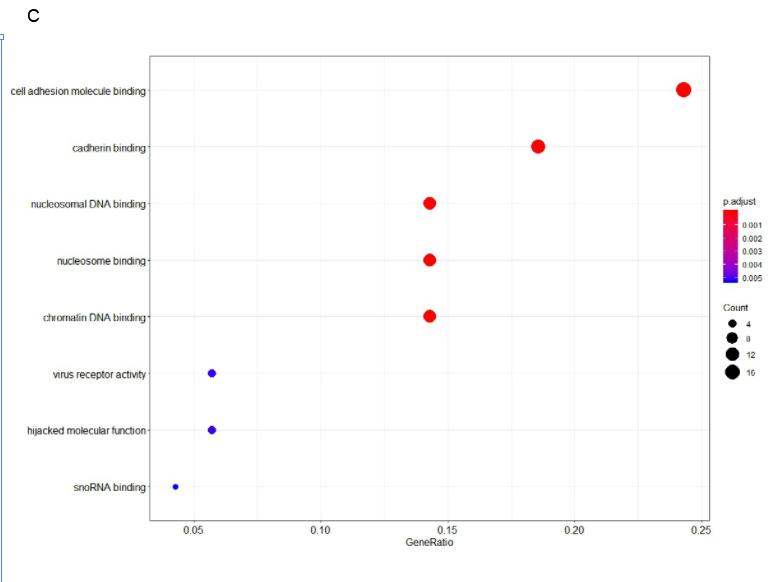


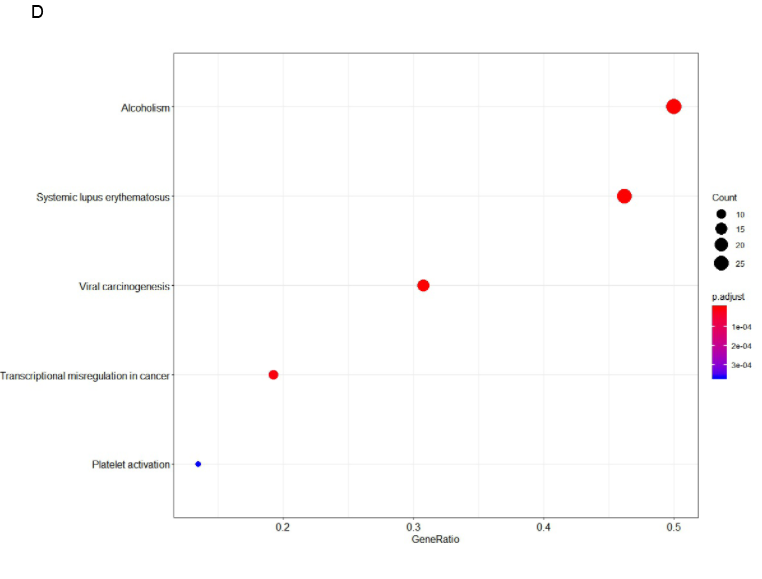


**Figure S3.** Functional annotation of up-regulated expressed proteins of HS5 after co-culture with KG1a, based on GO enrichment annotation terms. Proteins shown were linked to at least one annotation term within the GO cellular component, GO-CC (A), GO biological process, GO-BP (B).


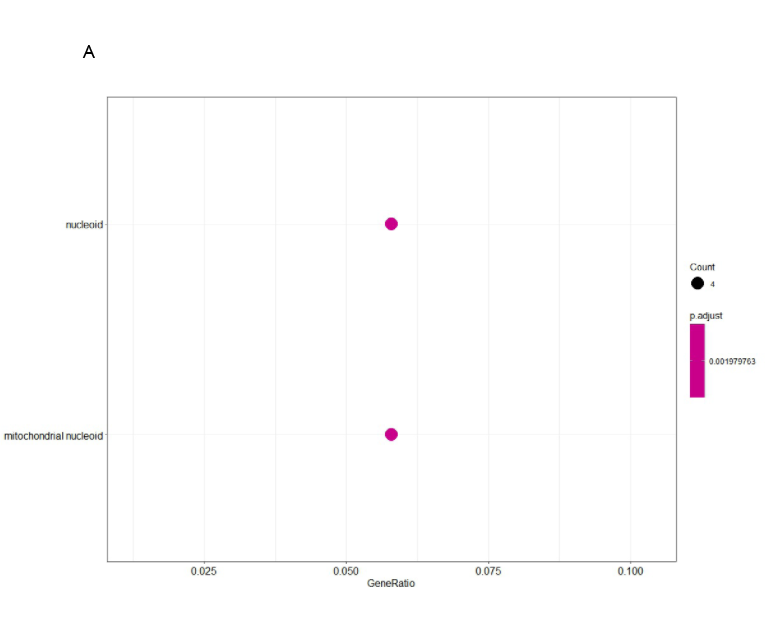


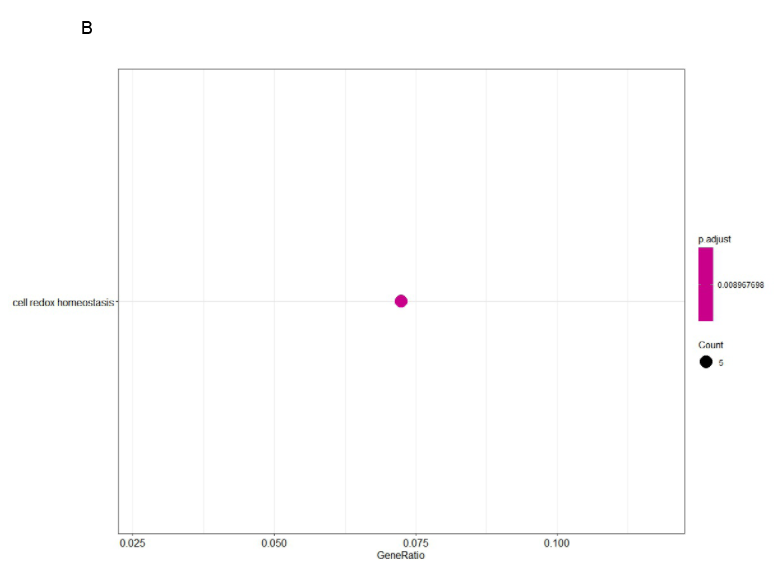


**Figure S4.** Functional annotation of down-regulated expressed proteins of HS5 after co-culture with KG1a, based on GO enrichment annotation terms. Proteins shown were linked to at least one annotation term within the GO cellular component, GO-CC (A), GO biological process, GO-BP (B), and GO molecular function, GO-MF (C). Over-represented pathways of significantly regulated proteins were retrieved from KEGG (FDR p-value <0.05) (D)


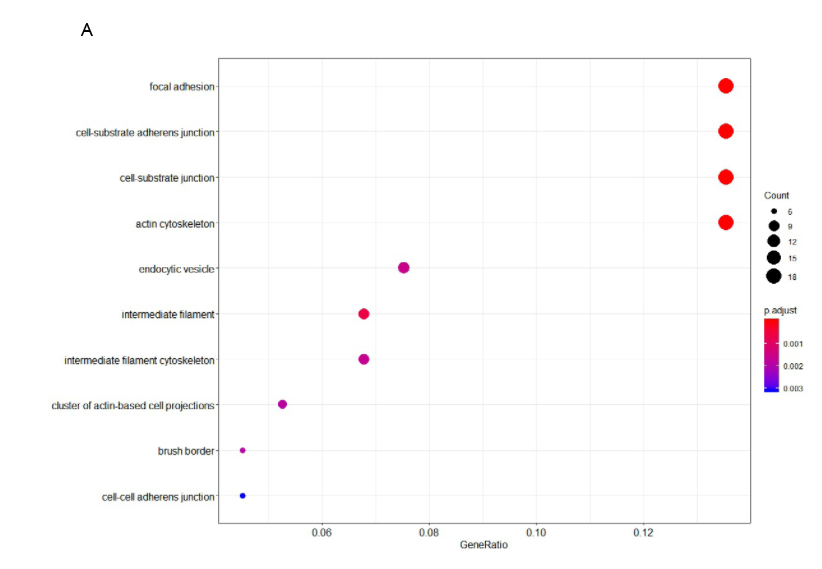


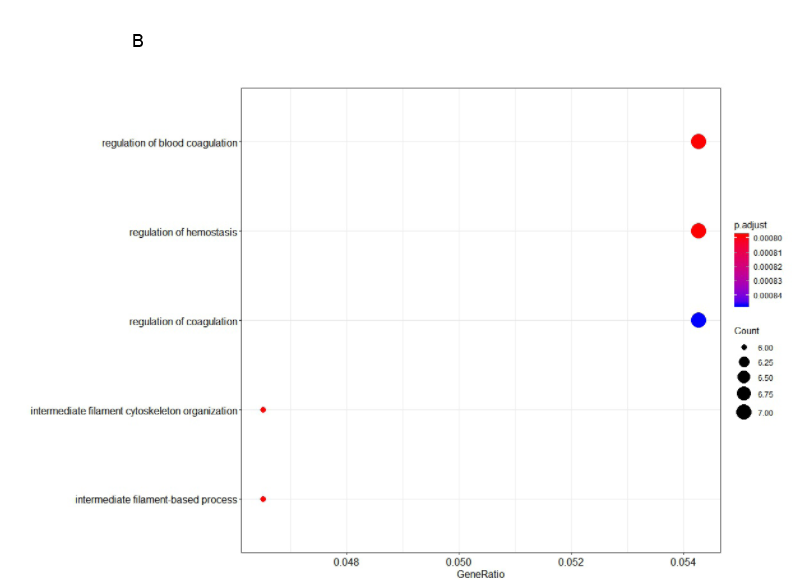


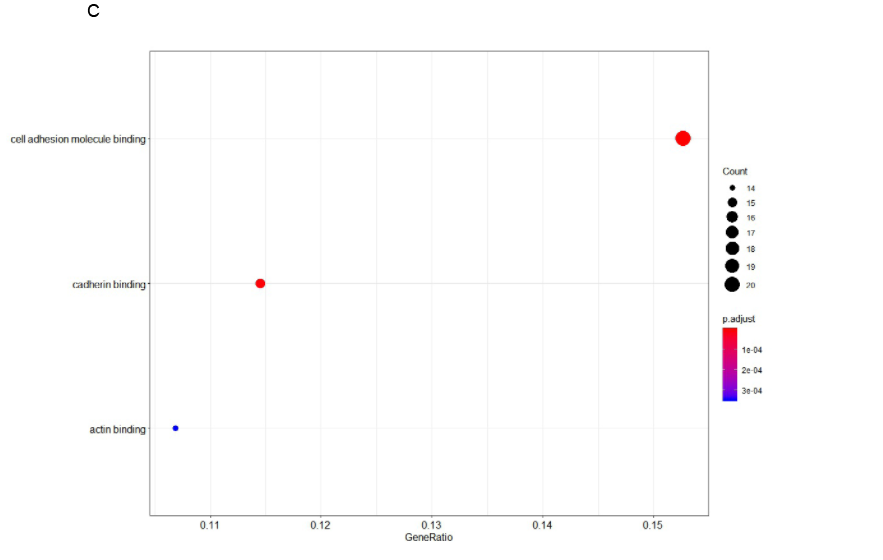


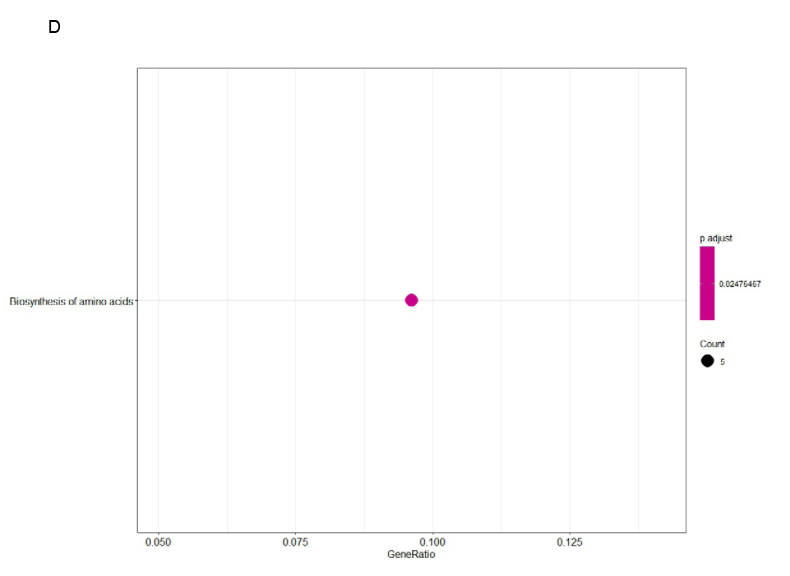


**Figure S5.** Western blotting of the expression of CKAP4 and CD44 in CD34^+^ primary cells after co-cultured with HS5 (A). The expression of LCP1 and CAPG in HS5 after co-cultured with CD34^+^ primary cells (B).


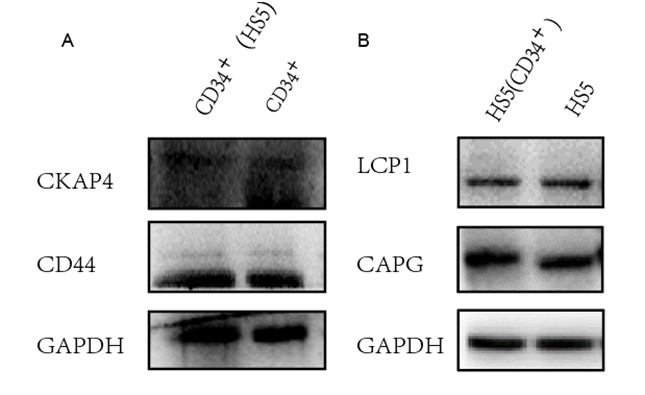


**Figure S6.** Flow cytometry analysis verified the sorted KG1a and HS5 cells after co-culture.


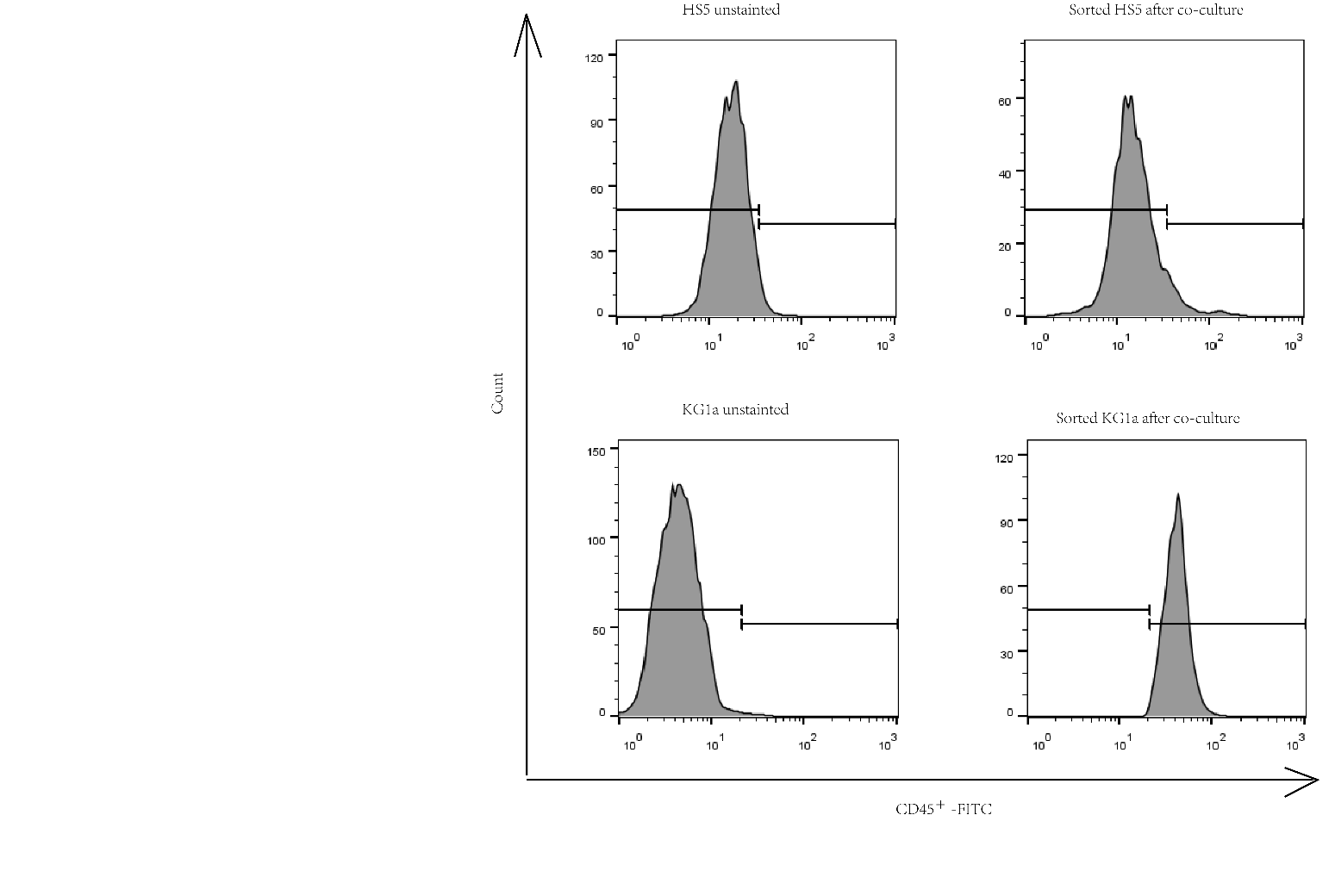


| **Table S1. Significant regulated proteins in KG1a after co-culture with HS5(Z-score ≥ 1.960σ).** | | | | | | | |
| --- | --- | --- | --- | --- | --- | --- | --- |
| Protein IDs | Majority protein IDs | Peptide counts (all) | Peptide counts (razor+unique) | Peptide  counts (unique) | Protein names | Gene names | Average Ratio H/M |
| P05120;H7C004;H7BYS2;E7EPJ9;E7ERB5;E9PDK7 | P05120 | 30;12;12;9;7;6 | 30;12;12;9;7;6 | 30;12;12;9;7;6 | Plasminogen activator inhibitor 2 | SERPINB2 | 382.27 |
| P02545-6;P02545;P02545-2;P02545-3;Q3BDU5;P02545-5;P02545-4;Q5TCI8;H0YAB0;A0A0C4DGC5 | P02545-6;P02545;P02545-2;P02545-3;Q3BDU5;P02545-5;P02545-4;Q5TCI8 | 35;35;34;34;30;27;27;26;11;8 | 35;35;34;34;30;27;27;26;11;8 | 35;35;34;34;30;27;27;26;11;8 | Prelamin-A/C;Lamin-A/C | LMNA | 319.07 |
| Q07065;H3BUW6 | Q07065 | 34;1 | 34;1 | 33;0 | Cytoskeleton-associated protein 4 | CKAP4 | 171.75 |
| P07355;P07355-2;H0YM50;H0YN42;H0YMD0;H0YMU9;A6NMY6;H0YNP5;H0YKS4;H0YN28;H0YL33;H0YMM1;H0YKZ7;H0YLV6;H0YMT9;H0YKX9;H0YMW4;H0YNA0;H0YKL9;H0YN52;H0YKV8;H0YMD9;H0YLE2;H0YNB8;H0YKN4 | P07355;P07355-2;H0YM50;H0YN42;H0YMD0;H0YMU9;A6NMY6;H0YNP5;H0YKS4 | 45;45;35;35;33;33;28;23;23;22;22;16;13;13;13;13;13;12;11;10;8;6;5;4;4 | 45;45;35;35;33;33;28;23;23;22;22;16;13;13;13;13;13;12;11;10;8;6;5;4;4 | 45;45;35;35;33;33;28;23;23;22;22;16;13;13;13;13;13;12;11;10;8;6;5;4;4 | Annexin A2;Annexin;Putative annexin A2-like protein | ANXA2;ANXA2P2 | 154.33 |
| Q09666;E9PQE3;E9PJZ0;E9PKR9;E9PJC6;E9PLK4;Q09666-2 | Q09666 | 118;1;1;1;1;1;1 | 118;1;1;1;1;1;1 | 118;1;1;1;1;1;1 | Neuroblast differentiation-associated protein AHNAK | AHNAK | 108.68 |
| P48307-2;P48307;H7C4A3 | P48307-2;P48307;H7C4A3 | 4;4;2 | 4;4;2 | 4;4;2 | Tissue factor pathway inhibitor 2 | TFPI2 | 20.66 |
| Q6ZN40;P09493-9;P09493-3;P09493-8;P09493-7;P09493-2 | Q6ZN40;P09493-9;P09493-3;P09493-8;P09493-7;P09493-2 | 26;26;26;22;22;21 | 5;5;5;5;5;4 | 0;0;0;0;0;0 | Tropomyosin alpha-1 chain | TPM1 | 12.35 |
| P06703;R4GN98 | P06703;R4GN98 | 10;9 | 10;9 | 10;9 | Protein S100-A6;Protein S100 | S100A6 | 11.96 |
| P35580;P35580-5;P35580-2;P35580-3;P35580-4 | P35580;P35580-5;P35580-2;P35580-3;P35580-4 | 35;35;35;35;35 | 6;6;6;6;6 | 6;6;6;6;6 | Myosin-10 | MYH10 | 8.18 |
| P09936;D6RE83 | P09936;D6RE83 | 10;9 | 10;9 | 1;1 | Ubiquitin carboxyl-terminal hydrolase isozyme L1;Ubiquitin carboxyl-terminal hydrolase | UCHL1 | 5.32 |
| Q99584 | Q99584 | 5 | 5 | 5 | Protein S100-A13 | S100A13 | 4.95 |
| P60903 | P60903 | 7 | 7 | 7 | Protein S100-A10 | S100A10 | 4.43 |
| Q9Y6N5;H3BNX3;H3BMS6;H3BNP9;A0A0A6YYU7;H3BV36 | Q9Y6N5;H3BNX3 | 8;4;3;2;2;2 | 8;4;3;2;2;2 | 8;4;3;2;2;2 | Sulfide:quinone oxidoreductase, mitochondrial | SQRDL | 4.01 |
| P07858;E9PR54;E9PJ67;E9PCB3;E9PKQ7;E9PNL5;E9PHZ5;E9PLY3;E9PSG5;E9PQM1;R4GMQ5;E9PS78 | P07858 | 10;3;3;3;3;3;3;3;3;3;2;2 | 10;3;3;3;3;3;3;3;3;3;2;2 | 10;3;3;3;3;3;3;3;3;3;2;2 | Cathepsin B;Cathepsin B light chain;Cathepsin B heavy chain | CTSB | 3.66 |
| A0A087X0S5;P12109 | A0A087X0S5;P12109 | 10;10 | 10;10 | 10;10 | Collagen alpha-1(VI) chain | COL6A1 | 3.35 |
| O00469-2;E7ETU9;O00469;O00469-3;C9JXZ0;F8WEW3 | O00469-2;E7ETU9;O00469;O00469-3 | 26;25;25;14;9;1 | 26;25;25;14;9;1 | 26;25;25;14;9;1 | Procollagen-lysine,2-oxoglutarate 5-dioxygenase 2 | PLOD2 | 3.29 |
| P13797;A0A0A0MSQ0;P13797-2;F8W8D8;P13797-3;H7C4N2;U3KQI3;C9JAM8 | P13797;A0A0A0MSQ0;P13797-2;F8W8D8;P13797-3 | 28;27;27;26;26;8;7;2 | 21;20;20;19;19;7;4;1 | 20;19;19;18;18;6;4;0 | Plastin-3 | PLS3 | 3.12 |
| O15460-2;O15460;C9JN43;C9JIG4;C9JCP0;C9JX45;A8MXE0;E7ERI1;E7ENX0;E7EPI9 | O15460-2;O15460 | 6;6;1;1;1;1;1;1;1;1 | 6;6;1;1;1;1;1;1;1;1 | 6;6;1;1;1;1;1;1;1;1 | Prolyl 4-hydroxylase subunit alpha-2 | P4HA2 | 2.93 |
| Q5XLA6 | Q5XLA6 | 4 | 4 | 4 | Caspase recruitment domain-containing protein 17 | CARD17 | 2.91 |
| O94855;O94855-2;E9PDM8;E9PC44 | O94855;O94855-2;E9PDM8 | 17;17;10;5 | 15;15;10;5 | 15;15;10;5 | Protein transport protein Sec24D | SEC24D | 2.63 |
| P21796;C9JI87 | P21796;C9JI87 | 15;8 | 15;8 | 15;8 | Voltage-dependent anion-selective channel protein 1 | VDAC1 | 2.51 |
| P45880;A0A0A0MR02;P45880-2;P45880-1;Q5JSD2;Q5JSD1;A2A3S1 | P45880;A0A0A0MR02;P45880-2;P45880-1;Q5JSD2;Q5JSD1 | 13;12;12;12;9;9;4 | 13;12;12;12;9;9;4 | 13;12;12;12;9;9;4 | Voltage-dependent anion-selective channel protein 2 | VDAC2 | 2.46 |
| Q96PP8;Q96PP8-2;E7ETN8 | Q96PP8;Q96PP8-2;E7ETN8 | 23;21;19 | 23;21;19 | 20;18;16 | Guanylate-binding protein 5 | GBP5 | 2.38 |
| Q92597;Q92597-3;Q92597-2;E7ESM1;E5RJY1;E5RIR1;E5RGM5;E5RIV1;E5RIM2;E5RI76;E5RH82;E5RK17;E5RJ98;E5RG99 | Q92597;Q92597-3;Q92597-2 | 10;8;8;4;3;3;3;3;3;2;2;2;1;1 | 10;8;8;4;3;3;3;3;3;2;2;2;1;1 | 10;8;8;4;3;3;3;3;3;2;2;2;1;1 | Protein NDRG1 | NDRG1 | 2.28 |
| A0A0A0MTJ9;Q6PIU2;Q6PIU2-2;H7C046;Q6PIU2-3 | A0A0A0MTJ9;Q6PIU2;Q6PIU2-2;H7C046;Q6PIU2-3 | 6;6;6;4;4 | 6;6;6;4;4 | 6;6;6;4;4 | Neutral cholesterol ester hydrolase 1 | NCEH1 | 2.26 |
| O60568;H7C2V1;H7C2S8;H7C0B8 | O60568;H7C2V1 | 11;7;4;3 | 11;7;4;3 | 11;7;4;3 | Procollagen-lysine,2-oxoglutarate 5-dioxygenase 3 | PLOD3 | 2.23 |
| P80723;P80723-2;U3KQP0 | P80723;P80723-2 | 15;11;3 | 15;11;3 | 15;11;3 | Brain acid soluble protein 1 | BASP1 | 2.07 |
| F8VU90;Q9NYL4-2;Q9NYL4;E9PAR0;H0YHM7 | F8VU90;Q9NYL4-2;Q9NYL4 | 6;6;6;2;1 | 6;6;6;2;1 | 6;6;6;2;1 | Peptidyl-prolyl cis-trans isomerase;Peptidyl-prolyl cis-trans isomerase FKBP11 | FKBP11 | 2.03 |
| P09525;Q6P452;P09525-2 | P09525;Q6P452;P09525-2 | 11;10;8 | 11;10;8 | 11;10;8 | Annexin A4;Annexin | ANXA4 | 1.96 |
| Q16222-2;Q16222-3;Q16222 | Q16222-2;Q16222-3;Q16222 | 18;17;17 | 18;17;17 | 16;15;15 | UDP-N-acetylhexosamine pyrophosphorylase;UDP-N-acetylgalactosamine pyrophosphorylase;UDP-N-acetylglucosamine pyrophosphorylase | UAP1 | 1.92 |
| P43490;A0A0C4DFS8;C9JF35;C9JG65 | P43490;A0A0C4DFS8 | 21;13;1;1 | 21;13;1;1 | 21;13;1;1 | Nicotinamide phosphoribosyltransferase | NAMPT | 1.89 |
| Q9BRT6 | Q9BRT6 | 5 | 5 | 5 | Protein LLP homolog | LLPH | 1.87 |
| P68366-2;P68366;C9JDS9;C9JEV8;C9JQ00;C9JJQ8;C9JDL2 | P68366-2;P68366 | 39;39;13;9;9;9;6 | 4;4;0;3;3;3;2 | 4;4;0;3;3;3;2 | Tubulin alpha-4A chain | TUBA4A | 1.82 |
| P18031;B4DSN5;REV__J3QSB5;REV__Q9Y3U8;REV__Q8IX04-6;REV__Q8IX04 | P18031;B4DSN5 | 22;17;1;1;1;1 | 22;17;1;1;1;1 | 22;17;1;1;1;1 | Tyrosine-protein phosphatase non-receptor type 1;Tyrosine-protein phosphatase non-receptor type | PTPN1 | 1.80 |
| P08107;P08107-2;V9GZ37 | P08107;P08107-2;V9GZ37 | 34;30;23 | 27;23;18 | 17;14;11 | Heat shock 70 kDa protein 1A | HSPA1A | 1.73 |
| Q96PP9;Q6ZN66 | Q96PP9 | 6;1 | 6;1 | 6;1 | Guanylate-binding protein 4 | GBP4 | 1.73 |
| Q96N66-2;Q96N66;Q96N66-3;M0R1Z5;A9C4B8 | Q96N66-2;Q96N66;Q96N66-3 | 5;5;4;1;1 | 5;5;4;1;1 | 5;5;4;1;1 | Lysophospholipid acyltransferase 7 | MBOAT7 | 1.73 |
| P32455;H3BNS1;F6X827;F6SPX6;H3BRX6;H3BP53;REV__CON__Q3TTY5;Q8N8V2 | P32455 | 21;4;4;2;1;1;1;1 | 20;4;3;1;0;0;1;0 | 11;0;0;0;0;0;0;0 | Interferon-induced guanylate-binding protein 1 | GBP1 | 1.60 |
| Q01201;D6R992 | Q01201;D6R992 | 6;5 | 6;5 | 6;5 | Transcription factor RelB | RELB | 1.56 |
| P11021;REV__H3BUZ0;REV__Q9NXD2-2;REV__Q9NXD2 | P11021 | 66;1;1;1 | 65;1;1;1 | 65;1;1;1 | 78 kDa glucose-regulated protein | HSPA5 | 1.53 |
| O60234;M0QYJ8;M0R0C1;M0QYG8;M0R1D2;M0QXC2;M0QX47;M0QXZ6;M0R2P2 | O60234;M0QYJ8;M0R0C1;M0QYG8;M0R1D2;M0QXC2 | 15;14;11;9;9;8;5;1;1 | 15;14;11;9;9;8;5;1;1 | 13;12;9;7;7;8;5;1;1 | Glia maturation factor gamma | GMFG | 0.66 |
| Q9Y5Q9;Q9Y5Q9-2;H7C0C0;F8WC64;H0YFI7 | Q9Y5Q9;Q9Y5Q9-2 | 7;4;2;1;1 | 7;4;2;1;1 | 7;4;2;1;1 | General transcription factor 3C polypeptide 3 | GTF3C3 | 0.65 |
| Q70J99;Q70J99-3;Q70J99-2;K7EN81;K7EMK8;K7EN29;K7EIH3;K7EM66 | Q70J99;Q70J99-3 | 33;33;10;9;6;5;4;3 | 33;33;10;9;6;5;4;3 | 23;23;0;0;6;5;0;2 | Protein unc-13 homolog D | UNC13D | 0.65 |
| P16150;C9JUK7 | P16150;C9JUK7 | 10;7 | 10;7 | 10;7 | Leukosialin | SPN | 0.64 |
| Q6UW63 | Q6UW63 | 10 | 10 | 10 | KDEL motif-containing protein 1 | KDELC1 | 0.64 |
| Q7Z4Q2;Q7Z4Q2-2;Q7Z4Q2-3 | Q7Z4Q2;Q7Z4Q2-2 | 7;5;2 | 7;5;2 | 7;5;2 | HEAT repeat-containing protein 3 | HEATR3 | 0.64 |
| A0A0A0MRM9;Q14978;Q14978-3;Q14978-2;S4R349;S4R402;S4R341;S4R3C2 | A0A0A0MRM9;Q14978;Q14978-3;Q14978-2;S4R349 | 9;9;9;9;5;2;2;1 | 9;9;9;9;5;2;2;1 | 9;9;9;9;5;2;2;1 | Nucleolar and coiled-body phosphoprotein 1 | NOLC1 | 0.63 |
| E9PNW4;E9PR17;P13987;H0YET2 | E9PNW4;E9PR17;P13987 | 5;5;5;2 | 5;5;5;2 | 5;5;5;2 | CD59 glycoprotein | CD59 | 0.63 |
| Q12789-3;Q12789;H3BU37 | Q12789-3;Q12789 | 11;11;1 | 11;11;1 | 11;11;1 | General transcription factor 3C polypeptide 1 | GTF3C1 | 0.62 |
| Q15758;M0QXM4;Q15758-3;Q15758-2;M0QX44;M0R144 | Q15758;M0QXM4;Q15758-3;Q15758-2 | 9;8;6;6;3;1 | 9;8;6;6;3;1 | 9;8;6;6;3;1 | Neutral amino acid transporter B(0);Amino acid transporter | SLC1A5 | 0.61 |
| P43405;P43405-2;K7ERY4;P43403-2;Q8NFD2 | P43405;P43405-2 | 40;37;1;1;1 | 40;37;1;1;1 | 38;35;0;0;0 | Tyrosine-protein kinase SYK | SYK | 0.61 |
| O95810 | O95810 | 11 | 11 | 11 | Serum deprivation-response protein | SDPR | 0.60 |
| O15327;D6RJC3;E7EQN9;E9PCZ3;E9PHC0;E9PG59;O15327-2;H0YA10;D6RE59 | O15327;D6RJC3;E7EQN9;E9PCZ3;E9PHC0 | 20;19;19;17;16;9;2;1;1 | 20;19;19;17;16;9;2;1;1 | 20;19;19;17;16;9;2;1;1 | Type II inositol 3,4-bisphosphate 4-phosphatase | INPP4B | 0.60 |
| Q01970;Q01970-2 | Q01970;Q01970-2 | 7;4 | 6;3 | 6;3 | 1-phosphatidylinositol 4,5-bisphosphate phosphodiesterase beta-3 | PLCB3 | 0.58 |
| A0FGR8-2;H7BXI1;A0A087WXU3;A0FGR8;A0FGR8-6;A0FGR8-4;A0FGR8-5;Q68CJ9-4;Q68CJ9-2;Q68CJ9 | A0FGR8-2;H7BXI1;A0A087WXU3;A0FGR8;A0FGR8-6;A0FGR8-4 | 33;32;32;32;31;17;15;1;1;1 | 33;32;32;32;31;17;15;1;1;1 | 33;32;32;32;31;17;15;1;1;1 | Extended synaptotagmin-2 | ESYT2 | 0.58 |
| Q9HBI0;Q9HBI0-2;Q9HBI0-3;B0QYM9;B0QYN0;U3KQ67;Q9HBI0-5;Q9HBI0-4 | Q9HBI0;Q9HBI0-2;Q9HBI0-3 | 10;9;6;1;1;1;1;1 | 10;9;6;1;1;1;1;1 | 10;9;6;1;1;1;1;1 | Gamma-parvin | PARVG | 0.58 |
| Q92692;Q92692-2;K7EKE8 | Q92692;Q92692-2 | 14;9;6 | 14;9;6 | 14;9;6 | Nectin-2 | PVRL2 | 0.57 |
| Q92614-4;Q92614;Q92614-2;Q92614-3;Q92614-5;H0YEV9 | Q92614-4;Q92614;Q92614-2;Q92614-3;Q92614-5 | 66;66;65;63;56;9 | 66;66;65;63;56;9 | 65;65;64;62;55;9 | Unconventional myosin-XVIIIa | MYO18A | 0.57 |
| P51531-2;P51531;F6VDE0;F6XE55;F6XG14;B1ALF6;A0A0A0MT03 | P51531-2;P51531 | 29;29;6;5;5;5;1 | 9;9;3;1;1;1;1 | 9;9;3;1;1;1;1 | Probable global transcription activator SNF2L2 | SMARCA2 | 0.56 |
| Q9BV40;B8ZZT4;C9JXZ5 | Q9BV40;B8ZZT4 | 3;2;1 | 3;2;1 | 3;2;1 | Vesicle-associated membrane protein 8 | VAMP8 | 0.56 |
| Q7LDG7;Q7LDG7-4;Q7LDG7-2;E7EM78;E7EMB4;E7EWQ7;A6NJ28;Q7LDG7-3;C9JZ82;A6NHE2;A8MVK8;A8MTF9 | Q7LDG7;Q7LDG7-4;Q7LDG7-2 | 29;29;29;8;6;6;6;6;4;4;1;1 | 29;29;29;8;6;6;6;6;4;4;1;1 | 29;29;29;8;6;6;6;6;4;4;1;1 | RAS guanyl-releasing protein 2 | RASGRP2 | 0.56 |
| Q9BZL1;K7EQ43 | Q9BZL1 | 7;1 | 7;1 | 7;1 | Ubiquitin-like protein 5 | UBL5 | 0.56 |
| P45973;F8VNY3 | P45973;F8VNY3 | 6;3 | 5;3 | 5;3 | Chromobox protein homolog 5 | CBX5 | 0.55 |
| P50749;P50749-2;Q9H2L5-3;Q9H2L5;Q9H2L5-2 | P50749 | 14;5;2;2;2 | 14;5;2;2;2 | 14;5;2;2;2 | Ras association domain-containing protein 2 | RASSF2 | 0.55 |
| Q8IV04;E9PSB7;Q8IV04-2 | Q8IV04 | 7;3;2 | 7;3;2 | 7;3;2 | Carabin | TBC1D10C | 0.55 |
| Q86W42-3;Q86W42;Q86W42-2 | Q86W42-3;Q86W42;Q86W42-2 | 6;6;4 | 6;6;4 | 6;6;4 | THO complex subunit 6 homolog | THOC6 | 0.54 |
| P08754;P19087;P11488;P09471;A8MTJ3;A0A087WTB6;A2A2R6;Q5JWD1;H0Y7E8;H3BTM2;A0A087WZE5 | P08754 | 12;3;2;2;2;1;1;1;1;1;1 | 4;0;0;0;0;0;0;0;0;0;0 | 4;0;0;0;0;0;0;0;0;0;0 | Guanine nucleotide-binding protein G(k) subunit alpha | GNAI3 | 0.54 |
| Q92686;P17677;P17677-2 | Q92686 | 3;1;1 | 3;1;1 | 3;1;1 | Neurogranin;NEUG(55-78) | NRGN | 0.54 |
| A6NJT0 | A6NJT0 | 9 | 9 | 9 | Homeobox protein unc-4 homolog | UNCX | 0.53 |
| G5E953;Q13615-3;Q13615-2;Q13615 | G5E953;Q13615-3;Q13615-2;Q13615 | 5;5;5;5 | 5;5;5;5 | 5;5;5;5 | Myotubularin-related protein 3 | MTMR3 | 0.53 |
| P16070-18;P16070-12;P16070-14;P16070-13;P16070-11;P16070-10;P16070-16;P16070-8;P16070-17;P16070-6;P16070-4;P16070-3;P16070-7;P16070-5;P16070;H0YD13;H0Y2P0;H0YDW7;H0YCV9;H0Y5E4;P16070-15;P16070-9;H0YE40;E9PKC6;H0YDX6;Q86UZ1;H0YD17;P16070-19;H0YD90;J3KN83;H0YEV3;H0YEU1;H0YF08 | P16070-18;P16070-12;P16070-14;P16070-13;P16070-11;P16070-10;P16070-16;P16070-8;P16070-17;P16070-6;P16070-4;P16070-3;P16070-7;P16070-5;P16070;H0YD13;H0Y2P0;H0YDW7;H0YCV9;H0Y5E4;P16070-15;P16070-9 | 18;18;18;18;18;18;18;18;18;18;18;18;18;18;18;15;14;10;10;10;10;10;8;6;6;5;5;5;3;3;3;3;1 | 18;18;18;18;18;18;18;18;18;18;18;18;18;18;18;15;14;10;10;10;10;10;8;6;6;5;5;5;3;3;3;3;1 | 18;18;18;18;18;18;18;18;18;18;18;18;18;18;18;15;14;10;10;10;10;10;8;6;6;5;5;5;3;3;3;3;1 | CD44 antigen | CD44 | 0.52 |
| P22087;M0QXL5;M0R299;M0R2Q4;M0R0P1;M0R2U2;M0R1H0;M0R2B0;M0QXC9 | P22087;M0QXL5;M0R299;M0R2Q4;M0R0P1;M0R2U2;M0R1H0;M0R2B0 | 18;17;15;14;13;11;11;11;1 | 18;17;15;14;13;11;11;11;1 | 15;14;12;12;11;10;10;10;1 | rRNA 2-O-methyltransferase fibrillarin | FBL | 0.51 |
| Q9Y6H1;Q5T1J5 | Q9Y6H1;Q5T1J5 | 6;5 | 6;5 | 6;5 | Coiled-coil-helix-coiled-coil-helix domain-containing protein 2;Putative coiled-coil-helix-coiled-coil-helix domain-containing protein CHCHD2P9, mitochondrial | CHCHD2;CHCHD2P9 | 0.51 |
| P37268;E9PNM1;P37268-2;P37268-3;P37268-5;P37268-4;E9PJG4;E9PS69;E9PQ90;E9PNJ2;E9PSH1 | P37268;E9PNM1;P37268-2;P37268-3;P37268-5;P37268-4 | 20;19;16;15;15;13;9;7;1;1;1 | 20;19;16;15;15;13;9;7;1;1;1 | 20;19;16;15;15;13;9;7;1;1;1 | Squalene synthase | FDFT1 | 0.51 |
| P27701;P27701-2;E9PJC7 | P27701;P27701-2;E9PJC7 | 5;4;3 | 5;4;3 | 5;4;3 | CD82 antigen;Tetraspanin | CD82 | 0.50 |
| O00161;H3BV99;H3BP15;H3BQY9;O00161-2;H3BM38;H3BNE1;H3BR18;H3BPJ0;H3BR99;H3BNG6;H3BU94 | O00161;H3BV99;H3BP15;H3BQY9;O00161-2 | 11;7;7;7;7;5;4;4;4;2;1;1 | 11;7;7;7;7;5;4;4;4;2;1;1 | 11;7;7;7;7;5;4;4;4;2;1;1 | Synaptosomal-associated protein 23;Synaptosomal-associated protein | SNAP23 | 0.49 |
| P14209;A0A096LP69;P14209-3;A8MQT7;H7C2F2;P14209-2 | P14209;A0A096LP69;P14209-3;A8MQT7;H7C2F2;P14209-2 | 5;4;4;3;3;3 | 5;4;4;3;3;3 | 5;4;4;3;3;3 | CD99 antigen | CD99 | 0.49 |
| K7EKM7;O75264 | K7EKM7;O75264 | 4;4 | 4;4 | 4;4 | Small integral membrane protein 24 | SMIM24 | 0.49 |
| Q99622;U3KQ85;F5GXW5;U3KQ07 | Q99622;U3KQ85;F5GXW5 | 3;2;2;1 | 3;2;2;1 | 3;2;2;1 | Protein C10 | C12orf57 | 0.48 |
| Q13601;Q13601-2;A0A087WZC7 | Q13601;Q13601-2;A0A087WZC7 | 6;5;3 | 6;5;3 | 6;5;3 | KRR1 small subunit processome component homolog | KRR1 | 0.48 |
| Q12788;J3KNP2;A0A087WYP7 | Q12788;J3KNP2 | 6;5;1 | 6;5;1 | 6;5;1 | Transducin beta-like protein 3 | TBL3 | 0.47 |
| B4DGU4;P35222;P35222-2;E7EMJ5 | B4DGU4;P35222 | 14;14;3;1 | 14;14;3;1 | 11;11;2;1 | Catenin beta-1 | CTNNB1 | 0.47 |
| Q8TCZ2-3;Q8TCZ2-2;Q8TCZ2;Q8TCZ2-5;H0Y4H3;Q8TCZ2-6 | Q8TCZ2-3;Q8TCZ2-2;Q8TCZ2;Q8TCZ2-5;H0Y4H3;Q8TCZ2-6 | 4;4;4;4;2;2 | 4;4;4;4;2;2 | 4;4;4;4;2;2 | CD99 antigen-like protein 2 | CD99L2 | 0.47 |
| J3QRU1;P07947;P12931;P12931-2 | J3QRU1;P07947 | 8;8;3;3 | 6;6;1;1 | 6;6;1;1 | Non-specific protein-tyrosine kinase;Tyrosine-protein kinase Yes | YES1 | 0.46 |
| A0A0C4DFL7;Q16850;Q16850-2;H7C0D0;C9IYR8 | A0A0C4DFL7;Q16850;Q16850-2 | 10;10;7;4;1 | 10;10;7;4;1 | 10;10;7;4;1 | Lanosterol 14-alpha demethylase | CYP51A1 | 0.45 |
| O00567;Q5JXT2;H0YDU4;H0Y653 | O00567;Q5JXT2;H0YDU4 | 20;11;10;9 | 20;11;10;9 | 20;11;10;9 | Nucleolar protein 56 | NOP56 | 0.45 |
| Q9Y2X3;H7BZ72;F8WED0 | Q9Y2X3 | 14;5;1 | 14;5;1 | 14;5;1 | Nucleolar protein 58 | NOP58 | 0.44 |
| O95816;O95816-2;Q9UL42 | O95816;O95816-2 | 11;10;1 | 11;10;1 | 10;9;0 | BAG family molecular chaperone regulator 2 | BAG2 | 0.44 |
| P56202;E9PI30;H0YDT2;J3QLB4;D6RAR2;D6RFL2;J3KTA2;D6RHE0;A0A087WSZ7;A8MSI8;Q08499-5;Q08499-8;Q08499-4;Q08499-3;Q08499-2;Q08499-10;Q08499-9;Q08499-11;Q08499 | P56202;E9PI30;H0YDT2 | 20;16;10;1;1;1;1;1;1;1;1;1;1;1;1;1;1;1;1 | 20;16;10;1;1;1;1;1;1;1;1;1;1;1;1;1;1;1;1 | 19;15;10;0;0;0;0;0;0;0;0;0;0;0;0;0;0;0;0 | Cathepsin W | CTSW | 0.44 |
| B0I1T2;B0I1T2-4;B0I1T2-3;F8WEW9;B0I1T2-2;J3KRL0 | B0I1T2;B0I1T2-4 | 57;35;21;7;7;2 | 57;35;21;7;7;2 | 55;33;19;5;5;0 | Unconventional myosin-Ig;Minor histocompatibility antigen HA-2 | MYO1G | 0.43 |
| Q9NUN5-4;Q9NUN5-2;Q9NUN5-3;Q9NUN5 | Q9NUN5-4;Q9NUN5-2;Q9NUN5-3;Q9NUN5 | 1;1;1;1 | 1;1;1;1 | 1;1;1;1 | Probable lysosomal cobalamin transporter | LMBRD1 | 0.42 |
| P26447 | P26447 | 8 | 8 | 8 | Protein S100-A4 | S100A4 | 0.41 |
| Q9Y605;A0A087WTY9 | Q9Y605;A0A087WTY9 | 2;1 | 2;1 | 2;1 | MORF4 family-associated protein 1 | MRFAP1 | 0.41 |
| O60262 | O60262 | 2 | 2 | 2 | Guanine nucleotide-binding protein G(I)/G(S)/G(O) subunit gamma-7 | GNG7 | 0.38 |
| P13591;A0A087WX77;A0A087WV75;P13591-5;A0A087WWJ5 | P13591;A0A087WX77;A0A087WV75;P13591-5 | 37;36;34;24;15 | 37;36;34;24;15 | 2;2;0;2;0 | Neural cell adhesion molecule 1 | NCAM1 | 0.38 |
| O95864;O95864-3;O95864-4;O95864-2;E5RGD8;E9PPZ4;E9PQC2;E9PKP8 | O95864;O95864-3;O95864-4;O95864-2 | 17;14;11;11;2;1;1;1 | 17;14;11;11;2;1;1;1 | 11;8;7;7;0;0;0;0 | Fatty acid desaturase 2 | FADS2 | 0.38 |
| P62805 | P62805 | 13 | 13 | 13 | Histone H4 | HIST1H4A | 0.38 |
| Q9NWQ8 | Q9NWQ8 | 18 | 18 | 18 | Phosphoprotein associated with glycosphingolipid-enriched microdomains 1 | PAG1 | 0.36 |
| E9PJK1;E9PRJ8;H0YDL9;H0YDJ9;E9PIF1;A6NMH8;P60033;E9PM31;H0YEE2 | E9PJK1;E9PRJ8;H0YDL9;H0YDJ9;E9PIF1;A6NMH8;P60033 | 3;3;3;3;3;3;3;1;1 | 3;3;3;3;3;3;3;1;1 | 3;3;3;3;3;3;3;1;1 | Tetraspanin;CD81 antigen | CD81 | 0.35 |
| P68431 | P68431 | 11 | 11 | 1 | Histone H3.1 | HIST1H3A | 0.34 |
| Q658P3-3;Q658P3;Q658P3-2;B8ZZX6;Q658P3-4 | Q658P3-3;Q658P3;Q658P3-2;B8ZZX6;Q658P3-4 | 4;4;4;3;3 | 4;4;4;3;3 | 4;4;4;3;3 | Metalloreductase STEAP3 | STEAP3 | 0.34 |
| O43736-2;O43736 | O43736-2;O43736 | 10;10 | 10;10 | 10;10 | Integral membrane protein 2A | ITM2A | 0.33 |
| H0YKX3;Q15004;H0YMA4 | H0YKX3;Q15004;H0YMA4 | 4;4;3 | 4;4;3 | 4;4;3 | PCNA-associated factor | KIAA0101 | 0.31 |
| P07948;E5RJ37;C9JM54;C9J6K3 | P07948 | 27;8;1;1 | 27;8;1;1 | 3;3;0;0 | Tyrosine-protein kinase Lyn | LYN | 0.30 |
| Q6YHK3-4;Q6YHK3;Q6YHK3-2;Q6YHK3-3 | Q6YHK3-4;Q6YHK3;Q6YHK3-2;Q6YHK3-3 | 12;12;10;8 | 12;12;10;8 | 12;12;10;8 | CD109 antigen | CD109 | 0.25 |

**Table S2. The top 10 upregulated proteins in KG1a after co-culture with HS5.**

| Gene names | Protein descriptions | Mean SILAC ratio($\frac{\mathbf{Heavy(H)}}{\mathbf{Medium(M)}}$) |
| --- | --- | --- |
| SERPINB2 | Plasminogen activator inhibitor 2 | 382.27 |
| LMNA | Prelamin-A/C;Lamin-A/C | 319.07 |
| CKAP4 | Cytoskeleton-associated protein 4 | 171.75 |
| ANXA2;ANXA2P2 | Annexin A2;Annexin;Putative annexin A2-like protein | 154.33 |
| AHNAK | Neuroblast differentiation-associated protein AHNAK | 108.68 |
| TFPI2 | Tissue factor pathway inhibitor 2 | 20.66 |
| TPM1 | Tropomyosin alpha-1 chain | 12.35 |
| S100A6 | Protein S100-A6;Protein S100 | 11.96 |
| MYH10 | Myosin-10 | 8.18 |

**Table S3. The top 10 downregulated proteins in KG1a after co-culture with HS5.**

| Gene names | Protein descriptions | Mean SILAC ratio($\frac{\mathbf{Heavy(H)}}{\mathbf{Medium(M)}}$) |
| --- | --- | --- |
| FADS2 | Fatty acid desaturase 2 | 0.38 |
| HIST1H4A | Histone H4 | 0.38 |
| PAG1 | Phosphoprotein associated with glycosphingolipid-enriched microdomains 1 | 0.36 |
| CD81 | Tetraspanin;CD81 antigen | 0.35 |
| HIST1H3A | Histone H3.1 | 0.34 |
| STEAP3 | Metalloreductase STEAP3 | 0.34 |
| ITM2A | Integral membrane protein 2A | 0.33 |
| KIAA0101 | PCNA-associated factor | 0.31 |
| LYN | Tyrosine-protein kinase Lyn | 0.30 |
| CD109 | CD109 antigen | 0.25 |

**Table S4. Significant regulated proteins in HS5 after co-culture with KG1a (Z-score ≥ 1.960σ).**

| Protein IDs | Majority protein IDs | Peptide counts (all) | Peptide counts (razor+unique) | Peptide counts (unique) | Protein names | Gene names | Average ratio H/L |
| --- | --- | --- | --- | --- | --- | --- | --- |
| P81605;P81605-2 | P81605;P81605-2 | 4;4 | 4;4 | 4;4 | Dermcidin;Survival-promoting peptide;DCD-1 | DCD | 0.04 |
| P04264 | P04264 | 59 | 59 | 4 | Keratin, type II cytoskeletal 1 | KRT1 | 0.04 |
| P00750;P00750-3;E7ESF4;B4DNJ1;B4DN26;P00750-4;P00750-2;E5RHG4;H0YBH9 | P00750;P00750-3;E7ESF4;B4DNJ1;B4DN26;P00750-4 | 18;16;15;14;14;13;8;4;3 | 18;16;15;14;14;13;8;4;3 | 14;14;13;12;12;12;4;0;1 | Tissue-type plasminogen activator;Tissue-type plasminogen activator chain A;Tissue-type plasminogen activator chain B | PLAT | 0.08 |
| D3YTG3;H0Y897;Q7Z7G0-4;Q5JPC9;Q7Z7G0-3;H0YEA0;H7C556;E9PPR9;H7C4X4;H7C4S3;H7C4T1;H0YEL2;H0YCP4;H0YF57;H0YCG4 | D3YTG3;H0Y897;Q7Z7G0-4;Q5JPC9 | 35;19;19;18;17;8;6;5;5;5;3;3;1;1;1 | 35;19;19;18;17;8;6;5;5;5;3;3;1;1;1 | 1;1;1;1;0;0;0;0;0;1;0;0;0;0;0 | Target of Nesh-SH3 | ABI3BP;DKFZp667H216 | 0.17 |
| O75635-2;O75635;C9JM00;C9JA68;A0A0A6YYQ4 | O75635-2;O75635;C9JM00 | 9;9;5;4;4 | 9;9;5;4;4 | 9;9;5;4;4 | Serpin B7 | SERPINB7 | 0.18 |
| Q16352;A0A087WYG8;E7EMV2;E7ESP9;P07197-2;P07197 | Q16352;A0A087WYG8 | 14;13;3;3;3;3 | 12;11;1;1;1;1 | 11;10;0;0;0;0 | Alpha-internexin | INA | 0.21 |
| B1AHR1;Q9UH03-2;Q9UH03 | B1AHR1;Q9UH03-2;Q9UH03 | 5;5;5 | 4;4;4 | 4;4;4 | Neuronal-specific septin-3 | SEPT3 | 0.21 |
| P52943;P52943-2;H0YFA4;H0YHD8 | P52943;P52943-2;H0YFA4 | 9;9;7;3 | 9;9;7;3 | 9;9;7;3 | Cysteine-rich protein 2 | CRIP2 | 0.22 |
| O00391;O00391-2;H0Y5Z8;A8MXT8 | O00391;O00391-2 | 9;5;4;1 | 9;5;4;1 | 9;5;4;1 | Sulfhydryl oxidase 1 | QSOX1 | 0.22 |
| P08473;C9JR96;C9J9X7;C9IYX7;C9JDZ3;A0A087WWM7;Q3KQS6 | P08473 | 28;13;10;7;4;2;2 | 28;13;10;7;4;2;2 | 28;13;10;7;4;2;2 | Neprilysin | MME | 0.23 |
| P17676-2;P17676;P17676-3 | P17676-2;P17676 | 10;10;4 | 9;9;3 | 9;9;3 | CCAAT/enhancer-binding protein beta | CEBPB | 0.27 |
| Q9Y6K8-3;Q9Y6K8;Q9Y6K8-2;E9PQQ8;E9PIS7 | Q9Y6K8-3;Q9Y6K8 | 11;11;5;3;1 | 11;11;5;3;1 | 10;10;4;3;1 | Adenylate kinase isoenzyme 5 | AK5 | 0.29 |
| Q9NZN4;Q9NZN4-2 | Q9NZN4;Q9NZN4-2 | 31;22 | 30;22 | 26;18 | EH domain-containing protein 2 | EHD2 | 0.30 |
| G3V2N3;P54821-2;P54821;Q99811 | G3V2N3;P54821-2;P54821 | 6;6;6;2 | 6;6;6;2 | 6;6;6;2 | Paired mesoderm homeobox protein 1 | PRRX1 | 0.30 |
| Q07954;Q6PJ72;H0YJI8;Q7Z7K9;Q07954-2;H0YJ88 | Q07954 | 34;6;3;3;3;1 | 34;6;3;3;3;1 | 33;6;3;3;3;1 | Prolow-density lipoprotein receptor-related protein 1;Low-density lipoprotein receptor-related protein 1 85 kDa subunit;Low-density lipoprotein receptor-related protein 1 515 kDa subunit;Low-density lipoprotein receptor-related protein 1 intracellular domain | LRP1 | 0.30 |
| Q8IWE2;Q8IWE2-2;D6R9C9 | Q8IWE2;Q8IWE2-2 | 18;15;1 | 18;15;1 | 18;15;1 | Protein NOXP20 | FAM114A1 | 0.31 |
| Q9NYL2-2 | Q9NYL2-2 | 16 | 3 | 3 | Mitogen-activated protein kinase kinase kinase MLT | ZAK | 0.31 |
| G3V5X4;A0A0A0MRE3;Q8WXH0;Q8WXH0-2;A0A0C4DGK3;Q8WXH0-7;Q8WXH0-4;Q8WXH0-3;Q8WXH0-13;A0A0C4DH87;Q8WXH0-10 | G3V5X4;A0A0A0MRE3;Q8WXH0;Q8WXH0-2;A0A0C4DGK3;Q8WXH0-7 | 6;6;6;6;4;3;2;2;2;1;1 | 6;6;6;6;4;3;2;2;2;1;1 | 6;6;6;6;4;3;2;2;2;1;1 | Nesprin-2 | SYNE2 | 0.31 |
| P00966;Q5T6L6;Q5T6L5 | P00966;Q5T6L6;Q5T6L5 | 17;12;11 | 17;12;11 | 17;12;11 | Argininosuccinate synthase | ASS1 | 0.32 |
| Q14764;H3BQK6;H3BRL2;H3BUK7;H3BNF6;I3L155;H3BPZ2;H3BNF2;H3BUP3;H3BQE7 | Q14764 | 40;11;8;5;5;2;1;1;1;1 | 40;11;8;5;5;2;1;1;1;1 | 36;7;4;1;1;2;0;0;0;0 | Major vault protein | MVP | 0.32 |
| P48681 | P48681 | 39 | 39 | 39 | Nestin | NES | 0.32 |
| P08670;B0YJC4;B0YJC5;Q5JVS8;P17661;P14136-3;P14136;P14136-2;U3KPR1;B4DIR1;K7ELP4;K7EKH6;K7EJU1;K7EKH9 | P08670;B0YJC4 | 70;66;34;23;8;2;2;2;1;1;1;1;1;1 | 70;66;34;23;8;2;2;2;1;1;1;1;1;1 | 62;58;27;22;4;1;1;1;0;0;0;1;0;0 | Vimentin | VIM | 0.33 |
| P02545;P02545-6;P02545-3;P02545-5;P02545-4;H0YAB0;A0A0C4DGC5;REV__Q5VX52 | P02545;P02545-6;P02545-3;P02545-5;P02545-4 | 66;64;63;55;55;21;21;1 | 66;64;63;55;55;21;21;1 | 4;2;3;4;4;0;3;0 | Prelamin-A/C;Lamin-A/C | LMNA | 0.33 |
| P35580;P35580-3;P35580-5;P35580-2;P35580-4;E7ERA5 | P35580;P35580-3;P35580-5;P35580-2;P35580-4 | 94;94;93;93;93;6 | 65;65;64;64;64;3 | 62;62;61;61;61;3 | Myosin-10 | MYH10 | 0.33 |
| P02545-2;Q3BDU5;Q5TCI8 | P02545-2;Q3BDU5;Q5TCI8 | 63;55;52 | 1;1;1 | 1;1;1 | Prelamin-A/C;Lamin-A/C | LMNA | 0.34 |
| P05120;H7C004;H7BYS2;E7EPJ9;E7ERB5;E9PDK7 | P05120 | 28;13;13;7;6;5 | 28;13;13;7;6;5 | 28;13;13;7;6;5 | Plasminogen activator inhibitor 2 | SERPINB2 | 0.34 |
| D3TTY5;A0A087WU80;P21580;A0A087WXL5;A0A087X1N1;A0A087WVN3 | D3TTY5;A0A087WU80;P21580;A0A087WXL5;A0A087X1N1 | 5;5;5;4;4;1 | 5;5;5;4;4;1 | 5;5;5;4;4;1 | Tumor necrosis factor alpha-induced protein 3;A20p50;A20p37 | TNFAIP3 | 0.34 |
| O75508;B4DFI2 | O75508;B4DFI2 | 3;2 | 3;2 | 3;2 | Claudin-11;Claudin | CLDN11 | 0.35 |
| Q9Y5Z4;Q9Y5Z4-2;Q5THN1 | Q9Y5Z4;Q9Y5Z4-2 | 4;3;1 | 4;3;1 | 4;3;1 | Heme-binding protein 2 | HEBP2 | 0.35 |
| Q14692 | Q14692 | 6 | 6 | 6 | Ribosome biogenesis protein BMS1 homolog | BMS1 | 0.35 |
| P41226 | P41226 | 4 | 3 | 3 | Ubiquitin-like modifier-activating enzyme 7 | UBA7 | 0.36 |
| P52630;P52630-4;B4DLC8 | P52630;P52630-4 | 16;15;7 | 16;15;7 | 16;15;7 | Signal transducer and activator of transcription 2 | STAT2 | 0.36 |
| P47895;H0Y2X5;H0YNQ3;H0YKF9;H0YLT1;P00352 | P47895;H0Y2X5 | 23;20;8;8;1;1 | 23;20;8;8;1;1 | 19;17;7;6;1;0 | Aldehyde dehydrogenase family 1 member A3 | ALDH1A3 | 0.36 |
| P60660;F8W1R7;J3KND3;G3V1V0;B7Z6Z4;G3V1Y7;F8VPF3;H0YI43;F8W1I5 | P60660;F8W1R7;J3KND3;G3V1V0;B7Z6Z4;G3V1Y7;F8VPF3 | 10;9;9;9;8;7;7;2;2 | 1;0;1;0;0;1;0;0;0 | 1;0;1;0;0;1;0;0;0 | Myosin light polypeptide 6 | MYL6 | 0.36 |
| P23497;E9PHV6;P23497-7;P23497-6;P23497-5;P23497-2;P23497-3;P23497-4;C9JBL0;H7C4B4;H7BYP4;U5Y3L1;Q9H930-1;Q9H930-2;Q9H930;Q9H930-3 | P23497;E9PHV6;P23497-7;P23497-6;P23497-5;P23497-2;P23497-3;P23497-4;C9JBL0 | 17;13;13;13;13;13;13;13;9;2;2;2;2;2;2;1 | 13;13;13;13;13;13;13;13;9;2;2;2;2;2;2;1 | 13;13;13;13;13;13;13;13;9;2;2;2;2;2;2;1 | Nuclear autoantigen Sp-100 | SP100 | 0.36 |
| A0A0A0MT30;Q04828;H0Y804;A6NHU4;P52895-2;P17516 | A0A0A0MT30;Q04828;H0Y804 | 7;7;6;3;1;1 | 7;7;6;3;1;1 | 1;1;1;1;0;0 | Aldo-keto reductase family 1 member C1 | AKR1C1 | 0.37 |
| B1AHL2;P23142-2;P23142-3;P23142-4;P23142;H7C1M6;B1AHM7;B1AHM9;B1AHM8;CON__ENSEMBL:ENSBTAP00000016046 | B1AHL2;P23142-2;P23142-3;P23142-4;P23142;H7C1M6;B1AHM7;B1AHM9 | 6;6;6;6;6;4;3;3;1;1 | 6;6;6;6;6;4;3;3;1;1 | 6;6;6;6;6;4;3;3;1;1 | Fibulin-1 | FBLN1 | 0.37 |
| Q6P9B6;H3BM75;H3BUB0 | Q6P9B6 | 6;2;1 | 6;2;1 | 6;2;1 | TLD domain-containing protein 1 | TLDC1 | 0.37 |
| O94760;O94760-2 | O94760;O94760-2 | 19;14 | 19;14 | 18;13 | N(G),N(G)-dimethylarginine dimethylaminohydrolase 1 | DDAH1 | 0.37 |
| P01584;C9JVK0;C9JSC2;C9JWV2 | P01584 | 9;3;3;3 | 9;3;3;3 | 9;3;3;3 | Interleukin-1 beta | IL1B | 0.38 |
| P46937-2;P46937-8;P46937;P46937-9;P46937-5;P46937-3;P46937-6;P46937-7;P46937-4;H0YCI3 | P46937-2;P46937-8;P46937;P46937-9;P46937-5;P46937-3;P46937-6;P46937-7;P46937-4;H0YCI3 | 14;14;14;14;13;13;13;13;8;7 | 13;13;13;13;12;12;12;12;7;6 | 13;13;13;13;12;12;12;12;7;6 | Transcriptional coactivator YAP1 | YAP1 | 0.38 |
| P15924;P15924-2;P15924-3 | P15924;P15924-2;P15924-3 | 25;23;23 | 25;23;23 | 25;23;23 | Desmoplakin | DSP | 0.38 |
| A0A087WY00;F8W6H6;Q9Y4I1-2;Q9Y4I1;G3V394;F8WE88;Q9Y4I1-3;E7ERV5;Q9UES5;O95317;H0YMK3;Q7Z7A5;Q9NQX4-2;Q9ULV0;G3V3C9;H0YM93;H0YM96 | A0A087WY00;F8W6H6;Q9Y4I1-2;Q9Y4I1;G3V394;F8WE88;Q9Y4I1-3 | 27;27;27;27;26;26;26;8;2;2;2;2;2;2;1;1;1 | 27;27;27;27;26;26;26;8;2;2;2;2;2;2;1;1;1 | 25;25;25;25;24;24;24;8;2;2;0;1;0;1;1;0;0 | Unconventional myosin-Va | MYO5A | 0.38 |
| P15407;E9PKL5;E9PPX2 | P15407 | 4;1;1 | 4;1;1 | 4;1;1 | Fos-related antigen 1 | FOSL1 | 0.39 |
| Q5VUC6;O60551 | Q5VUC6;O60551 | 10;10 | 4;4 | 4;4 | Glycylpeptide N-tetradecanoyltransferase;Glycylpeptide N-tetradecanoyltransferase 2 | NMT2 | 0.39 |
| Q9BZQ8;H0Y7M9 | Q9BZQ8 | 36;2 | 36;2 | 36;2 | Protein Niban | FAM129A | 0.39 |
| A0A0A0MST8;E9PFN4;Q9Y6M7-9;Q9Y6M7-6;Q9Y6M7-13;Q9Y6M7-12;Q9Y6M7-8;Q9Y6M7-7;H7C3C4;C9JRP1;Q9Y6M7-11;Q9Y6M7-14;Q9Y6M7-10;Q9Y6M7-5;Q9Y6M7-4;Q9Y6M7-3;Q9Y6M7-2;Q9Y6M7;E7EW28;C9J240;Q2Y0W8-4;Q2Y0W8-5;Q2Y0W8-3;Q2Y0W8-2;Q6U841-2;Q2Y0W8;Q6U841-3;Q6U841 | A0A0A0MST8;E9PFN4;Q9Y6M7-9;Q9Y6M7-6;Q9Y6M7-13;Q9Y6M7-12;Q9Y6M7-8;Q9Y6M7-7;H7C3C4;C9JRP1;Q9Y6M7-11;Q9Y6M7-14;Q9Y6M7-10;Q9Y6M7-5;Q9Y6M7-4;Q9Y6M7-3;Q9Y6M7-2;Q9Y6M7 | 4;4;4;4;4;4;4;4;3;3;3;3;3;3;3;3;3;3;1;1;1;1;1;1;1;1;1;1 | 4;4;4;4;4;4;4;4;3;3;3;3;3;3;3;3;3;3;1;1;1;1;1;1;1;1;1;1 | 4;4;4;4;4;4;4;4;3;3;3;3;3;3;3;3;3;3;1;1;1;1;1;1;1;1;1;1 | Anion exchange protein;Sodium bicarbonate cotransporter 3 | SLC4A7 | 0.39 |
| P17096;P17096-3;E5RIT9 | P17096;P17096-3 | 10;6;1 | 10;6;1 | 3;3;0 | High mobility group protein HMG-I/HMG-Y | HMGA1 | 0.40 |
| O95340;O95340-2 | O95340;O95340-2 | 34;34 | 34;34 | 32;32 | Bifunctional 3-phosphoadenosine 5-phosphosulfate synthase 2;Sulfate adenylyltransferase;Adenylyl-sulfate kinase | PAPSS2 | 0.40 |
| Q8NEU8;Q8NEU8-3;Q8NEU8-2;F8VXB0;F8VS86;F8VWV2;F8W124;H0YH86 | Q8NEU8;Q8NEU8-3;Q8NEU8-2 | 14;13;12;6;3;3;1;1 | 14;13;12;6;3;3;1;1 | 14;13;12;6;3;3;1;1 | DCC-interacting protein 13-beta | APPL2 | 0.41 |
| Q5H9A7;P01033;H0Y789;Q5H9B5;Q5H9B4 | Q5H9A7;P01033;H0Y789 | 6;6;3;2;1 | 6;6;3;2;1 | 6;6;3;2;1 | Metalloproteinase inhibitor 1 | TIMP1 | 0.41 |
| P07951-2;P07951-3 | P07951-2;P07951-3 | 34;27 | 3;3 | 0;0 | Tropomyosin beta chain | TPM2 | 0.41 |
| Q86X27;Q86X27-3;Q86X27-2;A0A0A0MR31;H7C2H2 | Q86X27;Q86X27-3 | 9;8;4;3;1 | 9;8;4;3;1 | 9;8;4;3;1 | Ras-specific guanine nucleotide-releasing factor RalGPS2 | RALGPS2 | 0.41 |
| P05412 | P05412 | 5 | 5 | 5 | Transcription factor AP-1 | JUN | 0.41 |
| O14763-2;O14763 | O14763-2;O14763 | 3;3 | 3;3 | 3;3 | Tumor necrosis factor receptor superfamily member 10B | TNFRSF10B | 0.42 |
| Q9H6S3;Q9H6S3-3;Q9H6S3-2;A0A087WX39;E9PN68;E9PLN2;E9PNT0;E9PLH1;H0YD62;H0YF37 | Q9H6S3;Q9H6S3-3;Q9H6S3-2 | 17;16;9;5;1;1;1;1;1;1 | 17;16;9;5;1;1;1;1;1;1 | 17;16;9;5;1;1;1;1;1;1 | Epidermal growth factor receptor kinase substrate 8-like protein 2 | EPS8L2 | 0.42 |
| A0A0B4J2E5;Q15269 | A0A0B4J2E5;Q15269 | 1;1 | 1;1 | 1;1 | Periodic tryptophan protein 2 homolog | PWP2 | 0.42 |
| P21589-2;P21589;Q96B60;H0Y7R7;REV__Q9H2S1-2;REV__Q9H2S1 | P21589-2;P21589;Q96B60 | 5;5;3;1;1;1 | 5;5;3;1;1;1 | 5;5;3;1;1;1 | 5-nucleotidase | NT5E | 0.43 |
| P53004;C9J1E1 | P53004 | 15;4 | 15;4 | 15;4 | Biliverdin reductase A | BLVRA | 0.43 |
| P03956 | P03956 | 25 | 25 | 25 | Interstitial collagenase;22 kDa interstitial collagenase;27 kDa interstitial collagenase | MMP1 | 0.43 |
| Q5H909;Q9UNF1-2;Q9UNF1;Q5H907;A0A087X070;G5E9N2;Q12816-5;Q12816-2;Q12816-3;Q12816-4;Q12816 | Q5H909;Q9UNF1-2;Q9UNF1;Q5H907 | 17;17;17;15;2;2;2;2;2;2;2 | 17;17;17;15;2;2;2;2;2;2;2 | 16;16;16;14;2;2;2;2;2;2;2 | Melanoma-associated antigen D2 | MAGED2 | 0.43 |
| Q5T9L3;Q5T9L3-2;E9PM62;Q5T9L3-3;E9PKJ0;E9PJW4;H0YCG9;E9PQK1;Q7Z430;H0YDL2;E9PM23 | Q5T9L3;Q5T9L3-2;E9PM62;Q5T9L3-3;E9PKJ0;E9PJW4;H0YCG9;E9PQK1 | 8;8;7;6;5;5;5;4;3;2;1 | 8;8;7;6;5;5;5;4;3;2;1 | 8;8;7;6;5;5;5;4;3;2;1 | Protein wntless homolog | WLS | 0.43 |
| C9JWJ8;Q14938-6;Q14938-5;Q14938-3;K7EN08;Q14938-2;Q14938-4;Q14938;K7EKH0;K7ESG9;K7EJB0;K7EMQ5 | C9JWJ8;Q14938-6;Q14938-5;Q14938-3;K7EN08;Q14938-2;Q14938-4;Q14938;K7EKH0 | 8;8;8;8;7;7;7;7;4;3;3;2 | 7;7;7;7;6;6;6;6;3;2;2;2 | 5;5;5;5;4;4;4;4;1;0;0;0 | Nuclear factor 1;Nuclear factor 1 X-type | NFIX | 0.44 |
| Q8WUH6 | Q8WUH6 | 5 | 5 | 5 | Transmembrane protein 263 | TMEM263 | 0.44 |
| P35754 | P35754 | 5 | 5 | 5 | Glutaredoxin-1 | GLRX | 0.44 |
| O00151 | O00151 | 14 | 14 | 14 | PDZ and LIM domain protein 1 | PDLIM1 | 0.45 |
| Q03135;E9PCT5;C9JKI3;Q03135-2;P56539 | Q03135;E9PCT5;C9JKI3;Q03135-2 | 7;5;4;4;1 | 7;5;4;4;1 | 7;5;4;4;1 | Caveolin-1;Caveolin | CAV1 | 0.45 |
| Q16643;Q16643-2;Q16643-3;D6R9W4;D6RFI1;D6R9Q9;D6RCR4 | Q16643;Q16643-2;Q16643-3;D6R9W4 | 27;25;25;21;7;7;4 | 27;25;25;21;7;7;4 | 27;25;25;21;7;7;4 | Drebrin | DBN1 | 0.45 |
| C9J0J7;G5E9Q6;P35080-2;C9JQ45;C9J2N0 | C9J0J7;G5E9Q6;P35080-2 | 7;7;7;3;2 | 7;7;7;3;2 | 4;4;4;0;0 | Profilin;Profilin-2 | PFN2 | 0.45 |
| Q6NZI2;Q6NZI2-2;Q6NZI2-3 | Q6NZI2;Q6NZI2-2 | 16;9;6 | 16;9;6 | 16;9;6 | Polymerase I and transcript release factor | PTRF | 0.45 |
| Q9BTV5;M0R366;M0R2F6;M0R1R0 | Q9BTV5;M0R366 | 12;11;3;1 | 12;11;3;1 | 12;11;3;1 | Fibronectin type III and SPRY domain-containing protein 1 | FSD1 | 0.45 |
| A0A0C4DGH2;P62070;P62070-4;P62070-3;E9PK85;P62070-2;E9PQ87;E9PQK5;E9PQC5 | A0A0C4DGH2;P62070;P62070-4;P62070-3;E9PK85;P62070-2 | 8;8;8;7;6;6;3;1;1 | 8;8;8;7;6;6;3;1;1 | 6;6;6;6;5;6;3;0;0 | Ras-related protein R-Ras2 | RRAS2 | 0.46 |
| Q9Y281;Q9Y281-3;F8WDN3 | Q9Y281;Q9Y281-3 | 18;17;7 | 12;12;6 | 12;12;6 | Cofilin-2 | CFL2 | 0.46 |
| O75506 | O75506 | 5 | 5 | 5 | Heat shock factor-binding protein 1 | HSBP1 | 0.46 |
| P10301 | P10301 | 6 | 4 | 4 | Ras-related protein R-Ras | RRAS | 0.46 |
| Q12929;Q12929-2;F5H0R8;H0YFG1;F5H3Q6 | Q12929;Q12929-2 | 15;10;3;1;1 | 15;10;3;1;1 | 15;10;3;1;1 | Epidermal growth factor receptor kinase substrate 8 | EPS8 | 0.46 |
| P09104;P09104-2;F5H0C8;F5H1C3;U3KQP4;U3KQQ1 | P09104;P09104-2;F5H0C8 | 20;16;15;5;2;1 | 15;12;12;4;1;1 | 13;10;10;4;1;0 | Gamma-enolase;Enolase | ENO2 | 0.46 |
| F5H2F4;V9GY75 | F5H2F4 | 61;1 | 1;1 | 1;1 |  | MTHFD1 | 0.46 |
| P80723;P80723-2;U3KQP0 | P80723;P80723-2 | 22;13;3 | 22;13;3 | 22;13;3 | Brain acid soluble protein 1 | BASP1 | 0.47 |
| C9IZC9;H7BZ75;O75600;O75600-2;F2Z340 | C9IZC9;H7BZ75;O75600;O75600-2;F2Z340 | 2;2;2;2;1 | 2;2;2;2;1 | 2;2;2;2;1 | 2-amino-3-ketobutyrate coenzyme A ligase, mitochondrial | GCAT | 0.47 |
| P30479 | P30479 | 13 | 8 | 0 | HLA class I histocompatibility antigen, B-41 alpha chain | HLA-B | 0.47 |
| P35520;P35520-2;C9JMA6;H7C2H4;H7C1W6;H7C2W0 | P35520;P35520-2 | 12;12;4;4;1;1 | 12;12;4;4;1;1 | 12;12;4;4;1;1 | Cystathionine beta-synthase | CBS | 0.47 |
| F6Y5H0;P29558-2;P29558;E7ETU5;E7EPF2;C9J9B2;C9JIJ9;Q6XE24-3;Q6XE24-4;Q6XE24-5;Q6XE24-2;Q6XE24;F8VV01 | F6Y5H0;P29558-2;P29558;E7ETU5 | 10;10;10;9;4;2;2;2;2;2;2;2;1 | 10;10;10;9;4;2;2;2;2;2;2;2;1 | 7;7;7;6;2;1;1;1;1;1;1;1;0 | RNA-binding motif, single-stranded-interacting protein 1 | RBMS1 | 0.47 |
| P46821;D6RA32;D6RA40;E9PGC8;P78559;P78559-2;D6RCL2;D6RGJ3;C9JKF1;Q8IVG5 | P46821 | 77;22;20;2;2;2;1;1;1;1 | 77;22;20;2;2;2;1;1;1;1 | 76;21;19;2;2;2;1;1;0;0 | Microtubule-associated protein 1B;MAP1B heavy chain;MAP1 light chain LC1 | MAP1B | 0.47 |
| Q92466;Q92466-4;A0A087WYT8;A0A087X0X5;Q92466-2;Q92466-5;A0A087WW71;Q92466-3 | Q92466;Q92466-4 | 7;4;3;3;3;3;2;2 | 7;4;3;3;3;3;2;2 | 7;4;3;3;3;3;2;2 | DNA damage-binding protein 2 | DDB2 | 0.48 |
| O60504-2;O60504;H0YB51;E5RHI2;H0YBA2;E5RJP2;H0YAZ3;E5RIA0;E5RH58 | O60504-2;O60504;H0YB51;E5RHI2;H0YBA2;E5RJP2;H0YAZ3 | 7;7;5;5;5;4;4;2;2 | 7;7;5;5;5;4;4;2;2 | 7;7;5;5;5;4;4;2;2 | Vinexin | SORBS3 | 0.48 |
| Q96PY5;Q96PY5-3;C9IZY8 | Q96PY5;Q96PY5-3;C9IZY8 | 16;16;11 | 16;16;11 | 13;13;8 | Formin-like protein 2 | FMNL2 | 0.48 |
| P42025 | P42025 | 18 | 6 | 6 | Beta-centractin | ACTR1B | 0.48 |
| Q93052;A0A087WZF1;C9JUT4;C9JXK9;C9JIY7;C9JT42;C9J3U9;C9J1K7;C9J5C8;C9J4E3;C9JE51;C9J2R5 | Q93052;A0A087WZF1;C9JUT4 | 22;21;13;9;4;4;4;4;4;4;2;2 | 22;21;13;9;4;4;4;4;4;4;2;2 | 22;21;13;9;4;4;4;4;4;4;2;2 | Lipoma-preferred partner | LPP | 0.48 |
| P12277;H0YJG0;G3V4N7;G3V461;H0YJK0;P06732;G3V2I1 | P12277;H0YJG0 | 25;14;12;5;4;3;1 | 25;14;12;5;4;3;1 | 24;13;11;5;4;2;1 | Creatine kinase B-type | CKB | 0.48 |
| Q03001;F8W9J4;Q03001-9 | Q03001;F8W9J4 | 47;44;13 | 46;43;13 | 5;5;5 | Dystonin | DST | 0.48 |
| O15056-2;O15056;O15056-3;E7ER60;X6R647;H0YEV8 | O15056-2;O15056;O15056-3;E7ER60 | 13;13;11;9;4;2 | 13;13;11;9;4;2 | 13;13;11;9;4;2 | Synaptojanin-2 | SYNJ2 | 0.49 |
| Q63ZY3-3;Q63ZY3;Q63ZY3-2;K7ERU2;K7EIU4;K7ES05 | Q63ZY3-3;Q63ZY3;Q63ZY3-2 | 22;22;21;5;4;1 | 22;22;21;5;4;1 | 22;22;21;5;4;1 | KN motif and ankyrin repeat domain-containing protein 2 | KANK2 | 0.49 |
| P49419-2;P49419;F8VS02;P49419-4;P49419-3;H0YHM6;F8VVF2;F8WDY6;F8WD33 | P49419-2;P49419;F8VS02;P49419-4;P49419-3 | 27;27;20;20;18;8;4;1;1 | 27;27;20;20;18;8;4;1;1 | 27;27;20;20;18;8;4;1;1 | Alpha-aminoadipic semialdehyde dehydrogenase | ALDH7A1 | 0.49 |
| Q5RJ85;P17693 | Q5RJ85;P17693 | 1;1 | 1;1 | 1;1 | HLA class I histocompatibility antigen, alpha chain G | HLA-G | 0.50 |
| Q15417;Q15417-3;Q15417-2;E9PDU6;B7Z7E1;P51911-2;P51911;K7ESJ2;K7ENC5;K7EQ72;K7ERK4;K7ER02 | Q15417;Q15417-3;Q15417-2;E9PDU6 | 21;17;17;14;2;2;2;1;1;1;1;1 | 21;17;17;14;2;2;2;1;1;1;1;1 | 19;15;16;13;1;1;1;1;1;0;1;0 | Calponin-3;Calponin | CNN3 | 0.50 |
| P20700;E9PBF6 | P20700;E9PBF6 | 45;38 | 39;33 | 39;33 | Lamin-B1 | LMNB1 | 0.50 |
| E9PMS6;F8WD26;J3KP06;Q8WWI1-3;Q8WWI1;Q8WWI1-4;Q8WWI1-2;E9PMP7;E9PMT2;A0A0A0MTE2;Q8WWI1-5;H0Y424;H0YDG6;E9PJ10;E9PK58;H0YDQ3;E9PLU6;H0YE95;U3KQE6;E9PRE3;E9PRJ0 | E9PMS6;F8WD26;J3KP06;Q8WWI1-3;Q8WWI1;Q8WWI1-4;Q8WWI1-2;E9PMP7;E9PMT2;A0A0A0MTE2;Q8WWI1-5;H0Y424 | 24;24;24;24;24;22;22;19;19;17;17;14;5;4;3;3;2;2;1;1;1 | 24;24;24;24;24;22;22;19;19;17;17;14;5;4;3;3;2;2;1;1;1 | 24;24;24;24;24;22;22;19;19;17;17;14;5;4;3;3;2;2;1;1;1 | LIM domain only protein 7 | LMO7 | 0.50 |
| Q7Z5L9-2;Q7Z5L9;Q7Z5L9-3 | Q7Z5L9-2;Q7Z5L9 | 18;18;5 | 18;18;5 | 14;14;3 | Interferon regulatory factor 2-binding protein 2 | IRF2BP2 | 0.50 |
| O43294-2;O43294;H3BQC4;H3BSN4;H3BS04;H3BN49 | O43294-2;O43294 | 13;13;5;4;2;2 | 13;13;5;4;2;2 | 13;13;5;4;2;2 | Transforming growth factor beta-1-induced transcript 1 protein | TGFB1I1 | 0.50 |
| P60903 | P60903 | 7 | 7 | 7 | Protein S100-A10 | S100A10 | 0.50 |
| P00533;E9PFD7;Q504U8;A0A0B4J1Y5;P00533-4;P00533-3;P00533-2;C9JYS6;J3KTI5;H3BLT0;B4DTR1;J3QLU9;A0A0A0MSE1;P04626-3;P04626-2;P04626-6;P04626-5;P04626-4;P04626;Q15303-4;Q15303-3;Q15303-2;Q15303 | P00533;E9PFD7;Q504U8;A0A0B4J1Y5;P00533-4;P00533-3;P00533-2 | 25;23;19;16;16;16;13;4;1;1;1;1;1;1;1;1;1;1;1;1;1;1;1 | 25;23;19;16;16;16;13;4;1;1;1;1;1;1;1;1;1;1;1;1;1;1;1 | 25;23;19;16;16;16;13;4;1;1;1;1;1;1;1;1;1;1;1;1;1;1;1 | Epidermal growth factor receptor;Receptor protein-tyrosine kinase | EGFR | 0.50 |
| Q9UDT6-2;Q9UDT6 | Q9UDT6-2;Q9UDT6 | 13;12 | 7;6 | 7;6 | CAP-Gly domain-containing linker protein 2 | CLIP2 | 0.51 |
| Q8NFV4;Q8NFV4-4;Q8NFV4-6;C9J7Q4;H7BZ58;H0YC52;H7C396;Q8NFV4-3;Q8NFV4-2;Q8NFV4-5 | Q8NFV4;Q8NFV4-4;Q8NFV4-6 | 13;12;8;5;5;4;4;4;3;3 | 13;12;8;5;5;4;4;4;3;3 | 13;12;8;5;5;4;4;4;3;3 | Alpha/beta hydrolase domain-containing protein 11 | ABHD11 | 0.51 |
| A0A087WUU9;P47974;G3V2D5;G3V2P5;Q07352;M0QZ04;M0QY76;M0R0H3;P26651 | A0A087WUU9;P47974 | 5;5;2;2;2;1;1;1;1 | 5;5;2;2;2;1;1;1;1 | 5;5;2;2;2;1;1;1;1 |  | ZFP36L2 | 0.51 |
| P30043;M0R192;M0QZL1 | P30043;M0R192;M0QZL1 | 13;10;8 | 13;10;8 | 13;10;8 | Flavin reductase (NADPH) | BLVRB | 0.51 |
| F5H6E2;O00159-3;O00159;O00159-2;I3L204;I3L501;I3L168;I3L3F5;I3L4D4;I3L3Y6 | F5H6E2;O00159-3;O00159;O00159-2 | 41;41;41;40;8;4;3;3;3;3 | 41;41;41;40;8;4;3;3;3;3 | 40;40;40;39;8;4;3;3;3;3 | Unconventional myosin-Ic | MYO1C | 0.51 |
| Q8NFW8;Q8NFW8-2;F5GYM0;F5H296 | Q8NFW8;Q8NFW8-2 | 13;7;3;2 | 13;7;3;2 | 13;7;3;2 | N-acylneuraminate cytidylyltransferase | CMAS | 0.51 |
| Q92575;F8WB86;Q6PJ80;C9JLR4 | Q92575 | 8;1;1;1 | 8;1;1;1 | 8;1;1;1 | UBX domain-containing protein 4 | UBXN4 | 0.51 |
| Q9H3Q1;Q9H3Q1-2;J3KRZ9;J3QQS6;J3QR93 | Q9H3Q1;Q9H3Q1-2 | 9;8;3;2;1 | 9;8;3;2;1 | 9;8;3;2;1 | Cdc42 effector protein 4 | CDC42EP4 | 0.51 |
| P20337;M0R257;Q96E17 | P20337 | 10;2;2 | 10;2;2 | 8;0;0 | Ras-related protein Rab-3B | RAB3B | 0.51 |
| E7ESP4;E7EMF1;E9PB77;D6RG08 | E7ESP4;E7EMF1;E9PB77 | 24;20;14;3 | 1;1;1;0 | 1;1;1;0 |  | ITGA2 | 0.51 |
| Q9NYL2;Q9NYL2-3;C9J3F7 | Q9NYL2;Q9NYL2-3 | 20;11;5 | 20;11;5 | 7;0;0 | Mitogen-activated protein kinase kinase kinase MLT | ZAK | 0.51 |
| Q15059;Q15059-2;Q58F21-5;Q58F21-4;Q58F21;Q58F21-3;Q58F21-2 | Q15059;Q15059-2 | 8;7;1;1;1;1;1 | 3;3;0;0;0;0;0 | 3;3;0;0;0;0;0 | Bromodomain-containing protein 3 | BRD3 | 0.52 |
| Q15149 | Q15149 | 386 | 386 | 6 | Plectin | PLEC | 0.52 |
| Q8WUP2;Q8WUP2-3;Q8WUP2-2;E7EWE8;D6RAI6;E7EPI5;E7EN81;D6R9I4;D6RA19 | Q8WUP2;Q8WUP2-3;Q8WUP2-2 | 8;7;5;3;2;2;2;1;1 | 8;7;5;3;2;2;2;1;1 | 8;7;5;3;2;2;2;1;1 | Filamin-binding LIM protein 1 | FBLIM1 | 0.52 |
| P30508;Q07000;Q29960-2;Q29960;Q29963;P30499 | P30508;Q07000;Q29960-2;Q29960;Q29963 | 13;12;10;10;10;5 | 8;8;5;5;6;4 | 0;0;0;0;0;0 | HLA class I histocompatibility antigen, Cw-12 alpha chain;HLA class I histocompatibility antigen, Cw-15 alpha chain;HLA class I histocompatibility antigen, Cw-16 alpha chain;HLA class I histocompatibility antigen, Cw-6 alpha chain | HLA-C | 0.52 |
| A6NGP5;Q9H910;Q9H910-3;H3BU16;Q9H910-2;H3BMV3;H3BMT0;H3BTV5;H3BMM8;B4E1P3 | A6NGP5;Q9H910;Q9H910-3;H3BU16;Q9H910-2;H3BMV3;H3BMT0;H3BTV5 | 9;9;9;8;8;7;6;6;2;2 | 9;9;9;8;8;7;6;6;2;2 | 9;9;9;8;8;7;6;6;2;2 | Hematological and neurological expressed 1-like protein | HN1L | 0.52 |
| F8VQR7;Q16527;F8VW96 | F8VQR7;Q16527;F8VW96 | 6;6;5 | 6;6;5 | 6;6;5 | Cysteine and glycine-rich protein 2 | CSRP2 | 0.52 |
| A1X283;G3V144;H0Y507;Q5TCZ1-3;Q5TCZ1;Q5TCZ1-2 | A1X283 | 16;7;2;2;2;1 | 16;7;2;2;2;1 | 16;7;2;2;2;1 | SH3 and PX domain-containing protein 2B | SH3PXD2B | 0.52 |
| P05534 | P05534 | 19 | 19 | 3 | HLA class I histocompatibility antigen, A-24 alpha chain | HLA-A | 0.52 |
| Q9Y4K1;Q9Y4K1-2;Q96QW7 | Q9Y4K1 | 17;5;4 | 17;5;4 | 17;5;4 | Absent in melanoma 1 protein | AIM1 | 0.52 |
| Q15293;Q15293-2;E9PP27 | Q15293;Q15293-2 | 23;21;4 | 23;21;4 | 23;21;4 | Reticulocalbin-1 | RCN1 | 0.52 |
| P07355;P07355-2;H0YN42;H0YM50;H0YMD0;H0YMU9;A6NMY6;H0YNP5;H0YKS4;H0YMM1;H0YKZ7;H0YLV6;H0YMT9;H0YKX9;H0YKL9;H0YMW4;H0YKV8;H0YMD9;H0YNB8;H0YKN4 | P07355;P07355-2;H0YN42;H0YM50;H0YMD0;H0YMU9;A6NMY6 | 41;40;29;28;27;27;26;19;18;13;11;11;11;11;10;10;8;7;5;5 | 41;40;29;28;27;27;26;19;18;13;11;11;11;11;10;10;8;7;5;5 | 23;22;11;10;11;11;14;3;11;11;11;11;11;11;10;10;8;7;5;5 | Annexin A2;Annexin;Putative annexin A2-like protein | ANXA2;ANXA2P2 | 0.52 |
| Q32MZ4-4 | Q32MZ4-4 | 10 | 3 | 3 | Leucine-rich repeat flightless-interacting protein 1 | LRRFIP1 | 0.53 |
| Q09666;E9PKR9;E9PLK4;E9PJZ0;E9PJC6;Q09666-2;E9PQE3 | Q09666 | 355;5;5;4;4;4;3 | 355;5;5;4;4;4;3 | 352;5;5;4;4;4;3 | Neuroblast differentiation-associated protein AHNAK | AHNAK | 0.53 |
| Q92522 | Q92522 | 11 | 11 | 11 | Histone H1x | H1FX | 0.53 |
| Q969G5;E9PIE3 | Q969G5;E9PIE3 | 11;9 | 11;9 | 11;9 | Protein kinase C delta-binding protein | PRKCDBP | 0.53 |
| P24844;P24844-2 | P24844;P24844-2 | 13;8 | 5;4 | 5;4 | Myosin regulatory light polypeptide 9 | MYL9 | 0.53 |
| P20810-7;P20810-6;A0A0C4DGB5;P20810-9;P20810-10;P20810;B7Z574;P20810-4;P20810-8;E7ES10;E9PDE4;P20810-5;P20810-2;E9PCH5;P20810-3;H0Y7F0;E7EQ12;E7EQA0;H0YA91;F8W7E0;E9PSG1;A0A0C4DGD1;H0Y944;D6RGF7;D6RBR1;E7EQK6;E7EN75;D6RC54;D6RAA8;D6RBZ8 | P20810-7;P20810-6;A0A0C4DGB5;P20810-9;P20810-10;P20810;B7Z574;P20810-4;P20810-8;E7ES10;E9PDE4;P20810-5;P20810-2;E9PCH5;P20810-3;H0Y7F0;E7EQ12;E7EQA0 | 40;40;39;39;39;37;36;36;36;35;34;34;32;31;29;25;23;22;14;13;11;10;9;5;4;4;4;3;2;1 | 40;40;39;39;39;37;36;36;36;35;34;34;32;31;29;25;23;22;14;13;11;10;9;5;4;4;4;3;2;1 | 6;6;5;5;5;3;2;2;2;3;2;5;3;2;2;2;2;2;3;3;4;2;4;4;3;0;4;0;0;1 | Calpastatin | CAST | 0.54 |
| P49757-4;P49757-2;P49757-3;P49757;P49757-8;P49757-7;P49757-6;P49757-5;G3V3R1;G3V3M5;G3V3Z8;G3V4S6;G3V433;P49757-9 | P49757-4;P49757-2;P49757-3;P49757;P49757-8;P49757-7;P49757-6;P49757-5;G3V3R1;G3V3M5;G3V3Z8 | 10;10;10;10;9;9;9;9;7;6;5;2;2;2 | 10;10;10;10;9;9;9;9;7;6;5;2;2;2 | 7;7;7;7;6;6;6;6;4;3;4;2;2;2 | Protein numb homolog | NUMB | 0.54 |
| P49418-2;P49418;H0Y7T8 | P49418-2;P49418 | 12;12;4 | 10;10;4 | 10;10;4 | Amphiphysin | AMPH | 0.54 |
| A0A0A0MRE5;Q9ULH1;Q9ULH1-2;H0YBF7;H0YBY9;E5RHD7;H0YBM4;E5RFD9 | A0A0A0MRE5;Q9ULH1;Q9ULH1-2;H0YBF7 | 14;14;14;10;4;3;3;1 | 14;14;14;10;4;3;3;1 | 13;13;13;9;3;3;3;1 | Arf-GAP with SH3 domain, ANK repeat and PH domain-containing protein 1 | ASAP1 | 0.54 |
| Q9UKS6;A0A0C4DGG1;E9PIY1;E9PJ75;E9PNM9;E9PIZ6;E9PJ33 | Q9UKS6;A0A0C4DGG1;E9PIY1 | 10;9;8;3;3;1;1 | 10;9;8;3;3;1;1 | 10;9;8;3;3;1;1 | Protein kinase C and casein kinase substrate in neurons protein 3 | PACSIN3 | 0.54 |
| O75131;A0A087WYQ3;H0YB26;E5RHZ0;A0A087WXR6;A0A087WUS8;E5RFT7;Q9HCH3-2;Q96FN4-2;Q8IYJ1;Q96A23;O95741;Q9UBL6-2;Q96A23-2;O95741-2;Q9UBL6 | O75131;A0A087WYQ3 | 22;12;10;8;7;6;2;1;1;1;1;1;1;1;1;1 | 22;12;10;8;7;6;2;1;1;1;1;1;1;1;1;1 | 21;12;10;8;7;6;2;0;0;0;0;0;0;0;0;0 | Copine-3 | CPNE3 | 0.54 |
| Q9UBI6 | Q9UBI6 | 5 | 5 | 5 | Guanine nucleotide-binding protein G(I)/G(S)/G(O) subunit gamma-12 | GNG12 | 0.54 |
| B1AKR6;Q9NP97;H3BQI1;Q9NP97-2;Q8TF09 | B1AKR6;Q9NP97 | 6;6;1;1;1 | 6;6;1;1;1 | 6;6;1;1;1 | Dynein light chain roadblock-type 1 | DYNLRB1 | 0.54 |
| Q6PI78 | Q6PI78 | 4 | 4 | 4 | Transmembrane protein 65 | TMEM65 | 0.54 |
| Q9Y508;A0A096LNV3;A0A096LP02;Q9Y508-2;A0A096LNT1;A0A096LNN8;A0A096LPF9 | Q9Y508;A0A096LNV3;A0A096LP02;Q9Y508-2 | 17;15;15;15;2;2;2 | 17;15;15;15;2;2;2 | 17;15;15;15;2;2;2 | E3 ubiquitin-protein ligase RNF114 | RNF114 | 0.54 |
| A0A096LP25;Q6ZSR9;A0A096LNZ0 | A0A096LP25;Q6ZSR9;A0A096LNZ0 | 8;6;5 | 7;6;4 | 7;6;4 | Uncharacterized protein FLJ45252 | AAK1 | 0.54 |
| P14209-3;P14209 | P14209-3;P14209 | 3;3 | 3;3 | 1;1 | CD99 antigen | CD99 | 0.55 |
| Q12965;H0YNQ8;O00160;H0YN00;H0YLJ4;H0YLE5;M0QXU2 | Q12965 | 25;9;4;3;3;2;1 | 25;9;4;3;3;2;1 | 25;9;4;3;3;2;1 | Unconventional myosin-Ie | MYO1E | 0.55 |
| P17931;G3V3R6 | P17931;G3V3R6 | 12;9 | 12;9 | 12;9 | Galectin-3;Galectin | LGALS3 | 0.55 |
| P10606 | P10606 | 6 | 6 | 6 | Cytochrome c oxidase subunit 5B, mitochondrial | COX5B | 0.55 |
| I3L1T3;Q9GZP9 | I3L1T3;Q9GZP9 | 2;2 | 2;2 | 2;2 | Derlin-2 | DERL2 | 0.55 |
| Q9Y3P9;Q9Y3P9-2;Q9Y3P9-3;Q9Y3P9-4;B5MCD9;B7ZAP0 | Q9Y3P9;Q9Y3P9-2 | 20;12;8;4;1;1 | 20;12;8;4;1;1 | 19;12;8;4;1;0 | Rab GTPase-activating protein 1 | RABGAP1 | 0.55 |
| Q9Y4P8-3;Q9Y4P8-2;Q9Y4P8-5;Q9Y4P8-6 | Q9Y4P8-3;Q9Y4P8-2;Q9Y4P8-5;Q9Y4P8-6 | 9;9;9;9 | 9;9;9;9 | 1;1;1;1 | WD repeat domain phosphoinositide-interacting protein 2 | WIPI2 | 0.55 |
| H0Y6C7;F5GZ09;Q9BW83-2;Q9BW83;B1AH56;B1AH58 | H0Y6C7;F5GZ09;Q9BW83-2;Q9BW83;B1AH56;B1AH58 | 4;4;4;4;2;2 | 4;4;4;4;2;2 | 4;4;4;4;2;2 | Intraflagellar transport protein 27 homolog | IFT27 | 0.55 |
| O00622;A0A087WVM3 | O00622;A0A087WVM3 | 13;12 | 13;12 | 13;12 | Protein CYR61 | CYR61 | 0.55 |
| Q9BQS8;Q9BQS8-4;Q9BQS8-3;Q9BQS8-2 | Q9BQS8;Q9BQS8-4 | 14;14;5;5 | 14;14;5;5 | 14;14;5;5 | FYVE and coiled-coil domain-containing protein 1 | FYCO1 | 0.55 |
| Q53T59;F6TR53;B5MC96;H7BZZ1;H7C0Y9;H7BZ19 | Q53T59;F6TR53;B5MC96 | 11;9;6;3;2;1 | 11;9;6;3;2;1 | 11;9;6;3;2;1 | HCLS1-binding protein 3 | HS1BP3 | 0.55 |
| P40121;P40121-2;E7ENU9;B8ZZL6;H7C0X8 | P40121;P40121-2;E7ENU9 | 14;12;10;6;4 | 14;12;10;6;4 | 14;12;10;6;4 | Macrophage-capping protein | CAPG | 0.56 |
| O95834;O95834-2;O95834-3;A0A0C4DGQ7;C9JRL6;K7EIK7;K7ELI8;K7EII6;K7EKG3 | O95834;O95834-2;O95834-3;A0A0C4DGQ7;C9JRL6;K7EIK7 | 4;4;4;3;3;3;1;1;1 | 4;4;4;3;3;3;1;1;1 | 4;4;4;3;3;3;1;1;1 | Echinoderm microtubule-associated protein-like 2 | EML2 | 0.56 |
| E7ENN3;Q8NF91;A0A0C4DG40;Q8NF91-4;Q8NF91-7;Q8NF91-2;Q8NF91-8;H0Y326;H0Y325;Q8NF91-11;A0A0C4DH48;Q8NF91-10;Q5JV20;F8WAI0;Q8NF91-12;Q8NF91-9;Q8NF91-3;H0YGD3;REV__Q9P0K7-4;A0A087X1H7;REV__Q9P0K7-3;H0YBY6;REV__Q9P0K7;REV__Q9P0K7-2;Q9P1A6-3;Q9P1A6-2;Q9P1A6;Q8NF91-6 | E7ENN3;Q8NF91;A0A0C4DG40;Q8NF91-4;Q8NF91-7 | 24;24;22;22;13;11;10;4;4;4;3;3;2;2;2;2;2;1;1;1;1;1;1;1;1;1;1;1 | 23;23;21;21;13;10;9;4;4;4;3;3;2;2;2;2;2;1;1;1;1;1;1;1;1;1;1;1 | 23;23;21;21;13;10;9;4;4;4;3;3;2;2;2;2;2;1;1;1;1;1;1;1;1;1;1;1 | Nesprin-1 | SYNE1 | 0.56 |
| Q99536;Q99536-3;Q99536-2;K7ESA3;K7ERT7;K7ENX2;K7EM19;K7EJM4;Q9NQW1-5;Q9NQW1 | Q99536;Q99536-3;Q99536-2 | 22;17;13;10;9;5;5;1;1;1 | 22;17;13;10;9;5;5;1;1;1 | 22;17;13;10;9;5;5;1;1;1 | Synaptic vesicle membrane protein VAT-1 homolog | VAT1 | 0.56 |
| Q9UEY8-2;A0A087WX08;Q9UEY8 | Q9UEY8-2;A0A087WX08;Q9UEY8 | 11;10;10 | 11;10;10 | 11;10;10 | Gamma-adducin | ADD3 | 0.56 |
| P35611;P35611-3;E7EV99;E7ENY0;P35611-2;P35611-6;P35611-4;H0Y9H2;P35611-5;A0A0A0MSR2;D6RF25;D6RAH3;D6RJE2;H0YG19;H0YFD8;P35612-2;P35612-8;P35612-9;P35612-4;P35612-3;P35612 | P35611;P35611-3;E7EV99;E7ENY0;P35611-2;P35611-6;P35611-4;H0Y9H2;P35611-5;A0A0A0MSR2 | 25;25;24;24;24;24;23;15;15;13;5;4;3;3;2;1;1;1;1;1;1 | 25;25;24;24;24;24;23;15;15;13;5;4;3;3;2;1;1;1;1;1;1 | 25;25;24;24;24;24;23;15;15;13;5;4;3;3;2;1;1;1;1;1;1 | Alpha-adducin | ADD1 | 0.56 |
| B7ZM87;A2RUF3;O75044;A0A075B7B5;Q5VZB4;P0DMP2;P0DJJ0;A0A075B743;E9PDX4;A0A087X0L1;A0A087WW56;A0A087X1G6;A0A075B7E9 | B7ZM87;A2RUF3;O75044;A0A075B7B5;Q5VZB4 | 18;18;18;15;9;7;7;4;2;2;2;1;1 | 15;15;15;12;8;5;5;2;2;2;2;1;1 | 15;15;15;12;8;5;5;2;2;2;2;1;1 | SLIT-ROBO Rho GTPase-activating protein 2 | SRGAP2 | 0.56 |
| Q12788;J3KNP2;A0A087WYP7 | Q12788;J3KNP2;A0A087WYP7 | 6;5;3 | 6;5;3 | 6;5;3 | Transducin beta-like protein 3 | TBL3 | 0.57 |
| P17252;J3KRN5;P05771;P05771-2;H3BV73;J3KN97;P05129-2;P05129 | P17252;J3KRN5 | 16;8;7;7;3;2;1;1 | 16;8;7;7;3;2;1;1 | 16;8;7;7;3;2;1;1 | Protein kinase C alpha type | PRKCA | 0.57 |
| A0A087WYU1;Q9Y5X1;A0A087WVE4;A0A087WZW2 | A0A087WYU1;Q9Y5X1 | 24;24;1;1 | 24;24;1;1 | 24;24;1;1 | Sorting nexin;Sorting nexin-9 | SNX9 | 0.58 |
| Q86YB8;Q5TAE8;Q5T1H5;Q86YB8-2 | Q86YB8;Q5TAE8 | 4;2;1;1 | 3;1;1;1 | 3;1;1;1 | ERO1-like protein beta | ERO1LB | 0.58 |
| H0YMV8;Q71UM5;C9JLI6;C9J1C5 | H0YMV8;Q71UM5;C9JLI6 | 5;5;4;1 | 2;2;2;0 | 2;2;2;0 | 40S ribosomal protein S27;40S ribosomal protein S27-like | RPS27L | 0.58 |
| Q12906-7;Q12906-4;Q12906-6;K7EKJ9;K7EQR9;K7ER69;K7EJ09;K7EM82;K7EKY0;K7ERM6;K7ENK6;K7EPG3;K7ELV3;K7EQ75 | Q12906-7;Q12906-4;Q12906-6 | 50;44;44;16;10;9;8;7;7;5;5;4;2;1 | 2;2;2;0;0;0;0;0;0;0;0;0;0;0 | 2;2;2;0;0;0;0;0;0;0;0;0;0;0 | Interleukin enhancer-binding factor 3 | ILF3 | 0.58 |
| Q9ULC3 | Q9ULC3 | 11 | 11 | 11 | Ras-related protein Rab-23 | RAB23 | 0.58 |
| E7EW69;B5ME97;Q9P0V9;Q9P0V9-2;E7EX04;Q9P0V9-3;C9JEW2;C9JNR7;F5H1F2;C9JEQ7;F5GYV2;F5H1W3;F8WC85 | E7EW69;B5ME97;Q9P0V9;Q9P0V9-2;E7EX04;Q9P0V9-3 | 25;25;25;25;24;22;3;3;3;2;1;1;1 | 22;22;22;22;21;19;3;3;3;2;1;1;1 | 21;21;21;21;20;18;3;3;3;2;1;1;1 | Septin-10 | SEPT10 | 0.58 |
| Q9NRV9;F5GWX2;H0YG71 | Q9NRV9 | 9;4;1 | 9;4;1 | 9;4;1 | Heme-binding protein 1 | HEBP1 | 0.58 |
| Q9BYK8;Q9BYK8-2;Q9BYK8-4 | Q9BYK8;Q9BYK8-2;Q9BYK8-4 | 5;4;4 | 5;4;4 | 5;4;4 | Helicase with zinc finger domain 2 | HELZ2 | 0.58 |
| Q86XZ4;F8VS10 | Q86XZ4 | 7;2 | 6;2 | 6;2 | Spermatogenesis-associated serine-rich protein 2 | SPATS2 | 0.58 |
| Q03154-4;Q03154;C9JMV9;Q03154-2;Q03154-3;C9JYZ0;F8WC59 | Q03154-4;Q03154;C9JMV9;Q03154-2;Q03154-3;C9JYZ0 | 10;10;9;9;9;6;3 | 10;10;9;9;9;6;3 | 10;10;9;9;9;6;3 | Aminoacylase-1 | ACY1;ABHD14A-ACY1 | 0.58 |
| Q5GLZ8-2;Q5GLZ8;Q5GLZ8-3;Q5GLZ8-6;H0Y6K7;D6RCL5;Q5GLZ8-4;Q5GLZ8-5 | Q5GLZ8-2;Q5GLZ8;Q5GLZ8-3;Q5GLZ8-6 | 39;39;38;35;13;8;3;3 | 39;39;38;35;13;8;3;3 | 39;39;38;35;13;8;3;3 | Probable E3 ubiquitin-protein ligase HERC4 | HERC4 | 0.58 |
| P50995-2;P50995;H0Y6E1;E5RIN3;P27216;P27216-2 | P50995-2;P50995 | 21;21;5;1;1;1 | 21;21;5;1;1;1 | 21;21;5;1;1;1 | Annexin A11 | ANXA11 | 0.58 |
| P08758;D6RBL5;D6RBE9;E9PHT9;D6RCN3 | P08758;D6RBL5;D6RBE9 | 36;26;24;17;5 | 36;26;24;17;5 | 36;26;24;17;5 | Annexin A5;Annexin | ANXA5 | 0.58 |
| P31937;H7BZL2 | P31937;H7BZL2 | 13;7 | 13;7 | 13;7 | 3-hydroxyisobutyrate dehydrogenase, mitochondrial | HIBADH | 0.59 |
| Q8TDW0;M0R3C1;M0QZ48;Q6NSJ5 | Q8TDW0 | 6;1;1;1 | 6;1;1;1 | 6;1;1;1 | Volume-regulated anion channel subunit LRRC8C | LRRC8C | 0.59 |
| Q16513-3;Q16513;Q16513-2;Q16513-4;Q16513-5;B1AL79;A0A0A0MRN8;H0Y429;H0Y5V5;Q6P5Z2 | Q16513-3;Q16513;Q16513-2;Q16513-4;Q16513-5 | 28;28;27;25;21;12;8;3;2;1 | 28;28;27;25;21;12;8;3;2;1 | 26;26;25;23;19;12;8;1;2;0 | Serine/threonine-protein kinase N2 | PKN2 | 0.59 |
| C9JGI3;P19971;P19971-2 | C9JGI3;P19971;P19971-2 | 12;12;10 | 12;12;10 | 12;12;10 | Thymidine phosphorylase | TYMP | 0.59 |
| O60664-4;O60664;O60664-3;K7ERZ3;O60664-2;K7EL96;K7ER39 | O60664-4;O60664;O60664-3;K7ERZ3;O60664-2 | 28;28;26;18;18;9;6 | 28;28;26;18;18;9;6 | 28;28;26;18;18;9;6 | Perilipin-3 | PLIN3 | 0.59 |
| Q9UHL4;R4GMR2;R4GMU5;R4GMV4;R4GNE8 | Q9UHL4 | 7;2;1;1;1 | 7;2;1;1;1 | 7;2;1;1;1 | Dipeptidyl peptidase 2 | DPP7 | 0.60 |
| G5E9A6;P51784;Q5JXD3 | G5E9A6;P51784 | 18;18;2 | 17;17;2 | 16;16;2 | Ubiquitin carboxyl-terminal hydrolase;Ubiquitin carboxyl-terminal hydrolase 11 | USP11 | 0.60 |
| E7EVA0;P27816;P27816-6;P27816-2;P27816-4;B5MEG9;H0Y2V1;F8W9U4 | E7EVA0;P27816;P27816-6;P27816-2 | 67;63;61;51;32;25;19;16 | 67;63;61;51;32;25;19;16 | 29;25;24;17;7;3;3;3 | Microtubule-associated protein;Microtubule-associated protein 4 | MAP4 | 0.60 |
| Q14195-2;Q14195;H0YBT4;D6RF19;H0YB87;Q14117 | Q14195-2;Q14195 | 36;28;9;3;1;1 | 36;28;9;3;1;1 | 31;23;5;2;1;0 | Dihydropyrimidinase-related protein 3 | DPYSL3 | 0.60 |
| O94855;O94855-2;E9PDM8;E9PC44 | O94855;O94855-2;E9PDM8;E9PC44 | 22;22;17;11 | 20;20;17;11 | 20;20;17;11 | Protein transport protein Sec24D | SEC24D | 0.60 |
| O14907;A0A087X282 | O14907 | 3;1 | 3;1 | 3;1 | Tax1-binding protein 3 | TAX1BP3 | 0.60 |
| Q12765;Q12765-2;Q12765-3;C9K052;B8ZZP4;C9J7U9 | Q12765;Q12765-2;Q12765-3 | 21;21;17;8;5;5 | 21;21;17;8;5;5 | 20;20;16;7;5;5 | Secernin-1 | SCRN1 | 0.60 |
| E7EX44;Q05682-5;Q05682-3;Q05682-4;Q05682-6;Q05682-2;Q05682;E9PGZ1;C9J813;C9JEK3;C9JE79;F8WE61 | E7EX44;Q05682-5;Q05682-3;Q05682-4;Q05682-6;Q05682-2;Q05682;E9PGZ1;C9J813 | 27;27;27;26;26;26;26;25;22;4;1;1 | 27;27;27;26;26;26;26;25;22;4;1;1 | 27;27;27;26;26;26;26;25;22;4;1;1 | Caldesmon | CALD1 | 0.61 |
| A0A024R4E5;Q00341;Q00341-2;H0Y394;H7C0A4;H7C2D1;C9JIZ1;C9J5E5;H7BZC3;C9JZI8;C9JHZ8;C9JES8;C9JHS7;C9JT62;C9JK79;C9JBS3;C9JEJ8;C9JHN6;C9JQ82;C9JMQ6;H7C3D0 | A0A024R4E5;Q00341;Q00341-2;H0Y394 | 65;64;61;49;19;18;13;12;12;10;9;9;8;5;5;4;4;4;3;2;1 | 65;64;61;49;19;18;13;12;12;10;9;9;8;5;5;4;4;4;3;2;1 | 65;64;61;49;19;18;13;12;12;10;9;9;8;5;5;4;4;4;3;2;1 | Vigilin | HDLBP | 0.61 |
| O60565;O60565-2;Q9H772 | O60565;O60565-2 | 9;7;1 | 9;7;1 | 9;7;1 | Gremlin-1 | GREM1 | 0.61 |
| Q13557-12;D6R938;Q13557-8;Q13557-10;Q13557;Q13557-6;Q13557-11;E9PBG7;E9PF82;Q13557-9;Q13557-5;Q13557-3;Q13557-4;H0Y9J2;H0Y9C2;Q9UQM7;Q9UQM7-2;D6RHX9;E9PBE8;E7EQE4 | Q13557-12;D6R938;Q13557-8;Q13557-10;Q13557;Q13557-6;Q13557-11;E9PBG7;E9PF82;Q13557-9;Q13557-5;Q13557-3;Q13557-4 | 18;17;17;17;17;17;17;16;16;16;16;16;16;8;8;3;3;1;1;1 | 18;17;17;17;17;17;17;16;16;16;16;16;16;8;8;3;3;1;1;1 | 14;13;13;13;13;13;13;12;12;12;12;12;12;7;5;0;0;0;0;0 | Calcium/calmodulin-dependent protein kinase type II subunit delta | CAMK2D | 0.61 |
| Q6SZW1;Q6SZW1-2;J3QRE0;J3KSG7 | Q6SZW1;Q6SZW1-2 | 9;8;2;2 | 9;8;2;2 | 9;8;2;2 | Sterile alpha and TIR motif-containing protein 1 | SARM1 | 0.61 |
| Q5SW79;Q5SW79-3;H0Y2V6;Q5SW79-2;E7EMW0;E7EWM2;H0Y4T4;Q96L14;E5RG47;H0YB92;E5RJT5;E5RFU8;E5RIH6;E5RGW7;H0YB66;J3KQR7;Q9Y4F5-3;Q9Y4F5-2;Q9Y4F5 | Q5SW79;Q5SW79-3;H0Y2V6;Q5SW79-2 | 66;63;61;61;12;11;10;10;7;6;5;5;4;4;2;2;2;2;2 | 66;63;61;61;12;11;10;10;7;6;5;5;4;4;2;2;2;2;2 | 66;63;61;61;12;11;10;10;7;6;5;5;4;4;2;2;2;2;2 | Centrosomal protein of 170 kDa | CEP170 | 0.61 |
| P98170;B1AKU2 | P98170;B1AKU2 | 6;3 | 6;3 | 6;3 | E3 ubiquitin-protein ligase XIAP | XIAP | 0.61 |
| P08237;P08237-3;P08237-2;F8VX13;F8VNX2;F8VZQ1;F8VSL1;F8VP00;F8W1J8;F8VSF7;F8VW30;F8VUB8;F8VYK8;F8VTQ3;F8VZI0;H0YHB8;H0YIS9;REV__Q86XR5-2;REV__Q86XR5;F8VVE3;REV__H7C487 | P08237;P08237-3;P08237-2 | 28;28;26;5;5;5;4;4;2;2;2;2;2;2;2;1;1;1;1;1;1 | 24;24;24;5;5;5;4;4;2;2;2;2;2;2;2;1;1;1;1;1;1 | 24;24;24;5;5;5;4;4;2;2;2;2;2;2;2;1;1;1;1;1;1 | ATP-dependent 6-phosphofructokinase, muscle type | PFKM | 0.62 |
| O95394;O95394-3;O95394-4;A0A087WT27;J3KN95;D6RCQ8;D6RF77;D6RC77;D6RCD1;D6RIS6;H0Y8I3;H0Y987 | O95394;O95394-3;O95394-4;A0A087WT27;J3KN95 | 22;22;22;14;14;9;9;7;6;5;3;3 | 22;22;22;14;14;9;9;7;6;5;3;3 | 22;22;22;14;14;9;9;7;6;5;3;3 | Phosphoacetylglucosamine mutase | PGM3 | 0.62 |
| F2Z2E2;Q86VI3 | F2Z2E2;Q86VI3 | 22;22 | 19;19 | 19;19 | Ras GTPase-activating-like protein IQGAP3 | IQGAP3 | 0.62 |
| F8W930;Q9Y6M1-1;Q9Y6M1;Q9Y6M1-5;Q9Y6M1-6;Q9Y6M1-3;Q9Y6M1-4 | F8W930;Q9Y6M1-1;Q9Y6M1;Q9Y6M1-5;Q9Y6M1-6;Q9Y6M1-3;Q9Y6M1-4 | 27;27;27;21;21;21;21 | 27;27;27;21;21;21;21 | 26;26;26;20;20;20;20 | Insulin-like growth factor 2 mRNA-binding protein 2 | IGF2BP2 | 0.62 |
| Q8TDQ7-3;Q8TDQ7;Q8TDQ7-2;Q8TDQ7-5;Q8TDQ7-4 | Q8TDQ7-3;Q8TDQ7;Q8TDQ7-2;Q8TDQ7-5;Q8TDQ7-4 | 11;11;10;8;7 | 5;5;5;5;4 | 5;5;5;5;4 | Glucosamine-6-phosphate isomerase 2 | GNPDA2 | 0.63 |
| Q86W92-4;Q86W92;Q86W92-3;F5GZP6;H0YFE4;A0A0A0MTP2;H0YGH8;F5H6Q7;F5H0E0;F5H495;E9PP16;Q86W92-5;Q8ND30-3;Q8ND30-2;Q8ND30 | Q86W92-4;Q86W92;Q86W92-3;F5GZP6 | 35;34;32;26;10;7;3;2;2;2;2;2;2;2;2 | 35;34;32;26;10;7;3;2;2;2;2;2;2;2;2 | 2;2;2;2;0;0;0;0;0;0;0;0;0;0;0 | Liprin-beta-1 | PPFIBP1 | 0.63 |
| Q8IVM0;Q8IVM0-2 | Q8IVM0;Q8IVM0-2 | 7;6 | 7;6 | 7;6 | Coiled-coil domain-containing protein 50 | CCDC50 | 0.63 |
| Q14789;Q14789-2;Q14789-4;Q14789-3;E7EU81;H0Y867;F8WF12;C9J8Q0;H7C5I7;REV__Q9UEE9-2;REV__O43688-3;REV__O43688;REV__Q9UEE9;REV__O43688-2 | Q14789;Q14789-2;Q14789-4;Q14789-3;E7EU81 | 56;56;55;55;32;22;1;1;1;1;1;1;1;1 | 56;56;55;55;32;22;1;1;1;1;1;1;1;1 | 54;54;53;53;31;22;1;1;1;0;0;0;0;0 | Golgin subfamily B member 1 | GOLGB1 | 0.64 |
| Q07812-5;Q07812-8;Q07812;Q07812-2;Q07812-7;K4JQN1;I6LPK7;Q07812-6;Q07812-4 | Q07812-5;Q07812-8;Q07812;Q07812-2;Q07812-7 | 7;7;7;7;6;3;1;1;1 | 7;7;7;7;6;3;1;1;1 | 7;7;7;7;6;3;1;1;1 | Apoptosis regulator BAX | BAX | 0.64 |
| Q9NQ48-2;Q9NQ48;H7C488;Q9NQ48-3 | Q9NQ48-2;Q9NQ48;H7C488;Q9NQ48-3 | 7;7;6;6 | 7;7;6;6 | 7;7;6;6 | Leucine zipper transcription factor-like protein 1 | LZTFL1 | 0.64 |
| Q9NUY8-2;Q9NUY8;C9IZ32;C9JAM5;E9PGE5 | Q9NUY8-2;Q9NUY8;C9IZ32;C9JAM5;E9PGE5 | 13;13;7;7;7 | 13;13;7;7;7 | 13;13;7;7;7 | TBC1 domain family member 23 | TBC1D23 | 0.64 |
| Q9H3P7 | Q9H3P7 | 16 | 16 | 16 | Golgi resident protein GCP60 | ACBD3 | 0.65 |
| Q15843 | Q15843 | 4 | 4 | 1 | NEDD8 | NEDD8 | 0.65 |
| E9PGC0;P20936-2;P20936-4;P20936;P20936-3 | E9PGC0;P20936-2;P20936-4;P20936 | 23;23;23;23;9 | 23;23;23;23;9 | 23;23;23;23;9 | Ras GTPase-activating protein 1 | RASA1 | 0.65 |
| Q01970-2;Q01970 | Q01970-2;Q01970 | 18;18 | 18;18 | 18;18 | 1-phosphatidylinositol 4,5-bisphosphate phosphodiesterase beta-3 | PLCB3 | 0.66 |
| C9JRJ5;Q9UGP4 | C9JRJ5;Q9UGP4 | 8;8 | 8;8 | 8;8 | LIM domain-containing protein 1 | LIMD1 | 0.66 |
| Q14247;Q14247-3;Q14247-2;H7C314;H0YEV2;H0YCD9;E9PKG3;E9PP90 | Q14247;Q14247-3;Q14247-2 | 26;25;24;11;6;6;2;2 | 26;25;24;11;6;6;2;2 | 26;25;24;11;6;6;2;2 | Src substrate cortactin | CTTN | 0.67 |
| F5H8F7;Q9UBL3-3;Q9UBL3;Q9UBL3-2;H0YBF6;H0YAQ0 | F5H8F7;Q9UBL3-3;Q9UBL3;Q9UBL3-2 | 11;11;11;9;4;2 | 11;11;11;9;4;2 | 11;11;11;9;4;2 | Set1/Ash2 histone methyltransferase complex subunit ASH2 | ASH2L | 1.57 |
| P42704;A0A0C4DG06;B8ZZ38;C9JCA9;H7C3W8;A0A0C4DG51;Q9NP80-3;Q9NP80-2;Q9NP80 | P42704 | 90;31;30;24;3;1;1;1;1 | 90;31;30;24;3;1;1;1;1 | 90;31;30;24;3;1;1;1;1 | Leucine-rich PPR motif-containing protein, mitochondrial | LRPPRC | 1.60 |
| Q15067-2;Q15067;Q15067-3;K7ELT1;I3L0T4;I3L2U4;K7ENF1;K7ESC7 | Q15067-2;Q15067;Q15067-3 | 9;8;7;2;2;1;1;1 | 9;8;7;2;2;1;1;1 | 9;8;7;2;2;1;1;1 | Peroxisomal acyl-coenzyme A oxidase 1 | ACOX1 | 1.60 |
| O94776;O94776-2 | O94776;O94776-2 | 29;18 | 29;18 | 24;15 | Metastasis-associated protein MTA2 | MTA2 | 1.60 |
| O14828;O14828-2 | O14828;O14828-2 | 5;4 | 5;4 | 5;4 | Secretory carrier-associated membrane protein 3 | SCAMP3 | 1.60 |
| P31689;P31689-2 | P31689;P31689-2 | 21;17 | 20;17 | 20;17 | DnaJ homolog subfamily A member 1 | DNAJA1 | 1.62 |
| Q96TA2-3;Q96TA2-2;Q96TA2;Q96I63;R4GNA5;Q5T8D1;Q5T8D2 | Q96TA2-3;Q96TA2-2;Q96TA2;Q96I63 | 11;11;11;10;5;4;3 | 11;11;11;10;5;4;3 | 11;11;11;10;5;4;3 | ATP-dependent zinc metalloprotease YME1L1 | YME1L1 | 1.62 |
| O15160;E7EQB9;O15160-2;D6RDJ3;H0Y723 | O15160;E7EQB9;O15160-2;D6RDJ3;H0Y723 | 7;6;6;4;4 | 7;6;6;4;4 | 7;6;6;4;4 | DNA-directed RNA polymerases I and III subunit RPAC1 | POLR1C | 1.63 |
| E7EQZ4;Q16637-3;Q16637;B4DP61;Q16637-4;Q16637-2;H0YBZ9 | E7EQZ4;Q16637-3;Q16637;B4DP61;Q16637-4;Q16637-2 | 6;6;6;5;5;5;1 | 6;6;6;5;5;5;1 | 6;6;6;5;5;5;1 | Survival motor neuron protein | SMN1;SMN2 | 1.65 |
| P12236;Q9H0C2 | P12236 | 21;3 | 21;3 | 5;0 | ADP/ATP translocase 3;ADP/ATP translocase 3, N-terminally processed | SLC25A6 | 1.65 |
| Q9NRN7;E9PLW6;E9PNF3;Q9NRN7-2 | Q9NRN7;E9PLW6 | 11;7;4;4 | 11;7;4;4 | 11;7;4;4 | L-aminoadipate-semialdehyde dehydrogenase-phosphopantetheinyl transferase | AASDHPPT | 1.65 |
| C9JA08;Q96D46;C9J0B9;C9IZW9;C9K0C2;C9IY70 | C9JA08;Q96D46 | 11;11;5;4;4;3 | 11;11;5;4;4;3 | 11;11;5;4;4;3 | 60S ribosomal export protein NMD3 | NMD3 | 1.70 |
| P45880;A0A0A0MR02;P45880-2;P45880-1;Q5JSD2;Q5JSD1;A2A3S1 | P45880;A0A0A0MR02;P45880-2;P45880-1;Q5JSD2;Q5JSD1 | 13;12;12;12;9;9;4 | 13;12;12;12;9;9;4 | 13;12;12;12;9;9;4 | Voltage-dependent anion-selective channel protein 2 | VDAC2 | 1.72 |
| Q16822;B4DW73;H0YM31;H0YML5;A0A0A0MS74;Q16822-2;H0YMA5;P35558;H0YNH9;H0YKC4;H0YMU6;H0YMY3;H0YNG4;P35558-2 | Q16822;B4DW73;H0YM31;H0YML5;A0A0A0MS74;Q16822-2 | 22;15;14;13;12;12;2;2;1;1;1;1;1;1 | 22;15;14;13;12;12;2;2;1;1;1;1;1;1 | 22;15;14;13;12;12;2;2;1;1;1;1;1;1 | Phosphoenolpyruvate carboxykinase [GTP], mitochondrial | PCK2 | 1.74 |
| Q9UKD2 | Q9UKD2 | 14 | 14 | 14 | mRNA turnover protein 4 homolog | MRTO4 | 1.74 |
| Q99714;Q99714-2;Q5H928 | Q99714;Q99714-2;Q5H928 | 14;12;9 | 14;12;9 | 14;12;9 | 3-hydroxyacyl-CoA dehydrogenase type-2 | HSD17B10 | 1.74 |
| Q8N806;E9PCJ7;H0YJM2;H0YJY4;H0YJA0;G3V3Q6;G3V336;G3V2G3 | Q8N806;E9PCJ7;H0YJM2 | 9;6;5;4;3;2;1;1 | 9;6;5;4;3;2;1;1 | 9;6;5;4;3;2;1;1 | Putative E3 ubiquitin-protein ligase UBR7 | UBR7 | 1.75 |
| Q9H3N1;G3V448 | Q9H3N1 | 9;2 | 9;2 | 9;2 | Thioredoxin-related transmembrane protein 1 | TMX1 | 1.75 |
| Q8N684-2;Q8N684;Q8N684-3;F5H669;F5H6M0;F5H047;J3QT54;C9J286;C9J323 | Q8N684-2;Q8N684;Q8N684-3;F5H669 | 13;13;13;11;4;4;3;1;1 | 13;13;13;11;4;4;3;1;1 | 13;13;13;11;4;4;3;1;1 | Cleavage and polyadenylation specificity factor subunit 7 | CPSF7 | 1.76 |
| E9PMJ2;J3KP39;Q9BPY3;E9PMW3;E9PIM5;G3V179 | E9PMJ2;J3KP39;Q9BPY3;E9PMW3;E9PIM5;G3V179 | 3;3;3;2;2;2 | 3;3;3;2;2;2 | 3;3;3;2;2;2 | Protein FAM118B | FAM118B | 1.77 |
| A0A087WU53;Q9H0U3;Q9H0U3-2 | A0A087WU53;Q9H0U3;Q9H0U3-2 | 7;7;4 | 7;7;4 | 7;7;4 | Magnesium transporter protein 1 | MAGT1 | 1.77 |
| Q7L0Y3;C9JVB6 | Q7L0Y3;C9JVB6 | 18;12 | 18;12 | 18;12 | Mitochondrial ribonuclease P protein 1 | TRMT10C | 1.78 |
| Q969U7-2;Q969U7;K7ENR6;V9GZ55 | Q969U7-2;Q969U7;K7ENR6;V9GZ55 | 8;8;5;4 | 8;8;5;4 | 8;8;5;4 | Proteasome assembly chaperone 2 | PSMG2 | 1.79 |
| O60547-2;O60547 | O60547-2;O60547 | 7;7 | 7;7 | 7;7 | GDP-mannose 4,6 dehydratase | GMDS | 1.81 |
| E9PEZ3;O60610;E9PHQ0;O60610-2;O60610-3;E7ERW8;H9KV28;E7EMV0;B4E2I7;E5RJ79 | E9PEZ3;O60610;E9PHQ0;O60610-2;O60610-3;E7ERW8;H9KV28;E7EMV0 | 48;48;46;46;46;45;45;30;8;2 | 48;48;46;46;46;45;45;30;8;2 | 48;48;46;46;46;45;45;30;8;2 | Protein diaphanous homolog 1 | DIAPH1 | 1.84 |
| F5H039;Q9NQX3;Q9NQX3-2;G3V582;H0YJ30 | F5H039;Q9NQX3;Q9NQX3-2;G3V582;H0YJ30 | 8;8;8;5;4 | 8;8;8;5;4 | 8;8;8;5;4 | Gephyrin;Molybdopterin adenylyltransferase;Molybdopterin molybdenumtransferase | GPHN | 1.84 |
| Q13867;K7ESE8;K7ES02;J3KSD8;J3KS79;K7ENH5 | Q13867;K7ESE8;K7ES02;J3KSD8 | 19;14;14;10;4;4 | 19;14;14;10;4;4 | 19;14;14;10;4;4 | Bleomycin hydrolase | BLMH | 1.85 |
| Q9P258 | Q9P258 | 29 | 29 | 29 | Protein RCC2 | RCC2 | 1.85 |
| P29144;Q5VZU9 | P29144;Q5VZU9 | 41;40 | 41;40 | 41;40 | Tripeptidyl-peptidase 2 | TPP2 | 1.85 |
| Q9H773;H3BSA6;H3BPN2 | Q9H773 | 14;6;4 | 14;6;4 | 14;6;4 | dCTP pyrophosphatase 1 | DCTPP1 | 1.85 |
| E5RHW4;O94905;E5RJ09;O94905-2;O94905-3 | E5RHW4;O94905 | 18;18;8;4;4 | 18;18;8;4;4 | 15;15;7;3;3 | Erlin-2 | ERLIN2 | 1.87 |
| I3L0K1;E7EQ34;O14653;O14653-2;I3L4Z6;O14653-3;I3L1K7;I3L3V4;I3NI02 | I3L0K1;E7EQ34;O14653;O14653-2;I3L4Z6;O14653-3;I3L1K7;I3L3V4 | 4;4;4;4;3;3;2;2;1 | 4;4;4;4;3;3;2;2;1 | 4;4;4;4;3;3;2;2;1 | Golgi SNAP receptor complex member 2 | GOSR2 | 1.88 |
| Q9UG63;Q9UG63-2;C9JHK9;C9JZV3 | Q9UG63;Q9UG63-2 | 19;19;9;9 | 19;19;9;9 | 19;19;9;9 | ATP-binding cassette sub-family F member 2 | ABCF2 | 1.88 |
| M0QXB5;O95571;M0QY80 | M0QXB5;O95571 | 8;8;1 | 8;8;1 | 8;8;1 | Persulfide dioxygenase ETHE1, mitochondrial | ETHE1 | 1.88 |
| P13693;J3KPG2;Q5W0H4;A0A0B4J2C3;P13693-2;E9PJF7;H0YCX0;Q56UQ5;Q9HAU6 | P13693;J3KPG2;Q5W0H4;A0A0B4J2C3;P13693-2;E9PJF7 | 12;11;11;11;9;8;3;3;1 | 12;11;11;11;9;8;3;3;1 | 12;11;11;11;9;8;3;3;1 | Translationally-controlled tumor protein | TPT1 | 1.89 |
| Q13162;H7C3T4;A6NJJ0;A6NG45 | Q13162;H7C3T4 | 17;11;5;5 | 13;7;5;5 | 13;7;5;5 | Peroxiredoxin-4 | PRDX4 | 1.89 |
| P49711;P49711-2 | P49711 | 9;4 | 9;4 | 9;4 | Transcriptional repressor CTCF | CTCF | 1.89 |
| Q9BPZ3;D6RA77 | Q9BPZ3;D6RA77 | 3;2 | 3;2 | 3;2 | Polyadenylate-binding protein-interacting protein 2 | PAIP2 | 1.89 |
| Q6NXE6-2;Q6NXE6;B4E1N1;F5GWV0;F5H4P3;H0YGL0;H0YH65;F5H2X2;F5H2K4;F5H3X1;F5H052;F5H6J3;F5GZP0 | Q6NXE6-2;Q6NXE6;B4E1N1;F5GWV0;F5H4P3 | 10;10;8;6;6;3;3;3;3;2;2;2;1 | 10;10;8;6;6;3;3;3;3;2;2;2;1 | 10;10;8;6;6;3;3;3;3;2;2;2;1 | Armadillo repeat-containing protein 6 | ARMC6 | 1.90 |
| Q5BKZ1;A0A0A0MRN4;Q5BKZ1-2;Q5BKZ1-3 | Q5BKZ1;A0A0A0MRN4 | 8;5;3;3 | 8;5;3;3 | 8;5;3;3 | DBIRD complex subunit ZNF326 | ZNF326 | 1.92 |
| P48506;A0A0C4DGB2;E1CEI4;D6RGF8;D6R959;H0Y9I7;D6REX4 | P48506;A0A0C4DGB2;E1CEI4 | 10;8;8;3;3;3;2 | 9;7;7;3;2;2;1 | 9;7;7;3;2;2;1 | Glutamate--cysteine ligase catalytic subunit | GCLC | 1.93 |
| P51572;P51572-2;C9JSP1;C9JQ75;C9J0M4;C9JMD7;C9JM14 | P51572;P51572-2;C9JSP1;C9JQ75;C9J0M4;C9JMD7 | 14;14;8;8;8;7;1 | 14;14;8;8;8;7;1 | 14;14;8;8;8;7;1 | B-cell receptor-associated protein 31 | BCAP31 | 1.94 |
| Q9NR30;Q9NR30-2 | Q9NR30;Q9NR30-2 | 33;29 | 33;29 | 31;27 | Nucleolar RNA helicase 2 | DDX21 | 1.95 |
| P16401 | P16401 | 17 | 15 | 15 | Histone H1.5 | HIST1H1B | 1.95 |
| Q8TDD1;Q8TDD1-2;H0YHH7;F8VRX4;H0YHZ9 | Q8TDD1;Q8TDD1-2 | 10;10;3;3;1 | 10;10;3;3;1 | 10;10;3;3;1 | ATP-dependent RNA helicase DDX54 | DDX54 | 1.95 |
| P00491;G3V5M2;G3V2H3;G3V393;Q8WW52-2;Q8WW52 | P00491;G3V5M2 | 22;15;6;4;1;1 | 22;15;6;4;1;1 | 22;15;6;4;1;1 | Purine nucleoside phosphorylase | PNP | 1.99 |
| Q92598;Q92598-2;Q92598-4;Q92598-3;A0A0A0MSM0;Q5TBM3;R4GN69 | Q92598;Q92598-2;Q92598-4;Q92598-3;A0A0A0MSM0 | 48;47;47;44;41;6;5 | 44;43;43;40;37;6;5 | 42;41;41;38;35;6;5 | Heat shock protein 105 kDa | HSPH1 | 1.99 |
| Q9BV57;Q9BV57-2;H7C382 | Q9BV57;Q9BV57-2 | 9;5;1 | 9;5;1 | 9;5;1 | 1,2-dihydroxy-3-keto-5-methylthiopentene dioxygenase | ADI1 | 2.00 |
| P00918;E5RID5;E5RK37 | P00918 | 8;3;3 | 8;3;3 | 8;3;3 | Carbonic anhydrase 2 | CA2 | 2.00 |
| P40763;P40763-3;G8JLH9 | P40763;P40763-3;G8JLH9 | 29;28;26 | 29;28;26 | 1;1;1 | Signal transducer and activator of transcription 3;Signal transducer and activator of transcription | STAT3 | 2.01 |
| P52292;J3QLL0;J3KS65 | P52292 | 18;7;7 | 18;7;7 | 18;7;7 | Importin subunit alpha-1 | KPNA2 | 2.01 |
| Q9BWD1;Q9BWD1-2;REV__H0Y3B8;REV__H3BLX7;REV__Q13635-4;REV__Q13635-3;REV__Q13635-2;REV__Q13635 | Q9BWD1;Q9BWD1-2 | 12;11;1;1;1;1;1;1 | 12;11;1;1;1;1;1;1 | 12;11;1;1;1;1;1;1 | Acetyl-CoA acetyltransferase, cytosolic | ACAT2 | 2.02 |
| G3V1D3;G3V180;Q9NY33;Q9NY33-4;E9PQ14;Q9NY33-2;E9PPK9;E9PKK8;E9PNX5;E9PQF2 | G3V1D3;G3V180;Q9NY33;Q9NY33-4 | 25;25;25;24;8;7;6;5;4;2 | 25;25;25;24;8;7;6;5;4;2 | 25;25;25;24;8;7;6;5;4;2 | Dipeptidyl peptidase 3 | DPP3 | 2.09 |
| P50416;P50416-2;H3BMD2;H3BP22;H3BUV7;H3BUJ0 | P50416;P50416-2 | 26;24;3;2;2;1 | 26;24;3;2;2;1 | 26;24;3;2;2;1 | Carnitine O-palmitoyltransferase 1, liver isoform | CPT1A | 2.13 |
| P05141 | P05141 | 19 | 6 | 6 | ADP/ATP translocase 2;ADP/ATP translocase 2, N-terminally processed | SLC25A5 | 2.14 |
| P42566;B1AUU8;P42566-2 | P42566;B1AUU8;P42566-2 | 16;12;9 | 16;12;9 | 16;12;9 | Epidermal growth factor receptor substrate 15 | EPS15 | 2.16 |
| Q99805 | Q99805 | 5 | 5 | 5 | Transmembrane 9 superfamily member 2 | TM9SF2 | 2.20 |
| Q9Y613;H3BVE7;J3KTH7 | Q9Y613 | 18;3;1 | 18;3;1 | 18;3;1 | FH1/FH2 domain-containing protein 1 | FHOD1 | 2.21 |
| A0A024QZP7;A0A087WY43;P06493;P06493-2;A0A087WZZ9;E5RIU6 | A0A024QZP7;A0A087WY43;P06493;P06493-2;A0A087WZZ9;E5RIU6 | 17;17;17;12;11;10 | 16;16;16;12;10;9 | 15;15;15;11;9;8 | Cyclin-dependent kinase 1 | CDC2;CDK1 | 2.23 |
| P02786;G3V0E5;F8WBE5;H7C3V5;Q9UP52-2;Q9UP52-3;Q9UP52 | P02786;G3V0E5 | 28;25;3;1;1;1;1 | 28;25;3;1;1;1;1 | 28;25;3;1;1;1;1 | Transferrin receptor protein 1;Transferrin receptor protein 1, serum form | TFRC | 2.28 |
| P63173;J3KT73;J3QL01;J3KSP2 | P63173;J3KT73;J3QL01 | 3;2;2;1 | 3;2;2;1 | 3;2;2;1 | 60S ribosomal protein L38 | RPL38 | 2.28 |
| Q13451;Q13451-2 | Q13451;Q13451-2 | 21;12 | 21;12 | 21;12 | Peptidyl-prolyl cis-trans isomerase FKBP5;Peptidyl-prolyl cis-trans isomerase FKBP5, N-terminally processed | FKBP5 | 2.34 |
| P52789;E9PB90 | P52789;E9PB90 | 27;26 | 23;22 | 23;22 | Hexokinase-2;Hexokinase | HK2 | 2.37 |
| A0A087WXR7;Q5TBB1;Q5TBB1-2;A0A087WZJ6 | A0A087WXR7;Q5TBB1;Q5TBB1-2 | 7;7;4;3 | 7;7;4;3 | 7;7;4;3 | Ribonuclease H2 subunit B | RNASEH2B | 2.44 |
| P49662;P49662-2;A0A087WZP8;E9PMT1;P51878-3;P51878-6;P51878-2;P51878;P51878-5;H0YD34;H7C0P5 | P49662;P49662-2;A0A087WZP8 | 15;13;12;6;3;3;3;3;3;1;1 | 15;13;12;6;3;3;3;3;3;1;1 | 15;13;12;6;3;3;3;3;3;1;1 | Caspase-4;Caspase-4 subunit 1;Caspase-4 subunit 2;Caspase | CASP4 | 2.44 |
| Q96KC8 | Q96KC8 | 5 | 5 | 5 | DnaJ homolog subfamily C member 1 | DNAJC1 | 2.45 |
| Q01469;I6L8B7;A8MUU1;REV__Q99880;REV__Q99879;REV__Q99877;REV__Q93079;REV__Q8N257;REV__Q5QNW6;REV__Q16778;REV__P62807;REV__P58876;REV__P57053;REV__P33778;REV__P23527;REV__P06899;REV__O60814;REV__Q5QNW6-2;REV__U3KQK0 | Q01469;I6L8B7 | 15;11;2;1;1;1;1;1;1;1;1;1;1;1;1;1;1;1;1 | 15;11;2;1;1;1;1;1;1;1;1;1;1;1;1;1;1;1;1 | 15;11;2;1;1;1;1;1;1;1;1;1;1;1;1;1;1;1;1 | Fatty acid-binding protein, epidermal | FABP5 | 2.48 |
| O43396;K7ER96;K7EML9;K7EPB7;K7EKG2;K7EME7 | O43396;K7ER96 | 14;13;6;2;1;1 | 14;13;6;2;1;1 | 14;13;6;2;1;1 | Thioredoxin-like protein 1 | TXNL1 | 2.68 |
| P15153;B1AH77;B1AH80;B1AH78 | P15153;B1AH77;B1AH80;B1AH78 | 9;8;8;7 | 5;5;5;4 | 5;5;5;4 | Ras-related C3 botulinum toxin substrate 2 | RAC2 | 2.72 |
| F5GZS6;P08195;J3KPF3;P08195-2;P08195-3;P08195-4;H0YFS2;F5H0E2;F5H867;F5GZI0;H0YFX4;F5GZR9 | F5GZS6;P08195;J3KPF3;P08195-2;P08195-3;P08195-4 | 24;24;23;23;23;23;9;8;5;5;3;1 | 24;24;23;23;23;23;9;8;5;5;3;1 | 24;24;23;23;23;23;9;8;5;5;3;1 | 4F2 cell-surface antigen heavy chain | SLC3A2 | 2.76 |
| P14550;Q5T621;V9GYG2;V9GYP9 | P14550 | 21;10;9;9 | 21;10;9;9 | 21;10;9;9 | Alcohol dehydrogenase [NADP(+)] | AKR1A1 | 2.83 |
| C9J8Q5;P51649;P51649-2 | C9J8Q5;P51649;P51649-2 | 10;10;10 | 10;10;10 | 10;10;10 | Succinate-semialdehyde dehydrogenase, mitochondrial | ALDH5A1 | 2.86 |
| O43719;Q5H919;Q5H918 | O43719 | 9;3;3 | 9;3;3 | 9;3;3 | HIV Tat-specific factor 1 | HTATSF1 | 2.97 |
| A0A0A6YYL4;P57737-3;I3L0X9;I3L3T0;Q9Y3D7;I3L1U7 | A0A0A6YYL4;P57737-3 | 17;17;2;2;2;1 | 17;17;2;2;2;1 | 2;2;2;2;2;1 | Coronin;Coronin-7 | CORO7-PAM16;CORO7 | 3.12 |
| P48651-2;P48651;P48651-3 | P48651-2;P48651;P48651-3 | 6;6;5 | 6;6;5 | 6;6;5 | Phosphatidylserine synthase 1 | PTDSS1 | 3.16 |
| Q06830;A0A0A0MSI0;A0A0A0MRQ5 | Q06830;A0A0A0MSI0 | 24;21;9 | 24;21;9 | 19;17;4 | Peroxiredoxin-1 | PRDX1 | 3.23 |
| Q01650;H0YJ95;A0A0C4DGL4;G3V4Z6;Q9UM01;Q92536 | Q01650;H0YJ95;A0A0C4DGL4;G3V4Z6;Q9UM01;Q92536 | 2;1;1;1;1;1 | 2;1;1;1;1;1 | 2;1;1;1;1;1 | Large neutral amino acids transporter small subunit 1;Y+L amino acid transporter 1;Y+L amino acid transporter 2 | SLC7A5;SLC7A7;SLC7A6 | 3.63 |
| E7EVW7;P14317;P14317-2;F8WDD5;F8WEZ6 | E7EVW7;P14317 | 9;9;3;1;1 | 9;9;3;1;1 | 9;9;3;1;1 | Hematopoietic lineage cell-specific protein | HCLS1 | 3.73 |
| P30740;P30740-2;C9J7N5;A0A0C4DGW9;O75830 | P30740 | 15;6;1;1;1 | 15;6;1;1;1 | 14;5;0;0;0 | Leukocyte elastase inhibitor | SERPINB1 | 3.74 |
| O95466-2;O95466;O95466-3;K7EK60;A0A0A0MR62;K7EJE6;K7ERL1 | O95466-2;O95466;O95466-3;K7EK60 | 16;14;14;11;7;6;1 | 16;14;14;11;7;6;1 | 16;14;14;11;7;6;1 | Formin-like protein 1 | FMNL1 | 3.84 |
| O14745;O14745-2;J3QRP6;J3QRA3 | O14745;O14745-2 | 11;6;5;2 | 11;6;5;2 | 11;6;5;2 | Na(+)/H(+) exchange regulatory cofactor NHE-RF1 | SLC9A3R1 | 3.91 |
| X6R433;A0A0A0MT22;P08575-2;P08575;M3ZCP1;A0A075B788;E9PKH0;M9MMK9 | X6R433;A0A0A0MT22;P08575-2;P08575;M3ZCP1;A0A075B788;E9PKH0 | 17;17;17;17;10;10;9;1 | 17;17;17;17;10;10;9;1 | 17;17;17;17;10;10;9;1 | Protein-tyrosine-phosphatase;Receptor-type tyrosine-protein phosphatase C | PTPRC | 3.96 |
| P27105;P27105-2;F8VSL7 | P27105 | 10;4;2 | 10;4;2 | 10;4;2 | Erythrocyte band 7 integral membrane protein | STOM | 4.21 |
| P49407-2;P49407;E9PM35;H0YET9;E7ESX4;P10523 | P49407-2;P49407;E9PM35 | 14;12;8;6;1;1 | 14;12;8;6;1;1 | 14;12;8;6;1;1 | Beta-arrestin-1 | ARRB1 | 4.40 |
| Q9NSI8;Q9NSI8-3;S6FRS6;Q9NSI8-2 | Q9NSI8;Q9NSI8-3;S6FRS6 | 13;13;12;6 | 13;13;12;6 | 12;12;11;6 | SAM domain-containing protein SAMSN-1 | SAMSN1 | 5.50 |
| E7EMG9;P33241;P33241-3;C9JU59;C9JDV1 | E7EMG9;P33241;P33241-3;C9JU59 | 8;8;8;5;2 | 8;8;8;5;2 | 2;2;2;2;2 | Lymphocyte-specific protein 1 | LSP1 | 8.59 |
| Q00534 | Q00534 | 14 | 13 | 13 | Cyclin-dependent kinase 6 | CDK6 | 8.89 |
| P20701;P20701-2;P20701-3;B4DQ77;H3BSV1 | P20701;P20701-2;P20701-3 | 13;13;11;1;1 | 13;13;11;1;1 | 13;13;11;1;1 | Integrin alpha-L | ITGAL | 10.84 |
| M0R0C1;O60234;M0QYG8;M0R1D2;M0QYJ8;M0QX47;M0QXC2 | M0R0C1;O60234;M0QYG8;M0R1D2;M0QYJ8 | 6;6;5;5;5;1;1 | 4;4;3;3;3;1;1 | 4;4;3;3;3;1;1 | Glia maturation factor gamma | GMFG | 10.96 |
| P31146;H3BRY3;H3BTU6;H3BRJ0;H3BNA2;H3BU76;H3BSL1 | P31146;H3BRY3 | 26;20;10;9;5;5;5 | 25;19;10;9;5;5;5 | 25;19;10;9;5;5;5 | Coronin-1A;Coronin | CORO1A | 15.32 |
| P52566;F5H3P3;H0YGX7;F5H6Q0;F5H2R5 | P52566;F5H3P3;H0YGX7;F5H6Q0 | 11;10;9;6;5 | 11;10;9;6;5 | 11;10;9;6;5 | Rho GDP-dissociation inhibitor 2 | ARHGDIB | 23.73 |
| P13796;Q5TBN3;P13796-2;Q5TBN5 | P13796 | 40;14;8;3 | 34;11;8;3 | 33;11;7;3 | Plastin-2 | LCP1 | 26.88 |
| A0A087WTF6;A0A087WX77;A0A087WWD4;A0A087WV75;P13591-1;P13591;H7BYX6;A0A087WTE4;P13591-4;P13591-3;P13591-5;P13591-6;A0A087WWJ5;A0A087X1V2;A0A087WVU1;A0A087WZS4;A0A0C4DGS4;A0A087WTR3 | A0A087WTF6;A0A087WX77;A0A087WWD4;A0A087WV75;P13591-1;P13591;H7BYX6;A0A087WTE4;P13591-4;P13591-3;P13591-5;P13591-6 | 21;21;21;21;21;21;16;16;16;16;13;11;10;5;5;3;2;1 | 21;21;21;21;21;21;16;16;16;16;13;11;10;5;5;3;2;1 | 21;21;21;21;21;21;16;16;16;16;13;11;10;5;5;3;2;1 | A0A087WTF6 | NCAM1 | 51.46 |
| Q9UI08;Q9UI08-2;Q9UI08-3;H0YJN0;G3V5F7;G3V3G2;G3V535;G3V314;A0A087WYI2;G3V2K5;H0YJL6 | Q9UI08;Q9UI08-2;Q9UI08-3;H0YJN0 | 20;20;17;10;7;6;6;6;6;5;3 | 20;20;17;10;7;6;6;6;6;5;3 | 20;20;17;10;7;6;6;6;6;5;3 | Ena/VASP-like protein | EVL | 52.05 |
| Q86UX7-2;Q86UX7;F5H1C6;H0YFT5;F5H3I6;G3V1L6;Q9BQL6-3 | Q86UX7-2;Q86UX7;F5H1C6 | 34;34;20;5;3;1;1 | 34;34;20;5;3;1;1 | 33;33;20;4;3;0;0 | Fermitin family homolog 3 | FERMT3 | 154.37 |

**Table S5. The top 10 upregulated proteins in HS5 after co-culture with KG1a.**

| Gene names | Protein descriptions | Mean SILAC ratio($\frac{\mathbf{Heavy(H)}}{\mathbf{Medium(L)}}$) |
| --- | --- | --- |
| SAMSN1 | SAM domain-containing protein SAMSN-1 | 5.50 |
| LSP1 | Lymphocyte-specific protein 1 | 8.59 |
| CDK6 | Cyclin-dependent kinase 6 | 8.89 |
| ITGAL | Integrin alpha-L | 10.84 |
| GMFG | Glia maturation factor gamma | 10.96 |
| CORO1A | Coronin-1A;Coronin | 15.32 |
| ARHGDIB | Rho GDP-dissociation inhibitor 2 | 23.73 |
| LCP1 | Plastin-2 | 26.88 |
| NCAM1 | A0A087WTF6 | 51.46 |
| EVL | Ena/VASP-like protein | 52.05 |
| FERMT3 | Fermitin family homolog 3 | 154.37 |

**Table S6. The top 10 downregulated proteins in HS5 after co-culture with KG1a.**

| Gene names | Protein descriptions | Mean SILAC ratio($\frac{\mathbf{Heavy(H)}}{\mathbf{Medium(L)}}$) |
| --- | --- | --- |
| DCD | Dermcidin;Survival-promoting peptide;DCD-1 | 0.04 |
| KRT1 | Keratin, type II cytoskeletal 1 | 0.04 |
| PLAT | Tissue-type plasminogen activator;  Tissue-type plasminogen activator chain A;  Tissue-type plasminogen activator chain B | 0.08 |
| ABI3BP;DKFZp667H216 | Target of Nesh-SH3 | 0.17 |
| SERPINB7 | Serpin B7 | 0.18 |
| INA | Alpha-internexin | 0.21 |
| SEPT3 | Neuronal-specific septin-3 | 0.21 |
| CRIP2 | Cysteine-rich protein 2 | 0.22 |
| QSOX1 | Sulfhydryl oxidase 1 | 0.22 |
|  |  |  |
